# Production of Antioxidant Molecules in *Polygonum aviculare* (L.) and *Senecio vulgaris* (L.) under Metal Stress: A Possible Tool in the Evaluation of Plant Metal Tolerance

**DOI:** 10.3390/ijms21197317

**Published:** 2020-10-03

**Authors:** Mirko Salinitro, Sara Hoogerwerf, Sonia Casolari, Alessandro Zappi, Dora Melucci, Annalisa Tassoni

**Affiliations:** 1 Department of Biological Geological and Environmental Sciences, University of Bologna, Via Irnerio 42, 40126 Bologna, Italy; mirko.salinitro2@unibo.it (M.S.); sara.hoogerwerf@studio.unibo.it (S.H.); 2 Department of Chemistry “G. Ciamician”, University of Bologna, Via Selmi 2, 40126 Bologna, Italy; sonia.casolari@unibo.it (S.C.); alessandro.zappi4@unibo.it (A.Z.); dora.melucci@unibo.it (D.M.)

**Keywords:** heavy metals, oxidative stress, antioxidant activity, polyphenols, flavonoids, photosynthetic pigments, population variability

## Abstract

Plants growing on heavy metal (HM)-polluted soils show toxicity symptoms, such as chlorosis and growth reduction, and undergo oxidative stress due to the formation of reactive oxygen species (ROS). Plants overcome oxidative stress by producing a wide range of antioxidant molecules, such as polyphenols and flavonoids. The aim of the present work was to study the accumulation of these molecules in response to increasing concentrations of Cd, Cr, Cu, Ni, Pb and Zn and to assess whether they can be used as a tool in assessing metal-related stress in *Polygonum aviculare* and *Senecio vulgaris*. On average, *P. aviculare* shoots accumulated lower amounts of metals than *S. vulgaris* shoots. The uptake of all six elements was correlated and proportional to their concentration in the nutrient solution (ρ > 0.9), with the bioaccumulation factor (BAF) being >1 for most of them. The present research demonstrated that 82% of the samples showed a good correlation (|ρ| > 0.5) between the level of polyphenols, flavonoids and antioxidant activity and the metal concentration in plant shoots, confirming that the metal stress level and production of phenolic compounds having antioxidant activity were strictly connected. Nonetheless, the mere quantification of these molecules cannot identify the type of metal that caused the oxidative stress, neither determine the concentration of the stressors. The five tested populations of each species did not show any specific adaptation to the environment of origin.

## 1. Introduction

Metal contamination is one of the most important contemporary environmental issues. Metals are naturally present in the soil, but, due to industrial and agricultural activities (such as smelting activities, vehicular traffic and use of sewage sludges, etc.), their concentration has increased to toxic levels in several areas [1,2]. Plants take up heavy metals (HMs) from contaminated soils together with other essential nutrients and accumulate them inside their tissues [3].

A basal HM tolerance can be found in all plant species, as they all coordinate a complex uptake/exclusion, transport/sequestration and detoxification system of such elements to protect sensitive organs from metal stress [4]. This system integrates different strategies for metal tolerance and detoxification [5]. At first, plants adopt a so-called avoidance strategy, aimed at reducing or excluding metal uptake from the soil [4]. When metal uptake cannot be avoided, plants activate tolerance mechanisms for detoxification, such as metal sequestration in different intracellular compartments [6]. These strategies may include metal transportation into the vacuole, binding to the cell wall and biosynthesis of compounds aimed at metal complexation (such as proline and metallothionein) [7,8].

The presence of HMs inside plant cells can disrupt the physiological and biochemical functions causing the formation of reactive oxygen species (ROS) [9,10,11]. ROS consist of radical and non-radical oxygen species, such as superoxide anion (O^2−^), hydrogen peroxide (H_2_O_2_) and hydroxyl radical (·OH), formed by the partial reduction of oxygen [12]. The increase in ROS concentration exposes plant cells to oxidative stress and may lead to lipid peroxidation, alteration of membranes and rupture of DNA strands [13]. Plants manage to overcome oxidative stress by activating several antioxidant defense mechanisms, among which the production of antioxidant enzymes and molecules able to prevent oxidative damage in cells [14,15,16]. Superoxide dismutase (SOD), peroxidase (POD) and catalase (CAT) are the most active antioxidant enzymes, while glutathione, carotenoids, ascorbate and polyphenols, and their subclass flavonoids, represent some of the non-enzymatic components of the plant detoxification system, acting as metal chelators or by directly scavenging ROS [17,18]. In particular, the antioxidant capacity of phenolic compounds is widely renowned, due to their redox properties, which allow them to act as reducing agents, stabilizing unstable metal forms inside plant cells [4]. Recent results evidenced that flavonoids can facilitate heavy metal tolerance in *A. thaliana* [19]. In particular quercetin was able to create complexes with copper [20] and uranium ions [21]. Similar results have been found by Salinitro et al. (2020) [22], who studied the concentration of phenolic compounds and antioxidant activity in relation to Zn and Ni uptake in *Stellaria media* (L.) Vill. In this herbaceous species, in fact, a strong correlation between metal shoot content and antioxidant content was demonstrated. The presence of metals and metalloids in plant organs does not only cause oxidative stress but also exerts a wide array of other negative effects, including reduced plant growth, low chlorophyll synthesis, a change of chlorophyll *a*/chlorophyll *b* ratio [23,24] and low photosynthetic activity [25] and transpiration rates [26].

The level of metal-induced stress in plants has usually been determined by measuring photosynthetic pigments reduction, together with SOD, POD and CAT enzyme activities [27]. Conversely, total polyphenol and flavonoid production have rarely been used as metal-related stress biomarkers. The main aim of the present study was to evaluate the effectiveness of using antioxidant activity, biomass and the production of polyphenols, and their sub-class flavonoids, as well as photosynthetic pigments as measuring tools to determine the metal stress levels in plants. For this purpose, two annual weeds, *Polygonum aviculare* (L.) and *Senecio vulgaris* (L.), were collected from five different locations: Bologna urban area (B), Milan urban area (M), Bologna woodland area (N), Milan woodland area (T) and Mt. Prinzera serpentine area (P). For each population, seedlings were grown in hydroponic culture with increasing concentrations of Cd, Cr, Cu, Ni, Pb and Zn and analyzed for various metal stress indicators. The research also aimed at identifying differences in metal uptake and target metabolites production among the populations adapted to different HM concentrations in their original habitats.

## 2. Results

### 2.1. Metal Accumulation

Both *P. aviculare* and *S. vulgaris* showed regular growth in hydroponics under control and treatment A conditions, while they exhibited metal toxicity symptoms at higher heavy metal (HM) concentrations (treatments B, C and D).

For all the tested metals, high correlations were found between the concentrations in the nutrient solution and those detected in plant shoots, with Spearman’s correlation coefficients (ρ) generally above 0.9.

**Figure 1:**
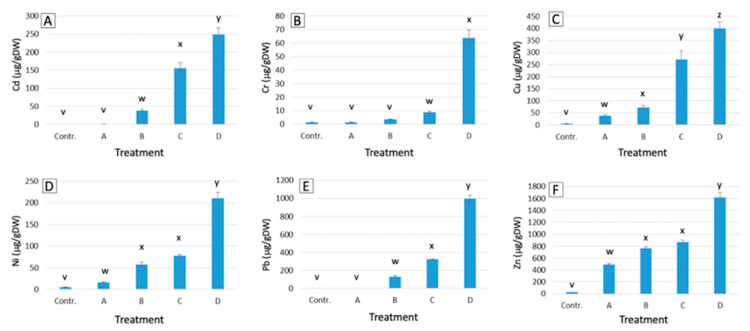
Metal contents in shoots of *P. aviculare* grown in hydroponics at increasing metal concentrations (from control to D, see Methods section). (**A**) Cd; (**B**) Cr; (**C**) Cu; (**D**) Ni; (**E**) Pb; (**F**) Zn. Bars represent the average of data coming from five populations analyzed in three biological replicates (*n* = 15). Different small letters (from v to z) above the bars indicate a statistically significant difference among the treatments (*p* < 0.05) calculated with *Dunn.test* using R software.

**Figure 2:**
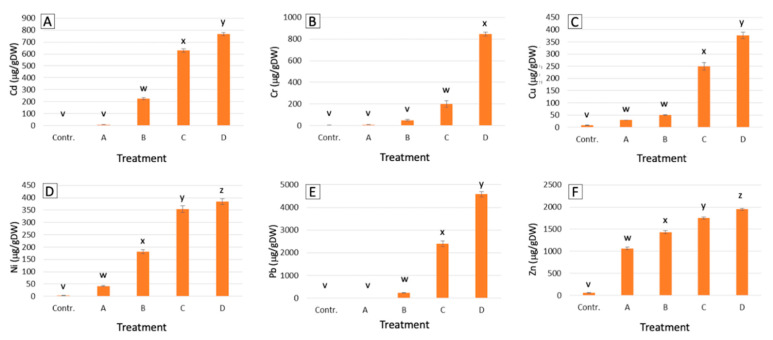
Metal contents in shoots of *S. vulgaris* grown in hydroponics at increasing metal concentrations (from control to D, see Methods section). (**A**) Cd; (**B**) Cr; (**C**) Cu; (**D**) Ni; (**E**) Pb; (**F**) Zn. Bars represent the average of data coming from five populations analyzed in three biological replicates (*n* = 15). Different small letters (from v to z) above the bars indicate a statistically significant difference among the treatments (*p* < 0.05) calculated with *Dunn.test* using R software.

On average, *P. aviculare* shoots (Figure 1) showed a lower metal uptake compared to that of *S. vulgaris* (Figure 2), with average contents, respectively, of 111 and 407 μg/g dry weight (μg/gDW) for Cd, 19 and 276 μg/gDW for Cr, 196 and 176 μg/gDW for Cu, 90 and 240 μg/gDW for Ni, 364 and 1805 μg/gDW for Pb and 937 and 1548 μg/gDW for Zn. Overall, the largest differences between the two species were detected for the Cr and Pb treatments, which resulted in, respectively, 22-times and 7-times higher average metal levels in *S. vulgaris* compared to *P. aviculare* (*p* < 0.01).

In *S. vulgaris* grown at high (C) and maximum (D) concentrations, the Pb content was above the set hyperaccumulation threshold (>1000 μg/gDW) [28], with, respectively, 2401 and 4580 μg/gDW of Pb accumulated in shoots (Figure 2). For Cd, the set hyperaccumulation threshold of 100 μg/gDW [28] was exceeded in *P. aviculare* C and D samples (respectively, 156 and 250 μg/gDW) (Figure 1) and in *S. vulgaris* B (medium), C and D samples (respectively, 226, 630 and 765 μg/gDW (Figure 2).

Depending on the plant species and treatment (A, B, C and D), metals were differentially absorbed and transferred to the shoots. This transfer is defined by a parameter called the bioaccumulation factor (BAF), calculated as the ratio between the metal concentration in plant shoots and that of the soil (in this case the hydroponic solution). For all the tested metals, the BAF values were generally higher for *S. vulgaris* (average of 13.6) with respect to *P. aviculare* (average of 11.3). The only exceptions were found in case of Cd and Cu, for which the transfer efficiency in *S. vulgaris* was lower than that in *P. aviculare* (average Cd BAF 19.6 and 32.5, and average Cu BAF 3.1 and 5.0, respectively) (Table 1).

Except for the Cr A and B treatments (BAF 0.7 and 0.9, respectively) and Pb A treatment (BAF 0.3) in *P. aviculare*, all BAF values were higher than 1, showing that the two studied species were able to concentrate metals in their aerial parts when grown in hydroponics. The present data also pointed out that, with increasing metal concentrations, the metal transfer decreased for Cd, Ni and Zn. Conversely, an opposite trend was observed for Cr and Pb, for which the BAF values increased with rising metal concentrations in the nutrient solution (Table 1). Cu was similarly absorbed at all concentrations, with the calculated BAF values quite constant in both plant species.

The increase in shoot metal concentration corresponded to an analogous increase in the dry weight/fresh weight (DW/FW) ratio (calculated from data in Supplementary Tables S1 and S2) in both *P. aviculare* and *S. vulgaris.* In both species, the DW/FW ratios ranged between 0.18 and 0.20 in treatment A (lowest metal concentration), increasing up to 0.28–0.32 in treatment D, the maximum metal concentration allowing plant growth (*p* < 0.01).

### 2.2. Effects of Metals on the Production of Antioxidant Metabolites and Photosynthetic Pigments

**Figure 3:**
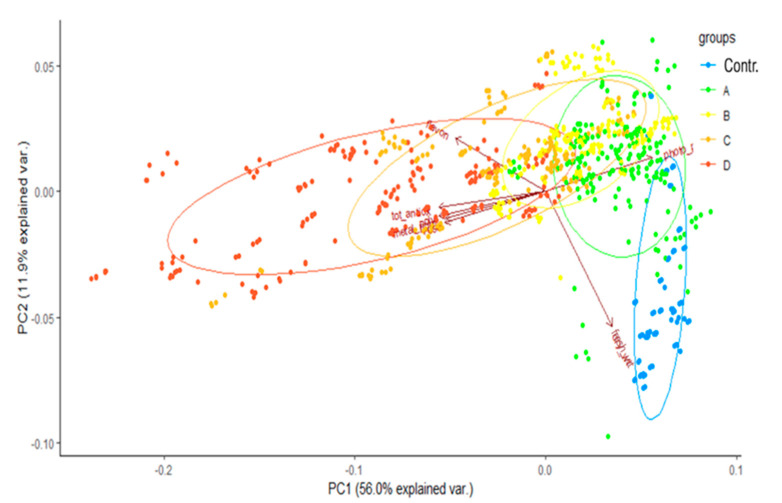
PCA analysis showing the grouping for *P. aviculare* data according to different metal treatments. The complete dataset reported in Supplementary Table S1 was used to perform the analysis by means of the *prcomp* function of the R package. Treatments: control, no metal; A, urban metal concentration; B, medium metal concentration, C, high metal concentration, D, maximum metal concentration allowing plant survival.

**Figure 4:**
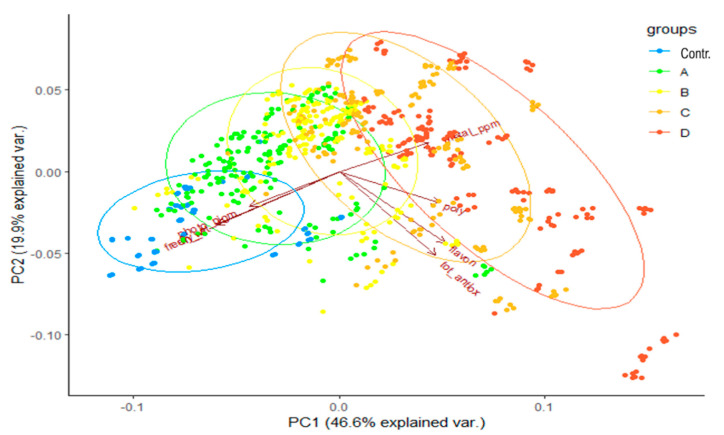
PCA analysis showing the grouping for *P. aviculare* data according to different metal treatments. The complete dataset reported in Supplementary Table S1 was used to perform the analysis by means of the *prcomp* function of the R package. Treatments: control, no metal; A, urban metal concentration; B, medium metal concentration, C, high metal concentration, D, maximum metal concentration allowing plant survival.

Overall, for all metals tested, increasing concentrations led to an increase in antioxidant activity and polyphenol and flavonoid amounts, while simultaneously the photosynthetic pigment levels and shoot FW decreased in both species, as shown by the explorative Principal Component Analysis (PCA) (Figure 3 and Figure 4). The PCA for *P. aviculare* (Figure 3), carried out including all dataset variables (Supplementary Table S1), showed that the data distribution and grouping of different treatments was mainly guided by shoot metal contents, by antioxidant capacity and by the photosynthetic pigment, polyphenol and flavonoid levels. The vertical distribution of the PCA was mainly guided by fresh and dry biomass, with in particular control treatments characterized by a high shoot biomass. However, the group division according to biomass values is not sharp; in fact, except for the control, which is clearly separated, all the other treatments are widely overlapping.

In *S. vulgaris*, the PCA (Figure 4) including all the dataset variables (Supplementary Table S2) showed, instead, that the data distribution was mainly guided by photosynthetic pigments level, shoot metal content and biomass, while antioxidant activity and the polyphenol and flavonoid levels had a minor role in sample grouping. The vertical distribution of the data given by the axis PC2 was mainly due to the polyphenol and flavonoid levels and antioxidant activity, which showed higher values for treatments C and D, even though the polyphenol and flavonoid data were quite dispersed in each dosage group.

Integration of all biological parameters indicated the metals and treatments that caused acute toxic effects in the studied species (data highlighted in yellow, Table 2). Toxicity was considered acute when a dramatic decrease in plant biomass and photosynthetic pigments levels was accompanied by an opposite sharp increase in polyphenol and flavonoid content. *P. aviculare* was in general less sensitive to HMs than *S. vulgaris*, since acute toxicity was found in only a few cases, in particular with maximum concentrations (D) of Cu, Pb and Zn. Instead, acute toxicity was detected more frequently in *S. vulgaris*, with high (Pb and Zn) and maximum (Cd, Cu, Ni, Pb and Zn) metal concentrations.

Compared to *S. vulgaris* plants, *P. aviculare* plants were in general characterized by higher photosynthetic pigments levels, antioxidant activity as well as polyphenol and flavonoid contents (Table 2), but had lower DW/FW ratios (Supplementary Tables S1 and S2). For all metals tested, the photosynthetic pigment levels were higher in control and treatment A samples (on average 224.6 μg/gFW) and progressively decreased with B, C and D dosages (on average 123.3 μg/gFW in D samples). Conversely, polyphenol and flavonoid content and antioxidant activity were higher in the C and D treatments (on average 9.4 mg gallic acid (GA) eq/gFW, 3.4 mg catechin (CAT) eq/gFW and 6.6 mg ascorbic acid (AA) eq/gFW, respectively, in samples D), decreasing about 40% in the control and A samples (on average 3.4 mg AA eq/gFW, 5.7 mg GA eq/gFW and 1.9 mg CAT eq/gFW, respectively, in the control samples) (*p* < 0.05). Moreover, fresh shoot biomass decreased from an average of 0.98 gFW in the control samples to the lowest value (average of 0.04 gFW) in treatment D samples (*p* < 0.01). Some exceptions to these general trends were also found. In *P. aviculare*, for example, the flavonoid concentration was almost constant at all Cd concentrations and most Cr concentrations (A, B and C).

Overall, in *S. vulgaris*, shoot FW (from 0.84 to 0.07 gFW) and photosynthetic pigment content (from 138.9 to 68.6 μg/gFW) steadily decreased with increasing metal concentrations, while an opposite trend was observed for shoot metal concentration (Table 2) and, to some extent, for the DW/FW ratio (Supplementary Table S2).

In *S. vulgaris*, polyphenol and flavonoid content and antioxidant activity showed higher values in treatments C and D (on average 1.4 mg GA eq/gFW, 0.9 mg CAT eq/gFW and 0.7 mg AA eq/gFW, respectively, in treatment D), while having lower concentrations in the control samples (on average 0.83 mg GA eq/gFW, 0.58 mg CAT eq/gFW and 0.53 mg AA eq/gFW, respectively) (*p* < 0.05). Polyphenol concentration tended to increase only at the highest metal concentration (D), being instead similar among the control, A and B treatments (*p* = 0.27). Photosynthetic pigment concentration slightly increased compared to the control at low levels of Cd, Cr, Ni and Pb, to diminish again at higher metal dosages (C and D).

The correlation between shoot metal concentration and the production of antioxidant metabolites and photosynthetic pigments were assessed using Spearman’s correlation coefficients (ρ). On the whole, considering both plant species, only 10% of the ρ values indicated a non-significant correlation (|ρ| < 0.300), whereas 8% showed a low correlation (0.301 < |ρ| < 0.500), 25% a good correlation (0.501 < |ρ| < 0.700) and 57% an excellent correlation (|ρ| > 0.701). HM shoot content was negatively correlated with photosynthetic pigment content and shoot biomass production, while it was generally positively correlated with polyphenol and flavonoid content and with antioxidant activity (Supplementary Table S3). In both species, the Zn and Cu samples showed the highest correlation with all the measured variables, while the Cd and Ni samples were those with the lowest correlation.

### 2.3. Population Differences

Under the present experimental conditions, the five populations (2 urban, 2 woodland and 1 serpentine) of both plant species showed a different behavior regarding shoot accumulation of the six tested metals.

**Figure 5:**
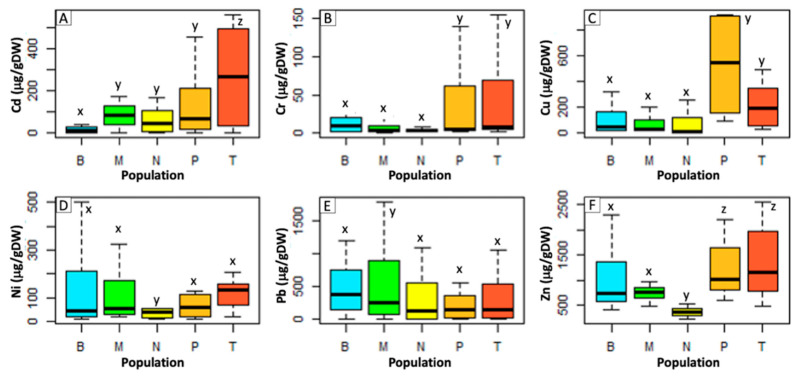
Shoot metal concentration in different *P. aviculare* populations. (**A**) Cd; (**B**) Cr; (**C**) Cu; (**D**) Ni; (**E**) Pb; (**F**) Zn. Populations: B, Bologna urban; M, Milan urban; N, Bologna woodland; T, Milan woodland; P, serpentine. Data represent the mean value of three biological replicates (*n* = 3). Different small letters (from x to z) above the bars indicate a statistically significant difference among the treatments (*p* < 0.05) calculated with *Dunn.test* using R software.

Regarding *P. aviculare* shoots (Figure 5), the Milan woodland samples (T) always showed significantly higher Cd, Cr, Cu, Ni and Zn concentrations compared to the other populations (*p* < 0.01). Similarly, the Mt. Prinzera serpentine population (P) showed 50–70% higher Cu levels with respect to all other samples (*p* < 0.01). The Bologna woodland population (N) generally accumulated the lowest concentration of all metals, even if statistically significant differences were detected only for Cr, Ni and Zn (*p* < 0.01). Interestingly, Zn was accumulated similarly by the Milan (M) and Bologna (B) urban populations, with an average content of 744 μg/gDW and 997 μg/gDW, respectively, which was 57% higher than those of N and P samples and 47% lower than the T sample content (*p* < 0.01) (Figure 5F).

**Figure 6:**
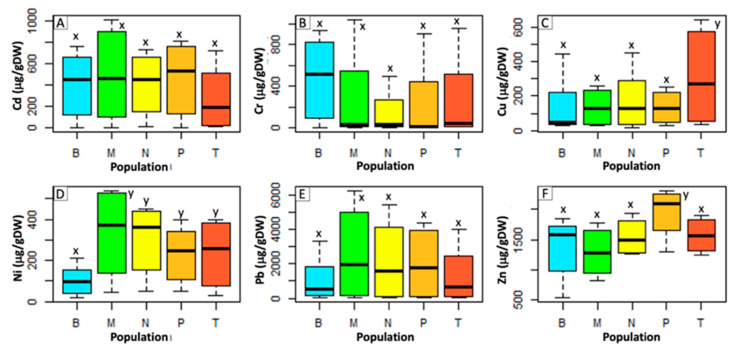
Shoot metal concentration in different *S. vulgaris* populations. (**A**) Cd; (**B**) Cr; (**C**) Cu; (**D**) Ni; (**E**) Pb; (**F**) Zn. Populations: B, Bologna urban; M, Milan urban; N, Bologna woodland; T, Milan woodland; P, Mt. Prinzera serpentine. Data represent the mean value of three biological replicates (*n* = 3). Different small letters (from x to z) above the bars indicate a statistically significant difference among the treatments (*p* < 0.05) calculated with *Dunn.test* using R software.

*S. vulgaris* populations (Figure 6), conversely, showed similar uptake levels for all the tested metals. The only exceptions were represented by the Bologna urban plants (B), showing a shoot Ni content on average 64% lower (100 μg/gDW) compared to the other samples (274 μg/gDW) (*p* < 0.01), and by the Mt. Prinzera serpentine plants (P), with a 26% more Zn (1953 μg/gDW) content with respect to the other samples.

Some differences among the populations were also detected when comparing the antioxidant activity as well as polyphenol and flavonoid contents. In *P. aviculare*, the two woodland populations (N and T) both had a higher polyphenol content (on average 6.1 mg GA eq/gFW) but lower flavonoid content and antioxidant activity (1.5 mg CAT eq/gFW and 2.6 mg AA eq/gFW) compared to the other populations (on average 4.1 mg GA eq/gFW, 3.5 mg CAT eq/gFW and 3.8 mg AA eq/gFW) (*p* < 0.01). Conversely, the two urban populations (B and M) showed opposite trends, with a low polyphenol content but high flavonoid content and antioxidant activity.

Some differences were also present among the five *S. vulgaris* populations, but no consistent trends were detected. The Milan urban population (M) showed higher values for polyphenol and flavonoid content and antioxidant activity compared to all other samples (*p* < 0.01). N and T plants had overall a higher polyphenol content than the other populations (*p* < 0.01), while the B, N and T populations had lower flavonoid levels compared to the P and M populations (*p* < 0.01). No differences in shoot biomass were reported among the populations for both *P. aviculare* and *S. vulgaris* (*p >* 0.05).

## 3. Discussion

Our results pointed out that the amounts of the six tested metals, detected in the shoots of both species, were directly proportional to their concentration in the hydroponic culture media (Figure 1 and Figure 2). As a consequence of this proportional absorption, the studied species could be considered good indicator plants, confirming previous results that proved *S. vulgaris* to be a viable Ni indicator in different soil environments [29]. According to the present data, *P. aviculare* was more efficient than *S. vulgaris* in preventing metal absorption, going towards an excluder behavior, especially in the presence of Cd and Pb. Indeed, all metals were detected at lower levels in *P. aviculare* compared to *S. vulgaris* shoots (from −60% for Zn up to −93% for Cd), with the exception of Cu, which was instead similarly absorbed by both species (on average 195 μg/gDW and 176 μg/gDW, respectively, for *P. aviculare* and *S. vulgaris*) (Figure 1 and Figure 2). These data are in agreement with previous studies on *Arabidopsis thaliana,* which demonstrated that the Cu requirement is strictly regulated by plants to prevent uncontrolled uptake from the substrate [30]. Our results led to the hypothesis that a similar behavior is also present in *P. aviculare* and *S. vulgaris*.

The hyperaccumulation thresholds set by van der Ent et al. [28] on the basis of a global database of hyperaccumulator plants were exceeded for some tested metals. In particular, the B, C and D samples of *S. vulgaris* accumulated on average above 500 μg/gDW of Cd (threshold > 100 μg/gDW) and in the C and D samples the shoot Pb content was on average above 5000 μg/gDW (threshold > 1000 μg/gDW). Similarly, in the *P. aviculare* shoots, the Cd content of the C and D samples was above 250 μg/gDW (Figure 1 and Figure 2). Despite these results, the two selected plant species cannot yet be considered hyperaccumulators, as under the present experimental conditions, they were not cultivated in soil, which is one of the mandatory requirements to define the hyperaccumulation capacity of a species [28]. Furthermore, the hyperaccumulation threshold was only exceeded when the highest metal concentrations were added to the hydroponic medium, a condition that may have caused an uncontrolled breakthrough of metals due to a generalized physiological disruption of the plant cells [3].

In both species, all metals (except for a few Cr and Pb treatments in *P. aviculare*) showed a shoot bio-accumulation factor (BAF) above 1, demonstrating the ability of these plants, in hydroponic conditions, to concentrate metal ions within their aerial parts. Similar results are usually unachievable when these plants are cultivated in the soil, where metals are less bioavailable. In fact, data on *P. aviculare* and *S. vulgaris* collected from natural soils showed that these species were not capable of concentrating HMs in the shoots, having in most cases BAFs below 0.3 [29].

In both plant species, the BAFs decreased with increasing Cd, Ni and Zn concentrations in the hydroponic solution (ranging on average from 29.8 in A treatments to 8.1 in D treatments), demonstrating a controlled absorption mechanism for these elements to limit their accumulation at toxic concentrations. Conversely, increasing doses of Cr and Pb led to higher BAFs in both species (on average from 1.6 for A treatments to 9.7 for D treatments). This trend can be explained by a passive uptake mechanism, as already shown for some non-essential elements, such as Cr, for which the absence of specific cellular transporters was also confirmed [31]. The BAFs remained almost constant for the Cu treatments, confirming again the strict uptake regulation of this ion.

The two species, besides having different HM accumulation capacities, also showed a diverse response to metal-related oxidative stress. *P. aviculare*, for example, showed an average 1.7-, 2.1- and 2.5-fold increase, respectively, compared to the controls for polyphenol, flavonoid and antioxidant activity levels in D-treated plants. On the other hand, a greater antioxidant response was generally detected in *S. vulgaris*, which showed a 2.0-, 5.3- and 8.2-fold increase, respectively, compared to the controls for polyphenol, flavonoid and antioxidant activity levels in D-treated plants (Table 2 and Supplementary Tables S1 and S2).

A direct effect of increasing metal concentrations on antioxidant metabolites production has been described in other plant species, such as *Capsicum annuum* and waterlily [4,18]. Lavid et al. [32] demonstrated that increasing Cd accumulation in *Nymphaea alba* leaves was accompanied by increasing polyphenol and peroxidase activity levels. Polyphenols, in fact, can act as scavengers of HMs, thanks to their capacity to form insoluble complexes with divalent and trivalent cations, reducing their cellular concentrations [32]. Similar results were also reported for *Stellaria media* under Zn and Ni stress [22] and for tomato under Cu and Pb stress [14], which showed higher values of polyphenols and antioxidant activity when the metal content inside plant organs increased. Similarly, Kidd et al. (2001) demonstrated that flavonoid biosynthesis is enhanced in *Zea mays* plants under Al toxicity [33], as well as in *Stellaria media* under Zn and Ni toxicity [22].

The present results pointed out that, under hydroponic growth conditions, 82% of the treatments showed a good or excellent correlation (0.501 < |ρ| < 1) between polyphenol, flavonoid and antioxidant activity levels and the metal concentration in plant shoots, confirming that the level of metal-related oxidative stress and the amount of produced secondary metabolites having antioxidant activity were strictly connected. However, previous reports demonstrated that the production of different types of polyphenols, flavonoids and other antioxidant molecules is rather ubiquitous in plants and can be stimulated by several stressors, among which are HMs, drought, UV radiation, pathogens and others [18,34]. For this reason, it could be difficult to infer a direct relation between metal uptake and antioxidant metabolites production when plants are grown under environmental conditions, such as in the open field, where several stressors may act simultaneously.

The occasional lack of linearity of these metal–metabolite relations can be misleading when antioxidant activity and polyphenol and flavonoid content are used to quantify the HM stress levels of plants. In the case of the two studied species, these correlations were sometimes represented by logarithmic (e.g., Cu treatments in woodland populations) or exponential (e.g., Pb treatments in woodland populations) dose–response curves. In the case of a logarithmic curve, the plant immediately reacts to low HM concentrations, producing a high amount of antioxidant molecules that rapidly reach a plateau, after which increasing the HM concentrations do not further stimulate their production. In the opposite case of an exponential curve, the plant does not react to increasing HM concentrations up to a specific threshold, after which a sudden production of antioxidant metabolites occurs. Nonetheless, if coupled with other markers, like shoot biomass and photosynthetic pigments content, the polyphenol and flavonoid levels and antioxidant capacity can contribute in evaluating the metal stress status of a plant.

Several research papers reported that HMs strongly affect photosynthetic processes, causing both a reduction of photosynthetic pigments production and plant growth [26,35]. Severe toxic effects of Cr on *N. alba* leading to photosynthetic pigments reduction have been reported. In particular, when the plant was exposed to 200 μM Cr for twelve days, an 81.3% and 61.4% decrease, respectively, of chlorophyll *a* and *b* was detected [36]. In addition, *Plantago major* [37] and *Brassica napus* [38] grown in the presence of Pb showed a decline in photosynthetic pigments production and a lower biomass compared to the control treatments.

In the present study, the drastic reduction of photosynthetic pigment levels and shoot biomass in combination with a simultaneous sharp increase in the production of polyphenols and flavonoids were considered biomarkers of acute HM stress conditions, evidencing which metals at which concentration caused the highest toxicity in the two studied species (Table 2). This situation occurred more frequently in *S. vulgaris* than in *P. aviculare,* probably due to a greater plant sensitivity, caused by the higher capacity of *S. vulgaris* to uptake and translocate metals to shoots. Overall, the most toxic element, even when supplied at very low concentrations (treatments A and B), was Cd, as previously reported for some agricultural crops (e.g., barley), in which a 2 to 20-fold higher toxicity of Cd with respect to other HMs was reported [39].

The present research also aimed at assessing whether HM uptake varies in different *P. aviculare* and *S. vulgaris* populations, collected from urban, woodland and serpentine environments. This pre-adaptation has been observed in *Paspalum distichum* and *Cynodon dactylon* populations grown on mine tailings and non-contaminated soils [40]. The collected data on the five *P. aviculare* and *S. vulgaris* populations did not show consistent patterns related to their original habitat (Figure 5 and Figure 6).

Although no pre-adaptation could be demonstrated for the five different populations in relation to metal uptake, different trends were detected among *P. aviculare* accessions when considering the production of plant metabolites having antioxidant activity. The two urban populations (B and M) showed similar trends, which were opposite to those of woodland populations (T and N). Regarding the production of secondary metabolites, *S. vulgaris* did not show differences among populations that could reflect pre-adaptation to metal stress in their original environment. This could be due to the fact that *S. vulgaris* may need a longer period than *P. aviculare* to differentiate ecotypes, given its wind-dispersion strategy. It was in fact reported that long distance dispersion (such as wind dispersion) favors genetic mixing, maintaining low differences among populations, as also demonstrated for several tropical species [41].

## 4. Materials and Methods

### 4.1. Species Selection

**Figure 7:**
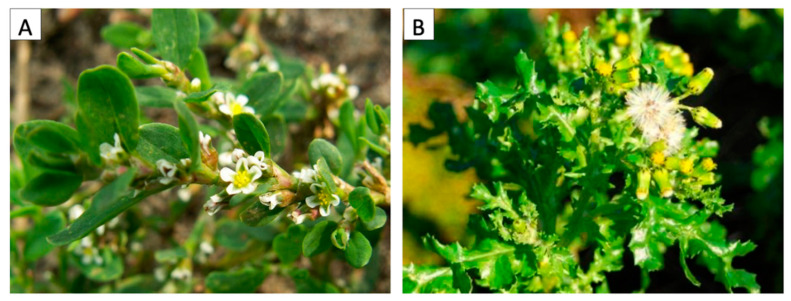
(**A**) *Polygonum aviculare* (L.), and (**B**) *Senecio vulgaris* (L.). Photo by Mirko Salinitro.

*Polygonum aviculare* (Figure 7A), an annual plant belonging to the Polygonaceae family, is a cosmopolitan species growing in several disturbed habitats, in particular in frequently trampled areas. It germinates in spring and continuously flowers during the hot season to finally produce seeds in autumn. It has creeping stems up to 45–50 cm long and dark-green oval leaves with small whitish flowers growing at the axil of each, followed by small triangular nuts. *Senecio vulgaris* (Figure 7B), is an annual plant belonging to the Asteraceae family. It also has a cosmopolitan distribution, growing on disturbed soils, like arable fields and wasteland. It germinates in autumn and/or spring, depending on the latitude, and flowers all year long. The plant is erect, 35–40 cm tall, and well branched. It produces several yellow flower heads, quickly followed by hairy seeds adapted to wind dispersion.

For both plant species, five different populations were collected from five different locations: Bologna urban area (B), Bologna woodland area (N), Milano urban area (M), Milano woodland area (T) and Mt. Prinzera serpentine area (P), following the sampling plan as in Salinitro et al. [29].

These populations are adapted to different soil heavy metal (HM) levels: the two urban populations (B and M) are adapted to high levels of several HMs, especially Zn, Cu and Pb; the Mt. Prinzera serpentine population (P) tolerates high levels of Ni and Cr; and the two woodland populations (N and T) are adapted to low-HM soil [29].

### 4.2. Plant Cultivation

For germination, seeds were placed on humid coarse quartz sand in transparent plastic boxes. *P. aviculare* seeds require light and warm temperature to germinate; therefore, the boxes were kept at 20 °C with a 16/8 h light/dark cycle. Conversely, for *S. vulgaris* seeds, which geminate better in the dark at low temperatures, the boxes were kept for three days in the dark at 10 °C, and then at 20 °C with a 16/8 h light/dark cycle until complete germination.

After the first leaf development (at about 10 days), seedlings were transferred from the germination boxes to 5 cm in diameter plastic containers (5 plants per pot). These final pots were immersed in trays filled with 200 mL of half strength Hoagland’s nutrient solution [42], so as to cover 1/3 of the pot. All chemicals were purchased from Merck (Darmstadt, Germany).

The nutrient solution contained: 2 mM KNO_3_, 2 mM Ca(NO_3_)_2_ · 4H_2_O, 0.5 mM NH_4_NO_3_, 0.5 mM MgSO_4_ · 7H_2_O, 0.25 mM KH_2_PO_4_, 50 µM KCl, 25 µM H_3_BO_3_, 2 µM MnCl_2_ · 4H_2_O, 2 µM ZnSO_4_ · 7H_2_O, 0.5 µM CuSO_4_ · 5H_2_O, 0.075 µM (NH_4_)_6_Mo_7_O_24_ · 4H_2_O, 0.15 µM CoCl_2_ · 6H_2_O, 0.05 µM NiCl_2_ · 6H_2_O and 40 µM Fe-EDTA. The solution pH was adjusted to 5.8 ± 0.2 with 1 M NaOH. 

Each different HM treatment was obtained by supplying the proper amount from a concentrated stock solution (0.1 M CdCl_2_ · 2.5 H_2_O, 1M CrCl_3_ · 6H_2_O, 1M CuSO_4_ · 5H_2_O, 1M NiCl_2_ · 6H_2_O, 1M Pb-EDTA and 1M ZnSO_4_ · 7H_2_O), according to the experimental plan in Table 3.

For treatment A, the total concentration of each metal corresponded to that found in urban soils [29]. These values were multiplied by different factors (e.g., for Zn by 2, 4 and 10), according to the toxicity of each element up to concentration D, the maximum still allowing plant growth and development. Plants were cultivated for four weeks: the first week after the transplant in half strength Hoagland’s nutrient solution without any metal, and the following three weeks in half-strength Hoagland’s nutrient solution spiked with different metal concentrations (Table 3). To avoid nutrient depletion, the solutions were totally replaced every week. Plants were cultivated in a growth chamber equipped with four SON-T and four HPI-T Plus lamps (Philips, Amsterdam, Netherlands) at a constant temperature of 21.5 ± 0.5 °C, with a 16/8 h light/dark cycle and a photosynthetic photon-flux density of 350 μmol/m^2^/sec.

### 4.3. Sample Collection and Preparation

At the end of the fourth week, three plants for each treatment of each of the five populations were harvested. Plant shoots were rinsed with deionized water and dried with paper towels before weighting (grams of fresh weight, gFW). Fresh samples were then ground with a mortar and pestle in liquid nitrogen to obtain a homogeneous powder that was used for spectrophotometric analysis. Powder aliquots (1 gFW) were dried at 105 °C for 24 h to calculate the dry weight (gDW) and then used for metal quantification.

### 4.4. Heavy Metal Quantification

Heavy metal quantification was carried out by pre-digesting samples (about 0.1 g of dry powder) at room temperature with 2 mL 70% (*v*/*v*) HNO_3_ for 1 day, followed by digesting for 1 h at 70 °C and 1 h at 125 °C according to a modified method by Huang and Schulte [43]. After digestion, samples were brought up to 10 mL with deionised water. Five replicates of the reference material (apple leaves, NIST^®^ SRM^®^ 1515, purchased from Merck, Darmstadt, Germany) and blanks (only 70% *v*/*v* HNO_3_) were subjected to the same procedure and added to the samples as quality control. Recovery rates of the six metals were within ±5% of the target concentrations of the certified reference material. The limits of quantification of the analyzed elements were 0.4, 0.4, 0.5, 0.3, 0.2 and 0.4 µg/g for Cd, Cr, Cu, Ni, Pb and Zn, respectively. A metal concentration analysis was performed by inductively coupled plasma mass spectrometry (ICP-MS) using an Elan 9000 DRCe detector (Perkin Elmer, Waltham, MA, USA). Data were expressed as μg of metal per g sample dry weight (μg/gDW).

### 4.5. Spectrophotometric Analysis

For the quantification of antioxidant activity and total polyphenol and flavonoid content, 0.1 gFW of the frozen ground sample was extracted with 1 mL of 95% (*v*/*v*) methanol and shaken overnight at room temperature. The supernatant was then recovered after centrifugation at 12,000 rpm for 5 min. All the samples were analyzed in a VersaMax™ Microplate Reader (Molecular Devices, San Jose, CA, USA) spectrophotometer.

The total flavonoids quantification assay was performed as described in Ferri et al. [44]. Sample absorbance was detected at 510 nm and data were expressed as mg catechin (CAT) equivalent/gFW, by means of a dose–response calibration curve performed with increasing concentrations of CAT, from 2 to 14 μg/mL.

For total polyphenols, the Folin–Ciocalteu assay [44] was used. Sample absorbance was detected at 765 nm and the data were expressed as mg gallic acid (GA) equivalent/gFW, using a dose–response calibration curve, with increasing the concentrations of GA from 0 to 150 μg/mL.

Finally, total antioxidant quantification was achieved using the 2,2’-azino-bis(3-etilbenzotiazolin-6-sulfonic) acid (ABTS) assay [44]. Sample absorbance was measured at 734 nm and the data were expressed as mg ascorbic acid (AA) equivalent/gFW. The calibration curve was performed with increasing the concentrations of AA from 0 to 80 μg/mL.

Quantification of photosynthetic pigments was performed following a modified method described by Radwan et al. [45], in which 0.1 gFW of frozen grinded sample was extracted with 85% (*v*/*v*) acetone and mixed twice for 30 s. The samples were then centrifuged at 2500 rpm for 5 min and the supernatant recovered. The quantification of chlorophyll *a*, *b* and carotenoids was carried out by a microplate-reader spectrophotometer at three different wavelengths: 663 nm, 644 nm and 452.5 nm. The obtained absorbance values were processed to give the pigment concentrations (μg/gFW) as follows:

Chlorophyll *a* = 10.3 · Abs_663_ − 0.98 · Abs_644_

Chlorophyll *b* = 19.7 · Abs_644_ − 3.87 · Abs_663_

Carotenoids = 4.2 · Abs_452.5_ − [(0.0264 · chl-*a*) + (0.426 · chl-*b*)]

### 4.6. Data Analysis

For each treatment of each population, three biological replicates were analyzed in two technical replicates (*n* = 3). Statistical analyses were performed using R software (version 3.1.5), R Core Team, Vienna, Austria. The differences in metal uptake and metabolite production were evaluated among the five different plant populations, as well as the different metal treatments and dosage. Data were tested for normality using the Shapiro–Wilk normality test, and for homogeneity using Levene’s test for homogeneity of variance with the default parameters from the package *car* [46]. Since the data were non-parametric, the Kruskal–Wallis test, followed by Dunn’s multiple pairwise comparison post-hoc test from the *dunn.test* package [47], was used to evaluate the differences among the compared groups with *p*-values reported in brackets in the Results section. Spearman’s correlation coefficients (ρ) were calculated to determine the presence of significant relationships between metal dosage (for each metal) and antioxidant activity, shoot biomass, and contents of polyphenols, flavonoids, and photosynthetic pigments. Spearman’s correlation coefficients can be divided into four classes: |ρ| < 0.300 = no correlation; 0.301 <|ρ| < 0.500 = low correlation; 0.501 < |ρ| < 0.700 = good correlation; and |ρ| > 0.701 = excellent correlation (|ρ| indicating the absolute value of the Spearman’s correlation coefficient). The relations between metal uptake and the other measured parameters were described using linear regression models (ρ and *p*-values are reported in brackets in the Results section). Principal component analysis (PCA) was performed with the function *prcomp*, using the default values. Graphical elaborations where performed using the R package *ggpubr* [48] and Microsoft Excel 2010.

## 5. Conclusions

The present study demonstrated that *P. aviculare* and *S. vulgaris* showed linear uptake of Cd, Cr, Cu, Ni, Pb and Zn with increasing metal concentrations when cultivated in hydroponic conditions. Consequently, the two studied species can be suitable metal indicators in several environments, also given their widespread presence and easy recognition.

For the highest concentration of the Cd and Pb treatments, the conventional hyperaccumulation threshold was largely exceeded by the two species, even though in these conditions the plants showed severe oxidative stress damage, highlighting their incapability to hyper-accumulate metals. *P. aviculare* demonstrated to be more tolerant to high HM levels in hydroponic solutions than *S. vulgaris,* suggesting its possible use for phytostabilization purposes.

Differences in HM accumulation and antioxidant metabolite production were detected among the five *P. aviculare* populations, being instead absent among the *S. vulgaris* populations. However, these differences did not indicate a possible pre-adaptation of each population to their original environment.

Our study demonstrated that the flavonoid, polyphenol and antioxidant activity levels, as well as the photosynthetic pigments production and shoot biomass, were in most cases correlated with shoot metal content and, also, that when these variables are taken in consideration all together, they can be efficiently used as a joint marker of HM stress in plants. On the other hand, the single quantification of these molecules cannot give information about the type of metal causing oxidative stress nor about the stressor concentration, as a high dosage of a lowly toxic metal may give similar effects as a low dosage of a highly toxic metal.

In conclusion, the tested parameters can be effectively used to evaluate metal sensitivity and toxicity in plants, but only when coupled with the quantification of metal trace elements inside plant tissues.

## Figures and Tables

**Table 1:** Bioaccumulation factors in *P. aviculare* and *S. vulgaris.* Each value is the average of the data coming from five populations tested in three biological replicates (*n* = 15). A 15% SE error applies to all data.

Metal	Treatment	BAF *P. aviculare*	BAF *S. vulgaris*
**Cd**	A	46.3	39.5
	B	37.4	20.4
	C	29.5	11.2
	D	16.9	7.7
**Cr**	A	0.7	4.0
	B	0.9	5.2
	C	1.8	10.7
	D	5.6	18.3
**Cu**	A	4.5	3.6
	B	4.4	3.1
	C	5.5	2.9
	D	5.9	3.1
**Ni**	A	13.7	34.5
	B	8.7	25.5
	C	5.3	11.2
	D	2.7	9.9
**Pb**	A	0.3	1.6
	B	2.4	4.4
	C	4.5	5.3
	D	6.2	8.8
**Zn**	A	26.9	48.0
	B	21.1	39.1
	C	11.9	24.1
	D	8.9	10.7

**Table 2:**
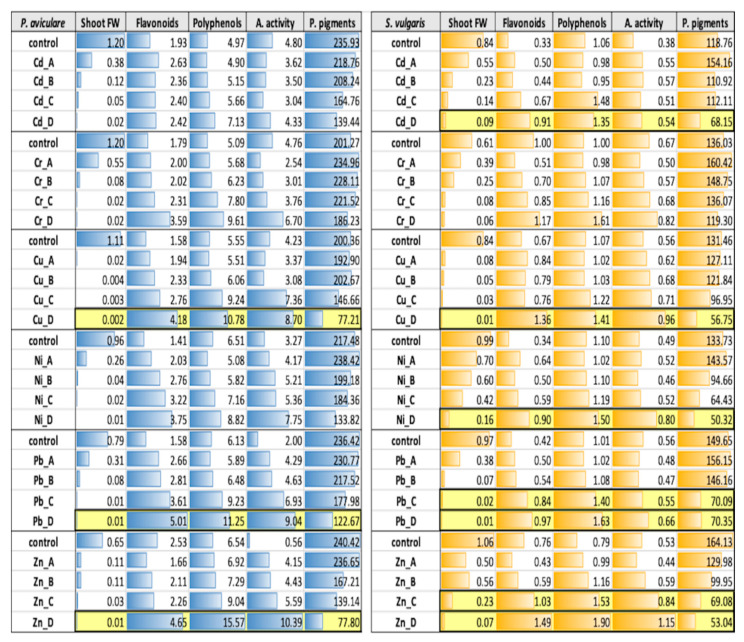
Fresh weight (gFW), antioxidant activity (mg ascorbic acid eq/gFW), flavonoid (mg catechin eq/gFW), polyphenol (mg gallic acid eq/gFW) and photosynthetic pigment contents (μg/gFW) in *P. aviculare* and *S. vulgaris* shoots. Each value is the average of the five populations, tested in three biological replicates (*n* = 15). A 15% SE error applies to all data. Yellow bars indicate concentrations giving acute toxic effects. Blue and orange bars highlight the specific data variability with respect to the highest value of each column variable. A. activity: antioxidant activity, P. pigments: photosynthetic pigments content (sum of chlorophyll *a*, chlorophyll *b* and carotenoid levels).

**Table 3:** Metal concentration of the nutrient solutions for each tested element. Treatments: Control, no metal (except the ones already present in the basic Hoagland’s solution indicated in brackets); A, mean urban concentration [29]; B, medium concentration; C, high concentration; and D, maximum concentration allowing plant growth.

Treatment	Zn (mM)	Pb (mM)	Cu (mM)	Ni (mM)	Cr (mM)	Cd (μM)
**Control**	(2 µM)	0	(0.5 µM)	(0.05 µM)	0	0
**A**	0.28	0.025	0.13	0.025	0.036	0.0945
**B**	0.56	0.25	0.26	0.125	0.18	9.45
**C**	1.12	1.25	0.65	0.250	0.36	47.25
**D**	2.80	2.50	1.30	0.625	0.90	94.50

## Supplementary Materials

The following are available online at https://www.mdpi.com/1422-0067/21/19/7317/s1. Supplementary Table S1: complete dataset of *Polygonum aviculare*; Supplementary Table S2: complete dataset of *Senecio vulgaris*. Supplementary Table S3: complete table of Spearman’s correlation coefficients (ρ) for *P. aviculare* and *S. vulgaris*.

## Abbreviations

AA	ascorbic acid
ABTS	2,2’-azino-bis(3-etilbenzotiazolin-6-sulfonic) acid
CAT	catechin
GA	gallic acid
HM	heavy metal
ROS	reactive oxygen species
