# Supplementary material for: Production of Antioxidant Molecules in Polygonum aviculare (L.) and Senecio vulgaris (L.) under Metal Stress: A Possible Tool in the Evaluation of Plant Metal Tolerance
Source: Int J Mol Sci. 2020 Oct 3;21(19):7317. doi: 10.3390/ijms21197317 (PMC7582341; doi:10.3390/ijms21197317)
Supplement: Supplementary file 1 [file ijms-21-07317-s001.zip › Supplementary Table S1 dataset Polygonum.pdf]

Supplementary Table S1. Complete dataset of *Polygonum aviculare.*

Sample	populat	treatment	factor_conc	Conc_met	shoot_fresh_wt	dry_wt	metal_ppm_shoot	flavon	poly	act_antiox	photo_pigm
B1control	B	control	0	0	1.3849	0.1637	5.714	1.7918	5.1222	4.7183	237.8634
B1control	B	control	0	0	1.2014	0.142	5.943	1.7953	4.3559	4.9151	236.8934
B1control	B	control	0	0	0.9954	0.1176	5.485	1.7989	5.1197	4.6877	236.8976
B1control	B	control	0	0	1.1939	0.1411	6.085	2.0692	6.054	4.8846	237.2181
B1control	B	control	0	0	1.1939	0.1411	5.343	2.0804	4.5927	4.7047	220.3076
B1control	B	control	0	0	1.2037	0.1453	5.732	2.0621	4.5978	4.9016	246.3967
B1Cu1/2	B	Cu	A	0.13	0.0095	0.0015	19.335	1.9316	5.0649	4.3147	195.2358
B1Cu1/2	B	Cu	A	0.13	0.0103	0.0016	19.818	1.9341	5.0923	3.9288	195.2965
B1Cu1/2	B	Cu	A	0.13	0.015	0.0023	18.852	1.9356	5.0923	4.338	186.6018
B1Cu1/2	B	Cu	A	0.13	0.0116	0.0018	19.342	1.8438	4.9925	3.9022	201.0522
B1Cu1/2	B	Cu	A	0.13	0.0116	0.0018	19.328	1.8503	5.01	4.2914	173.2842
B1Cu1/2	B	Cu	A	0.13	0.0118	0.0018	18.857	1.8458	4.9975	3.8822	212.5543
B1Cu0	B	Cu	B	0.26	0.0012	0.0002	34.481	2.1487	6.1751	2.7379	183.6135
B1Cu0	B	Cu	B	0.26	0.008	0.0012	39.574	1.8922	3.7375	2.5649	200.793
B1Cu0	B	Cu	B	0.26	0.0014	0.0002	29.387	2.2156	3.7001	2.7079	201.1366
B1Cu0	B	Cu	B	0.26	0.0035	0.0005	34.573	2.2315	6.3024	2.5349	195.181
B1Cu0	B	Cu	B	0.26	0.0035	0.0005	34.388	2.1891	3.7799	2.7246	183.0839
B1Cu0	B	Cu	B	0.26	0.0036	0.0005	29.448	1.8887	3.8523	2.5516	216.1785
B1Cu2.5	B	Cu	C	0.65	0.0008	0.0001	68.003	2.0781	8.7302	5.6682	148.8108
B1Cu2.5	B	Cu	C	0.65	0.0005	0.0001	78.434	2.1219	8.786	5.2248	149.055
B1Cu2.5	B	Cu	C	0.65	0.0002	0.0001	57.573	2.3116	8.9814	5.6403	142.6337
B1Cu2.5	B	Cu	C	0.65	0.0005	0.0001	71.933	2.3237	8.5558	5.2527	146.8331
B1Cu2.5	B	Cu	C	0.65	0.0005	0.0001	64.073	2.0558	8.5651	5.6558	145.0304
B1Cu2.5	B	Cu	C	0.65	0.0005	0.0001	60.193	2.2679	9.186	5.2403	158.8319
B1Cu5	B	Cu	D	1.3	0.0005	0.0001	296.38	4.9851	7.9833	5.0558	77.5395
B1Cu5	B	Cu	D	1.3	0.001	0.0002	322.204	5.334	8.0266	4.5353	77.7492
B1Cu5	B	Cu	D	1.3	0.0003	0.0001	270.557	5.3271	7.9926	5.0186	74.3559
B1Cu5	B	Cu	D	1.3	0.0006	0.0001	323.054	5.1264	7.723	4.4981	76.5482

B1Cu5	B	Cu	D	1.3	0.0006	0.0001	269.706	5.3309	7.7416	5.0392	72.6601
B1Cu5	B	Cu	D	1.3	0.0006	0.0001	252.774	5.3519	7.7292	4.5188	81.7155
B1control	B	control	0	0	1.3849	0.1637	4.008	1.7918	5.1222	4.7183	237.8634
B1control	B	control	0	0	1.2014	0.142	4.035	1.7953	4.3559	4.9151	236.8934
B1control	B	control	0	0	0.9954	0.1176	3.981	1.7989	5.1197	4.6877	236.8976
B1control	B	control	0	0	1.1939	0.1411	4.191	2.0692	6.054	4.8846	237.2181
B1control	B	control	0	0	1.1939	0.1411	3.825	2.0804	4.5927	4.7047	220.3076
B1control	B	control	0	0	1.2037	0.1453	3.859	2.0621	4.5978	4.9016	246.3967
B1Pb0	B	Pb	A	0.025	0.1143	0.0123	4.1	4.4144	2.5287	3.2122	288.5747
B1Pb0	B	Pb	A	0.025	0.4052	0.0438	4.793	4.1587	2.5598	3.2792	294.6834
B1Pb0	B	Pb	A	0.025	0.2732	0.0295	3.407	4.0148	2.512	3.2409	295.2696
B1Pb0	B	Pb	A	0.025	0.2642	0.0285	4.223	4.4551	2.5956	3.3078	292.8426
B1Pb0	B	Pb	A	0.025	0.2642	0.0285	3.977	4.2213	2.6267	3.2282	282.0204
B1Pb0	B	Pb	A	0.025	0.2701	0.0298	3.325	4.0148	2.5789	3.2951	312.6737
B1Pb10	B	Pb	B	0.25	0.0576	0.0074	351	4.6583	3.5545	4.2246	247.1796
B1Pb10	B	Pb	B	0.25	0.0346	0.0045	389.105	4.9899	3.5859	4.1023	231.9865
B1Pb10	B	Pb	B	0.25	0.0218	0.0028	312.895	4.8195	3.5376	4.2535	232.128
B1Pb10	B	Pb	B	0.25	0.038	0.0049	365.509	4.6081	3.4653	4.1313	237.098
B1Pb10	B	Pb	B	0.25	0.038	0.0049	336.491	4.847	3.4966	4.2407	223.7672
B1Pb10	B	Pb	B	0.25	0.0383	0.0049	303.222	4.8388	3.4484	4.1184	250.3257
B1Pb50	B	Pb	C	1.25	0.0113	0.0022	459	5.0406	7.9896	10.4385	131.7322
B1Pb50	B	Pb	C	1.25	0.0099	0.0019	538.674	5.0762	8.0218	9.4527	131.7085
B1Pb50	B	Pb	C	1.25	0.017	0.0033	379.326	5.7043	7.9723	10.4682	145.6403
B1Pb50	B	Pb	C	1.25	0.0127	0.0024	473.319	5.5653	8.2765	9.423	136.3603
B1Pb50	B	Pb	C	1.25	0.0127	0.0025	444.681	5.0213	8.3086	10.455	134.7687
B1Pb50	B	Pb	C	1.25	0.0131	0.0025	369.779	5.0406	8.2591	9.4395	148.5261
B1Pb100	B	Pb	D	2.5	0.0312	0.0062	1082	6.8263	10.5986	12.5021	100.8734
B1Pb100	B	Pb	D	2.5	0.0105	0.0021	1194.583	6.9237	10.639	12.5931	101.1838
B1Pb100	B	Pb	D	2.5	0.0087	0.0017	969.417	6.5993	10.5769	12.4648	103.5347
B1Pb100	B	Pb	D	2.5	0.0168	0.0033	1089.762	6.5502	11.3151	12.6303	101.864
B1Pb100	B	Pb	D	2.5	0.0168	0.0033	1074.238	6.8182	11.3555	12.4855	99.3746

B1Pb100	B	Pb	D	2.5	0.0176	0.0034	964.242	6.9888	11.2934	12.6137	109.0668
B1control	B	control	0	0	1.3849	0.1637	0.237	1.7918	5.1222	4.7183	237.8634
B1control	B	control	0	0	1.2014	0.142	0.257	1.7953	4.3559	4.9151	236.8934
B1control	B	control	0	0	0.9954	0.1176	0.283	1.7989	5.1197	4.6877	236.8976
B1control	B	control	0	0	1.1939	0.1411	0.238	2.0692	6.054	4.8846	237.2181
B1control	B	control	0	0	1.1939	0.1411	0.203	2.0804	4.5927	4.7047	220.3076
B1control	B	control	0	0	1.2037	0.1453	0.272	2.0621	4.5978	4.9016	246.3967
B1Cr0	B	Cr	A	0.036	0.3356	0.0408	1.624	2.2612	4.9761	4.6954	278.1504
B1Cr0	B	Cr	A	0.036	0.5116	0.0622	1.684	2.1962	5.1746	4.1818	278.085
B1Cr0	B	Cr	A	0.036	0.5303	0.0645	1.564	2.09	5.0813	4.6667	288.2379
B1Cr0	B	Cr	A	0.036	0.4592	0.0559	1.883	2.0866	5.1364	4.1531	281.4911
B1Cr0	B	Cr	A	0.036	0.4592	0.0559	1.365	2.2488	4.9211	4.6826	260.6558
B1Cr0	B	Cr	A	0.036	0.4803	0.058	1.391	2.1962	5.1483	4.1691	282.9568
B1Cr5	B	Cr	B	0.18	0.015	0.0019	2.051	1.8926	7.3787	4.8812	249.7377
B1Cr5	B	Cr	B	0.18	0.0492	0.0062	2.651	1.902	7.3119	4.4554	248.7837
B1Cr5	B	Cr	B	0.18	0.0665	0.0083	2.451	1.8876	7.0644	4.8515	249.1269
B1Cr5	B	Cr	B	0.18	0.0436	0.0055	2.18	1.9881	7.1683	4.4257	249.2161
B1Cr5	B	Cr	B	0.18	0.0436	0.0055	1.922	1.9851	7.0743	4.868	240.1475
B1Cr5	B	Cr	B	0.18	0.0445	0.0055	2.365	1.9812	7.2228	4.4422	257.7214
B1Cr10	B	Cr	C	0.36	0.0381	0.0062	21.269	2.5454	11.328	5.419	228.6754
B1Cr10	B	Cr	C	0.36	0.01	0.0016	25.94	2.5747	11.224	5.4033	228.7959
B1Cr10	B	Cr	C	0.36	0.0112	0.0018	20.598	2.4414	10.3828	5.4474	216.4367
B1Cr10	B	Cr	C	0.36	0.0198	0.0032	21.968	2.466	11.5595	5.4316	224.636
B1Cr10	B	Cr	C	0.36	0.0198	0.0032	20.57	2.5477	11.5643	5.4348	202.1786
B1Cr10	B	Cr	C	0.36	0.0206	0.0032	16.132	2.5931	10.6191	5.419	240.4337
B1Cr25	B	Cr	D	0.90	0.06	0.0142	20.469	3.8986	12.7023	9.5577	205.8413
B1Cr25	B	Cr	D	0.90	0.0452	0.0107	23.94	4.082	12.6294	8.9608	206.2528
B1Cr25	B	Cr	D	0.90	0.0238	0.0056	19.478	4.2443	11.9471	9.5254	206.5818
B1Cr25	B	Cr	D	0.90	0.043	0.0102	21.968	4.2163	11.9795	8.9932	206.2253
B1Cr25	B	Cr	D	0.90	0.043	0.0102	20.57	3.9056	12.7157	9.5433	205.1626
B1Cr25	B	Cr	D	0.90	0.0434	0.0105	18.2132	4.103	12.5539	8.9788	221.7355

B1control	B	control	0	0	1.3849	0.1637	5.698	1.7918	5.1222	4.7183	237.8634
B1control	B	control	0	0	1.2014	0.142	5.902	1.7953	4.3559	4.9151	236.8934
B1control	B	control	0	0	0.9954	0.1176	5.494	1.7989	5.1197	4.6877	236.8976
B1control	B	control	0	0	1.1939	0.1411	5.905	2.0692	6.054	4.8846	237.2181
B1control	B	control	0	0	1.1939	0.1411	5.49	2.0804	4.5927	4.7047	220.3076
B1control	B	control	0	0	1.2037	0.1453	5.355	2.0621	4.5978	4.9016	246.3967
B1Ni0	B	Ni	A	0.025	0.1609	0.0202	8.575	3.2139	3.1029	5.7316	280.2845
B1Ni0	B	Ni	A	0.025	0.1235	0.0155	8.819	3.0096	3.0474	5.5735	275.905
B1Ni0	B	Ni	A	0.025	0.2009	0.0253	8.331	3.1776	3.055	5.7619	275.4205
B1Ni0	B	Ni	A	0.025	0.1618	0.0204	8.645	3.1645	3.1609	5.6038	277.2033
B1Ni0	B	Ni	A	0.025	0.1618	0.0204	8.505	3.2371	3.499	5.7484	255.6139
B1Ni0	B	Ni	A	0.025	0.1693	0.0204	8.284	2.9995	3.4485	5.5903	291.577
B1Ni5	B	Ni	B	0.125	0.0178	0.0023	35.644	4.61	4.9272	4.9843	205.1654
B1Ni5	B	Ni	B	0.125	0.0072	0.0009	42.393	5.2646	5.2138	5.0909	203.164
B1Ni5	B	Ni	B	0.125	0.0056	0.0007	28.895	4.9196	5.1927	5.0125	212.799
B1Ni5	B	Ni	B	0.125	0.0102	0.0013	35.91	4.6259	3.696	5.119	207.0428
B1Ni5	B	Ni	B	0.125	0.0102	0.0013	35.378	5.0508	4.0508	5	192.3703
B1Ni5	B	Ni	B	0.125	0.0104	0.0013	28.718	4.8557	4.0367	5.1065	218.442
B1Ni10	B	Ni	C	0.25	0.0242	0.0044	44.355	5.0025	4.703	5.7558	171.0709
B1Ni10	B	Ni	C	0.25	0.0065	0.0012	62.3	5.0559	5.8886	6.0264	174.5227
B1Ni10	B	Ni	C	0.25	0.0125	0.0023	56.41	5.4228	5.8936	5.7261	174.2241
B1Ni10	B	Ni	C	0.25	0.0144	0.0026	45.637	5.0307	4.7475	5.9967	173.2726
B1Ni10	B	Ni	C	0.25	0.0144	0.0026	47.073	4.9688	5.8267	5.7426	160.2751
B1Ni10	B	Ni	C	0.25	0.0144	0.0027	55.556	5.3277	5.8193	6.0132	183.4967
B1Ni25	B	Ni	D	0.625	0.0032	0.0007	404.345	6.7044	10.011	9.8059	108.0764
B1Ni25	B	Ni	D	0.625	0.0024	0.0005	503.64	6.0747	10.0659	9.8315	107.6976
B1Ni25	B	Ni	D	0.625	0.0014	0.0003	365.051	6.1165	10.0577	9.7729	113.1046
B1Ni25	B	Ni	D	0.625	0.0023	0.0005	406.591	6.028	10.011	9.7985	109.6262
B1Ni25	B	Ni	D	0.625	0.0023	0.0005	402.1	6.1242	10	9.7912	105.5434
B1Ni25	B	Ni	D	0.625	0.0023	0.0005	383.554	6.1242	10.0522	9.8168	114.4584
B1control	B	control	0	0	1.3849	0.1637	0.455	1.7918	5.1222	4.7183	237.8634

B1control	B	control	0	0	1.2014	0.142	0.485	1.7953	4.3559	4.9151	236.8934
B1control	B	control	0	0	0.9954	0.1176	0.425	1.7989	5.1197	4.6877	236.8976
B1control	B	control	0	0	1.1939	0.1411	0.536	2.0692	6.054	4.8846	237.2181
B1control	B	control	0	0	1.1939	0.1411	0.374	2.0804	4.5927	4.7047	220.3076
B1control	B	control	0	0	1.2037	0.1453	0.371	2.0621	4.5978	4.9016	246.3967
B1Cd0	B	Cd	A	0.0000945	0.0261	0.0028	0.621	4.666	4.4766	4.9186	269.0429
B1Cd0	B	Cd	A	0.0000945	0.3214	0.035	0.638	5.015	4.499	4.9518	262.8842
B1Cd0	B	Cd	A	0.0000945	0.6755	0.0735	0.604	5.0543	4.4666	4.8887	262.9326
B1Cd0	B	Cd	A	0.0000945	0.341	0.0371	0.696	4.7767	4.6211	4.9219	264.9532
B1Cd0	B	Cd	A	0.0000945	0.341	0.0371	0.546	5.0264	4.6436	4.9053	264.2366
B1Cd0	B	Cd	A	0.0000945	0.3509	0.0386	0.554	5.1142	4.6112	4.9385	264.2956
B1Cd100	B	Cd	B	0.00945	0.1568	0.0156	2.925	4.825	4.3025	4.6724	263.1326
B1Cd100	B	Cd	B	0.00945	0.0796	0.0079	2.239	4.8428	4.3241	4.813	251.4612
B1Cd100	B	Cd	B	0.00945	0.1868	0.0185	2.611	5.0326	4.2929	4.6437	251.3725
B1Cd100	B	Cd	B	0.00945	0.1411	0.014	3.072	4.8389	4.9473	4.8418	255.3221
B1Cd100	B	Cd	B	0.00945	0.1411	0.014	2.779	5.4199	4.9688	4.6596	230.9408
B1Cd100	B	Cd	B	0.00945	0.1434	0.0143	2.514	5.2752	4.9377	4.829	264.4569
B1Cd500	B	Cd	C	0.04725	0.0234	0.0037	16.131	5.3432	5.0922	5.6592	259.9263
B1Cd500	B	Cd	C	0.04725	0.0157	0.0025	17.323	5.3489	5.1153	5.5225	248.1381
B1Cd500	B	Cd	C	0.04725	0.0226	0.0036	17.939	5.3535	5.082	5.6899	248.3904
B1Cd500	B	Cd	C	0.04725	0.0206	0.0033	16.384	5.3607	5.1691	5.5533	252.1516
B1Cd500	B	Cd	C	0.04725	0.0206	0.0033	18.878	5.3458	5.1921	5.6762	238.3866
B1Cd500	B	Cd	C	0.04725	0.0208	0.0034	17.107	5.3145	5.1588	5.5396	252.6669
B1Cd1000	B	Cd	D	0.0945	0.0151	0.003	34.846	4.884	6.1229	3.7019	254.5782
B1Cd1000	B	Cd	D	0.0095	0.0359	0.007	38.014	4.8727	6.1451	3.6788	233.3572
B1Cd1000	B	Cd	D	0.0945	0.031	0.0061	31.678	4.7517	6.113	3.6723	233.0401
B1Cd1000	B	Cd	D	0.0945	0.0273	0.0054	34.887	4.8001	6.4931	3.7085	240.3252
B1Cd1000	B	Cd	D	0.0945	0.0273	0.0054	34.804	4.8672	6.5153	3.6887	235.1475
B1Cd1000	B	Cd	D	0.0945	0.0285	0.0056	32.706	4.7157	6.4832	3.6953	243.166
B1control	B	control	0	0	1.3849	0.1637	29.391	1.7918	5.1222	4.7183	237.8634
B1control	B	control	0	0	1.2014	0.142	31.014	1.7953	4.3559	4.9151	236.8934

B1control	B	control	0	0	0.9954	0.1176	27.768	1.7989	5.1197	4.6877	236.8976
B1control	B	control	0	0	1.1939	0.1411	30.74	2.0692	6.054	4.8846	237.2181
B1control	B	control	0	0	1.1939	0.1411	28.043	2.0804	4.5927	4.7047	220.3076
B1control	B	control	0	0	1.2037	0.1453	28.668	2.0621	4.5978	4.9016	246.3967
B1Zn0	B	Zn	A	0.28	0.0834	0.0102	468.568	1.3226	5.1152	4.4614	309.6737
B1Zn0	B	Zn	A	0.28	0.2026	0.0248	513.498	1.8998	5.1378	4.4264	293.7883
B1Zn0	B	Zn	A	0.28	0.191	0.0234	423.638	1.7715	5.1052	4.3562	293.9301
B1Zn0	B	Zn	A	0.28	0.159	0.0195	479.523	1.7685	4.9875	4.7846	299.1307
B1Zn0	B	Zn	A	0.28	0.159	0.0195	457.613	1.3422	5.01	4.6543	275.7369
B1Zn0	B	Zn	A	0.28	0.1615	0.0197	430.941	1.9259	4.9775	4.5591	298.5154
B1Zn2	B	Zn	B	0.56	0.1022	0.0165	750.268	2.6728	4.7203	6.357	189.8411
B1Zn2	B	Zn	B	0.56	0.0868	0.014	841.723	2.6803	4.7428	6.367	189.6192
B1Zn2	B	Zn	B	0.56	0.0974	0.0157	658.813	2.6983	4.7103	6.3869	192.8111
B1Zn2	B	Zn	B	0.56	0.0955	0.0154	769.821	2.6364	4.4306	6.337	190.7571
B1Zn2	B	Zn	B	0.56	0.0955	0.0154	730.715	2.6274	4.453	6.3736	183.5996
B1Zn2	B	Zn	B	0.56	0.0988	0.0161	671.849	2.6239	4.4206	6.3536	204.0568
B1Zn4	B	Zn	C	1.12	0.0414	0.0118	738.775	2.55	6.836	7.1209	153.3628
B1Zn4	B	Zn	C	1.12	0.0393	0.0112	833.061	2.2874	6.8583	6.3751	153.4766
B1Zn4	B	Zn	C	1.12	0.0501	0.0142	644.49	2.6541	6.8261	6.8038	156.6109
B1Zn4	B	Zn	C	1.12	0.0436	0.0124	805.276	2.6283	6.3751	6.8038	154.4834
B1Zn4	B	Zn	C	1.12	0.0436	0.0124	672.275	2.56	6.3974	6.7344	146.9677
B1Zn4	B	Zn	C	1.12	0.0437	0.0124	688.823	2.3167	6.3652	7.0243	166.578
B1Zn10	B	Zn	D	2.8	0.025	0.0069	2089.521	5.8738	14.9907	8.2383	88.1468
B1Zn10	B	Zn	D	2.8	0.0166	0.0045	2301.819	5.7766	15.3084	8.0654	88.0608
B1Zn10	B	Zn	D	2.8	0.0063	0.0017	1877.223	5.8682	14.285	7.9907	89.472
B1Zn10	B	Zn	D	2.8	0.016	0.0044	2142.942	5.8804	14.2056	8.5467	88.5599
B1Zn10	B	Zn	D	2.8	0.016	0.0044	2036.1	5.8617	16.215	8.4439	87.4512
B1Zn10	B	Zn	D	2.8	0.016	0.0045	1912.837	5.7215	15.1168	8.1495	89.7478
M1control	M	control	0	0	1.3754	0.1659	7.096	1.7307	4.9226	4.9936	188.2598
M1control	M	control	0	0	1.2034	0.1451	7.43	1.7316	5.1886	4.5164	190.2882
M1control	M	control	0	0	0.9924	0.1197	6.762	1.7253	5.2031	5.0226	191.4813

M1control	M	control	0	0	1.19	0.1435	7.461	1.7645	4.913	4.4874	190.0098
M1control	M	control	0	0	1.1903	0.1436	6.731	1.7698	5.1886	5.0097	189.779
M1control	M	control	0	0	1.2473	0.1473	6.519	1.7664	5.3095	4.5035	205.8039
M1Cu1/2	M	Cu	A	0.13	0.0146	0.0025	22.102	2.6711	3.8412	4.458	189.6449
M1Cu1/2	M	Cu	A	0.13	0.0184	0.0032	22.435	2.6739	3.844	4.505	197.3402
M1Cu1/2	M	Cu	A	0.13	0.0334	0.0058	21.769	2.5977	3.8534	4.4831	192.0883
M1Cu1/2	M	Cu	A	0.13	0.0217	0.0038	22.84	2.6358	3.7688	4.5207	184.0387
M1Cu1/2	M	Cu	A	0.13	0.022	0.0038	21.363	2.618	3.7716	4.4518	181.1289
M1Cu1/2	M	Cu	A	0.13	0.0229	0.0039	21.277	2.5446	3.7735	4.4831	199.4947
M1Cu0	M	Cu	B	0.26	0.0013	0.0002	26.042	3.9771	4.6368	4.1976	244.1126
M1Cu0	M	Cu	B	0.26	0.0022	0.0003	31.358	3.816	4.1659	3.4509	238.5687
M1Cu0	M	Cu	B	0.26	0.0082	0.001	20.725	3.9762	4.5386	4.2262	240.1617
M1Cu0	M	Cu	B	0.26	0.0037	0.0004	28.623	4.0033	4.5834	3.4795	240.9477
M1Cu0	M	Cu	B	0.26	0.0039	0.0005	23.46	3.8189	4.1897	4.2135	238.0166
M1Cu0	M	Cu	B	0.26	0.004	0.0005	29.004	3.7969	4.6873	3.4668	259.7258
M1Cu2.5	M	Cu	C	0.65	0.003	0.0008	34.317	4.2886	10.5959	7.171	182.0104
M1Cu2.5	M	Cu	C	0.65	0.009	0.0023	36.233	4.3689	10.842	6.7737	182.2707
M1Cu2.5	M	Cu	C	0.65	0.0021	0.0005	32.401	4.2984	10.6244	7.1399	188.9054
M1Cu2.5	M	Cu	C	0.65	0.0047	0.0012	35.555	4.2637	10.5959	6.8048	184.3955
M1Cu2.5	M	Cu	C	0.65	0.0047	0.0012	33.078	4.2819	10.5518	7.1572	167.2327
M1Cu2.5	M	Cu	C	0.65	0.0049	0.0012	31.575	4.3756	10.886	6.791	194.3557
M1Cu5	M	Cu	D	1.3	0.0111	0.0029	189.009	4.4656	14.907	7.5763	56.5494
M1Cu5	M	Cu	D	1.3	0.0021	0.0005	202.544	4.4723	14.9427	7.5	59.6936
M1Cu5	M	Cu	D	1.3	0.0021	0.0005	175.475	4.4728	14.9117	7.57	61.9082
M1Cu5	M	Cu	D	1.3	0.005	0.0013	204.71	4.4928	14.9642	7.4841	59.7583
M1Cu5	M	Cu	D	1.3	0.0051	0.0013	173.309	4.4967	14.9928	7.5477	62.9372
M1Cu5	M	Cu	D	1.3	0.0052	0.0013	165.008	4.499	14.9761	7.465	64.8448
M1control	M	control	0	0	1.3754	0.1659	0.359	1.7307	4.9226	4.9936	188.2598
M1control	M	control	0	0	1.2034	0.1451	0.319	1.7316	5.1886	4.5164	190.2882
M1control	M	control	0	0	0.9924	0.1197	0.399	1.7253	5.2031	5.0226	191.4813
M1control	M	control	0	0	1.19	0.1435	0.353	1.7645	4.913	4.4874	190.0098

M1control	M	control	0	0	1.1903	0.1436	0.264	1.7698	5.1886	5.0097	189.779
M1control	M	control	0	0	1.2473	0.1473	0.302	1.7664	5.3095	4.5035	205.8039
M1Pb0	M	Pb	A	0.025	0.1094	0.0159	1.9	2.7411	5.2978	6.5738	253.5818
M1Pb0	M	Pb	A	0.025	0.3644	0.0529	2.246	2.7671	5.8799	6.3144	253.8755
M1Pb0	M	Pb	A	0.025	0.2704	0.0392	1.554	2.7598	5.2911	6.545	251.0684
M1Pb0	M	Pb	A	0.025	0.2477	0.0359	1.929	2.6825	5.2373	6.2856	252.8419
M1Pb0	M	Pb	A	0.025	0.248	0.036	1.871	2.6796	5.8021	6.561	234.1111
M1Pb0	M	Pb	A	0.025	0.2579	0.036	1.534	2.6787	5.2949	6.3016	268.6775
M1Pb10	M	Pb	B	0.25	0.0782	0.0097	169.27	2.8443	6.3639	6.0427	204.3807
M1Pb10	M	Pb	B	0.25	0.0432	0.0054	169.968	2.8478	6.3839	6.4097	202.4256
M1Pb10	M	Pb	B	0.25	0.0192	0.0024	168.572	2.7703	6.4339	6.0127	204.8838
M1Pb10	M	Pb	B	0.25	0.0467	0.0058	182.574	2.8073	6.3488	6.3797	203.8967
M1Pb10	M	Pb	B	0.25	0.0468	0.0058	155.966	2.7863	6.4339	6.0294	203.0936
M1Pb10	M	Pb	B	0.25	0.0487	0.0061	159.703	2.7102	6.4765	6.3964	223.9393
M1Pb50	M	Pb	C	1.25	0.0052	0.001	392	5.1253	11.6189	9.1897	199.5415
M1Pb50	M	Pb	C	1.25	0.0112	0.0022	443.962	4.7707	11.5207	8.9455	199.4484
M1Pb50	M	Pb	C	1.25	0.0152	0.003	340.038	4.9862	12.1576	9.1578	198.7988
M1Pb50	M	Pb	C	1.25	0.0103	0.0021	421.487	4.9061	11.6481	8.9137	199.2629
M1Pb50	M	Pb	C	1.25	0.0105	0.0021	362.513	5.1354	11.8551	9.1755	194.5797
M1Pb50	M	Pb	C	1.25	0.011	0.0021	320.38	4.8195	11.8816	8.9314	200.8985
M1Pb100	M	Pb	D	2.5	0.0214	0.0044	1582	8.4229	13.9347	12.3804	89.864
M1Pb100	M	Pb	D	2.5	0.0164	0.0034	1789.846	8.4529	14.0632	12.5303	90.1246
M1Pb100	M	Pb	D	2.5	0.0094	0.0019	1374.154	8.8694	13.94	12.4126	90.3182
M1Pb100	M	Pb	D	2.5	0.0153	0.0032	1596.641	8.8833	14.0525	12.4982	90.1022
M1Pb100	M	Pb	D	2.5	0.0156	0.0032	1567.359	8.4047	15.0161	12.7552	85.0197
M1Pb100	M	Pb	D	2.5	0.0162	0.0032	1364.393	8.4186	14.2398	12.5161	94.8756
M1control	M	control	0	0	1.3754	0.1659	2.213	1.7307	4.9226	4.9936	188.2598
M1control	M	control	0	0	1.2034	0.1451	2.289	1.7316	5.1886	4.5164	190.2882
M1control	M	control	0	0	0.9924	0.1197	2.137	1.7253	5.2031	5.0226	191.4813
M1control	M	control	0	0	1.19	0.1435	2.428	1.7645	4.913	4.4874	190.0098
M1control	M	control	0	0	1.1903	0.1436	1.998	1.7698	5.1886	5.0097	189.779

M1control	M	control	0	0	1.2473	0.1473	1.993	1.7664	5.3095	4.5035	205.8039
M1Cr0	M	Cr	A	0.036	0.3263	0.039	0.427	2.2581	6.4926	3.9934	218.4653
M1Cr0	M	Cr	A	0.036	0.7513	0.0898	0.4179	2.2951	6.7167	3.7274	218.3999
M1Cr0	M	Cr	A	0.036	0.5273	0.063	0.37	2.0128	6.1946	4.023	225.815
M1Cr0	M	Cr	A	0.036	0.5347	0.0639	0.361	2.6704	6.6207	3.757	220.8934
M1Cr0	M	Cr	A	0.036	0.5349	0.0639	0.393	2.2773	6.2635	4.0099	205.5525
M1Cr0	M	Cr	A	0.036	0.56	0.0648	0.314	2.2956	6.5345	3.7438	228.78
M1Cr5	M	Cr	B	0.18	0.0632	0.0098	3.14	1.8608	5.5496	4.7547	211.0797
M1Cr5	M	Cr	B	0.18	0.0512	0.0079	3.193	1.8608	5.6893	4.6986	210.9825
M1Cr5	M	Cr	B	0.18	0.0642	0.01	3.087	1.8608	5.9707	4.7813	211.8188
M1Cr5	M	Cr	B	0.18	0.0593	0.0092	3.36	1.9282	5.6671	4.672	211.2936
M1Cr5	M	Cr	B	0.18	0.0595	0.0092	2.92	1.9282	5.5629	4.7695	191.4051
M1Cr5	M	Cr	B	0.18	0.0617	0.0094	2.941	1.9282	5.809	4.6868	217.1864
M1Cr10	M	Cr	C	0.36	0.0312	0.0039	4.438	1.9196	5.9925	5.1223	218.0863
M1Cr10	M	Cr	C	0.36	0.0122	0.0015	4.5	1.7267	5.8161	5.1529	206.4285
M1Cr10	M	Cr	C	0.36	0.0072	0.0009	4.376	1.73	6.096	5.1388	217.3153
M1Cr10	M	Cr	C	0.36	0.0167	0.0021	4.712	1.7056	5.8702	5.334	213.9434
M1Cr10	M	Cr	C	0.36	0.0168	0.0021	4.164	1.9276	6.0536	5.3857	204.59
M1Cr10	M	Cr	C	0.36	0.0174	0.0022	4.193	1.9309	5.9948	5.1717	220.4544
M1Cr25	M	Cr	D	0.90	0.0573	0.015	19.345	3.8989	14.4513	10.0318	224.6823
M1Cr25	M	Cr	D	0.90	0.0463	0.0121	21.84	3.9485	13.5448	9.9427	224.5754
M1Cr25	M	Cr	D	0.90	0.0683	0.0178	14.85	4.1126	14.2366	10.0032	226.0176
M1Cr25	M	Cr	D	0.90	0.057	0.0149	21.68	4.1479	14.6756	9.9141	225.0918
M1Cr25	M	Cr	D	0.90	0.0572	0.015	17.011	3.7199	12.2137	10.0191	224.7524
M1Cr25	M	Cr	D	0.90	0.0578	0.0155	18.294	3.7638	13.812	9.93	225.2631
M1control	M	control	0	0	1.3754	0.1659	15.466	1.7307	4.9226	4.9936	188.2598
M1control	M	control	0	0	1.2034	0.1451	15.934	1.7316	5.1886	4.5164	190.2882
M1control	M	control	0	0	0.9924	0.1197	14.998	1.7253	5.2031	5.0226	191.4813
M1control	M	control	0	0	1.19	0.1435	18.253	1.7645	4.913	4.4874	190.0098
M1control	M	control	0	0	1.1903	0.1436	12.679	1.7698	5.1886	5.0097	189.779
M1control	M	control	0	0	1.2473	0.1473	13.14	1.7664	5.3095	4.5035	205.8039

M1Ni0	M	Ni	A	0.025	0.1513	0.0195	24.255	1.9669	2.8595	4.7824	226.3754
M1Ni0	M	Ni	A	0.025	0.2373	0.0306	30.768	1.7318	3.2072	4.9338	226.6559
M1Ni0	M	Ni	A	0.025	0.1923	0.0248	23.743	1.6358	3.2214	4.9976	236.6469
M1Ni0	M	Ni	A	0.025	0.1933	0.0249	25.3	1.9943	3.2308	5.0828	229.8928
M1Ni0	M	Ni	A	0.025	0.1936	0.025	23.211	1.5199	3.3373	5.1537	227.2006
M1Ni0	M	Ni	A	0.025	0.2007	0.0255	21.047	1.8269	3.3136	4.8463	249.9366
M1Ni5	M	Ni	B	0.125	0.0034	0.0003	38.081	2.398	4.2512	6.5616	226.0671
M1Ni5	M	Ni	B	0.125	0.0141	0.0014	44.703	2.4346	3.5387	6.1557	221.0062
M1Ni5	M	Ni	B	0.125	0.0017	0.0002	31.46	2.4021	4.0755	6.022	223.3107
M1Ni5	M	Ni	B	0.125	0.0063	0.0006	39.012	2.4386	4.2139	6.022	223.4614
M1Ni5	M	Ni	B	0.125	0.0064	0.0006	37.15	2.4659	3.5492	6.8625	214.2401
M1Ni5	M	Ni	B	0.125	0.0064	0.0006	32.08	2.453	4.1882	6.5473	224.2695
M1Ni10	M	Ni	C	0.25	0.009	0.0013	68.681	4.1371	7.1711	8.0769	203.6387
M1Ni10	M	Ni	C	0.25	0.012	0.0018	85.446	4.1356	7.1103	8.087	194.5838
M1Ni10	M	Ni	C	0.25	0.01	0.0015	61.916	4.1311	7.1179	7.915	197.5565
M1Ni10	M	Ni	C	0.25	0.0103	0.0015	73.356	4.1569	7.6139	7.9757	198.593
M1Ni10	M	Ni	C	0.25	0.0103	0.0015	64.006	4.1594	8.8537	7.1559	179.6189
M1Ni10	M	Ni	C	0.25	0.0105	0.0016	65.033	4.1508	8.8411	7.247	213.2755
M1Ni25	M	Ni	D	0.625	0.0103	0.0018	320.7275	5.6424	8.2097	11.3963	105.4891
M1Ni25	M	Ni	D	0.625	0.0033	0.0006	383.5462	5.2336	8.1475	11.2535	106.0036
M1Ni25	M	Ni	D	0.625	0.0008	0.0001	257.9088	5.6452	8.1336	11.2811	110.784
M1Ni25	M	Ni	D	0.625	0.0045	0.0008	326.2447	4.6848	8.3802	11.553	107.4256
M1Ni25	M	Ni	D	0.625	0.0047	0.0008	315.2103	5.2797	9.8525	11.7189	96.8428
M1Ni25	M	Ni	D	0.625	0.0047	0.0009	261.5869	5.6382	9.8664	11.9217	112.2711
M1control	M	control	0	0	1.3754	0.1659	0.684	1.7307	1.9226	4.9936	188.2598
M1control	M	control	0	0	1.2034	0.1451	0.508	1.7316	2.1886	4.5164	190.2882
M1control	M	control	0	0	0.9924	0.1197	0.676	1.7253	2.2031	5.0226	191.4813
M1control	M	control	0	0	1.19	0.1435	0.624	1.7645	1.913	4.4874	190.0098
M1control	M	control	0	0	1.1903	0.1436	0.543	1.7698	2.1886	5.0097	189.779
M1control	M	control	0	0	1.2473	0.1473	0.492	1.7664	2.3095	4.5035	205.8039
M1Cd0	M	Cd	A	0.0000945	0.0163	0.0018	0.732	2.3716	2.0185	2.5678	203.1726

M1Cd0	M	Cd	A	0.0000945	0.3543	0.039	0.84	2.495	1.9959	2.7939	207.1028
M1Cd0	M	Cd	A	0.0000945	0.6743	0.0742	0.624	2.54	1.9869	2.5407	208.1347
M1Cd0	M	Cd	A	0.0000945	0.348	0.0383	0.827	2.4193	2.4571	2.7667	206.1367
M1Cd0	M	Cd	A	0.0000945	0.3482	0.0383	0.637	2.5016	2.4684	2.5558	204.5327
M1Cd0	M	Cd	A	0.0000945	0.3583	0.0384	0.688	2.5389	2.4412	2.7818	221.6421
M1Cd100	M	Cd	B	0.00945	0.0564	0.0055	73.789	1.3198	2.905	2.1446	216.2476
M1Cd100	M	Cd	B	0.00945	0.0824	0.0081	74.073	1.5226	2.9818	2.1601	216.0658
M1Cd100	M	Cd	B	0.00945	0.1794	0.0176	73.505	1.3822	2.898	2.1167	215.1713
M1Cd100	M	Cd	B	0.00945	0.1057	0.0104	74.784	1.4753	3.0214	2.1322	215.8282
M1Cd100	M	Cd	B	0.00945	0.106	0.0104	72.793	1.3152	2.9679	2.1322	206.4991
M1Cd100	M	Cd	B	0.00945	0.1085	0.0105	74.168	1.4921	3.0214	2.1477	229.3376
M1Cd500	M	Cd	C	0.04725	0.0151	0.0017	118.624	1.5014	2.7236	2.8141	160.273
M1Cd500	M	Cd	C	0.04725	0.0161	0.0019	137.934	1.7106	2.851	2.8333	160.1642
M1Cd500	M	Cd	C	0.04725	0.0201	0.0023	99.313	1.6101	2.8269	2.8429	165.4916
M1Cd500	M	Cd	C	0.04725	0.017	0.002	114.269	1.599	2.7019	2.8622	161.9763
M1Cd500	M	Cd	C	0.04725	0.0171	0.002	92.978	1.4935	2.7284	2.8301	161.2095
M1Cd500	M	Cd	C	0.04725	0.0172	0.002	116.41	1.7043	2.7764	2.8494	167.7099
M1Cd1000	M	Cd	D	0.0945	0.0113	0.002	168.624	1.4115	4.6546	3.9543	108.7095
M1Cd1000	M	Cd	D	0.0095	0.0413	0.0075	145.245	1.0061	4.4933	3.8178	108.9975
M1Cd1000	M	Cd	D	0.0945	0.0343	0.0062	132.004	1.284	4.8772	3.9236	110.9742
M1Cd1000	M	Cd	D	0.0945	0.0287	0.0052	171.298	1.2973	4.7006	3.7871	109.5604
M1Cd1000	M	Cd	D	0.0945	0.0289	0.0052	145.95	1.3976	4.6929	3.9406	99.8641
M1Cd1000	M	Cd	D	0.0945	0.0294	0.0054	127.12	0.9928	4.5496	3.8042	118.6866
M1control	M	control	0	0	1.3754	0.1659	39.814	1.7307	4.9226	4.9936	188.2598
M1control	M	control	0	0	1.2034	0.1451	41.094	1.7316	5.1886	4.5164	190.2882
M1control	M	control	0	0	0.9924	0.1197	38.534	1.7253	5.2031	5.0226	191.4813
M1control	M	control	0	0	1.19	0.1435	40.446	1.7645	4.913	4.4874	190.0098
M1control	M	control	0	0	1.1903	0.1436	39.182	1.7698	5.1886	5.0097	189.779
M1control	M	control	0	0	1.2473	0.1473	38.955	1.7664	5.3095	4.5035	205.8039
M1Zn0	M	Zn	A	0.28	0.0683	0.008	549.538	1.2058	6.8377	3.3266	266.9789
M1Zn0	M	Zn	A	0.28	0.2503	0.0292	603.425	0.9707	7.4191	3.2432	267.6546

M1Zn0	M	Zn	A	0.28	0.1883	0.0219	495.652	1.1759	7.4317	3.2659	272.6303
M1Zn0	M	Zn	A	0.28	0.1687	0.0196	558.227	1.1628	7.4317	3.6047	269.0879
M1Zn0	M	Zn	A	0.28	0.1689	0.0197	540.849	1.1921	6.8099	3.367	245.0221
M1Zn0	M	Zn	A	0.28	0.1748	0.0204	501.444	1.0015	7.3635	3.0814	271.1413
M1Zn2	M	Zn	B	0.56	0.1032	0.0143	862.961	1.5728	8.3991	3.4491	146.7255
M1Zn2	M	Zn	B	0.56	0.0932	0.0129	962.39	1.8601	8.3563	3.4657	147.0479
M1Zn2	M	Zn	B	0.56	0.0962	0.0133	763.531	1.7103	8.9439	3.2564	149.3669
M1Zn2	M	Zn	B	0.56	0.0973	0.0135	882.774	1.7355	8.8392	3.4134	147.7134
M1Zn2	M	Zn	B	0.56	0.0975	0.0135	843.147	1.5347	8.4396	3.1993	141.4312
M1Zn2	M	Zn	B	0.56	0.1022	0.0142	776.741	1.813	8.4087	3.2374	160.5438
M1Zn4	M	Zn	C	1.12	0.0353	0.0105	779.756	2.1931	11.7256	6.1471	130.2839
M1Zn4	M	Zn	C	1.12	0.0433	0.0129	876.366	2.077	11.6554	6.0842	130.7257
M1Zn4	M	Zn	C	1.12	0.0483	0.0143	683.147	2.0697	11.7352	5.8712	133.6078
M1Zn4	M	Zn	C	1.12	0.042	0.0125	841.299	2.0857	11.5731	6.3529	131.5391
M1Zn4	M	Zn	C	1.12	0.0422	0.0126	718.213	2.1839	11.7522	6.3238	119.6802
M1Zn4	M	Zn	C	1.12	0.0439	0.0131	724.175	2.0707	11.7352	5.9414	134.7695
M1Zn10	M	Zn	D	2.8	0.0152	0.0043	866.48	3.0967	16.6458	8.9404	71.1039
M1Zn10	M	Zn	D	2.8	0.0192	0.0055	953.436	3.7129	16.6107	8.6147	71.3753
M1Zn10	M	Zn	D	2.8	0.0243	0.0069	679.523	3.237	17.0441	8.4018	72.5071
M1Zn10	M	Zn	D	2.8	0.0194	0.0055	872.576	3.25	16.8763	8.7901	71.6621
M1Zn10	M	Zn	D	2.8	0.0195	0.0056	860.384	3.0767	16.6182	8.8327	67.7806
M1Zn10	M	Zn	D	2.8	0.0196	0.0057	683.587	3.6804	16.7209	8.73	78.1805
N1control	N	control	0	0	1.1002	0.17	3.773	1.4669	6.7682	3.8385	205.132
N1control	N	control	0	0	0.9827	0.1518	4.045	1.4524	6.7775	3.9256	207.41
N1control	N	control	0	0	1.0525	0.1626	3.501	1.4598	6.7812	3.8385	209.6863
N1control	N	control	0	0	1.0451	0.1615	4.039	1.4691	6.8818	3.9282	207.4095
N1control	N	control	0	0	1.0451	0.1615	3.507	1.4531	6.8901	3.8122	192.9186
N1control	N	control	0	0	1.0477	0.1616	3.679	1.4623	6.8873	3.9071	210.5805
N1Cu1/2	N	Cu	A	0.13	0.0239	0.0035	5.043	1.4105	7.7964	3.697	190.2515
N1Cu1/2	N	Cu	A	0.13	0.0033	0.0005	5.252	1.4115	7.8064	3.7282	183.7073
N1Cu1/2	N	Cu	A	0.13	0.0115	0.0017	4.834	1.4138	7.8034	3.7168	197.5748

N1Cu1/2	N	Cu	A	0.13	0.0129	0.0019	5.286	1.4403	7.8073	3.7083	190.4064
N1Cu1/2	N	Cu	A	0.13	0.0129	0.0019	4.801	1.4409	7.8183	3.68	179.9286
N1Cu1/2	N	Cu	A	0.13	0.0134	0.0019	4.996	1.4439	7.8153	3.6998	203.4847
N1Cu0	N	Cu	B	0.26	0.0012	0.0002	11.84	1.3945	8.1627	3.3503	221.4374
N1Cu0	N	Cu	B	0.26	0.0007	0.0001	12.2	1.4444	8.1706	2.9082	221.0823
N1Cu0	N	Cu	B	0.26	0.001	0.0001	11.48	1.4196	8.1696	3.3872	226.293
N1Cu0	N	Cu	B	0.26	0.001	0.0001	13.182	1.3962	8.1052	2.9365	222.9376
N1Cu0	N	Cu	B	0.26	0.001	0.0001	10.497	1.4468	8.1121	3.3447	212.8025
N1Cu0	N	Cu	B	0.26	0.001	0.0001	12.374	1.4216	8.1171	2.8968	225.075
N1Cu2.5	N	Cu	C	0.65	0.0011	0.0002	22.675	1.4701	7.9586	3.0695	203.399
N1Cu2.5	N	Cu	C	0.65	0.0009	0.0003	23.337	1.5209	7.9661	2.9324	203.4315
N1Cu2.5	N	Cu	C	0.65	0.0008	0.0002	22.013	1.498	7.9605	3.0722	203.5758
N1Cu2.5	N	Cu	C	0.65	0.0007	0.0002	25.415	1.4719	8.1223	2.9243	203.4688
N1Cu2.5	N	Cu	C	0.65	0.0009	0.0002	19.936	1.5212	8.1308	3.0426	187.8408
N1Cu2.5	N	Cu	C	0.65	0.0009	0.0002	23.84	1.4986	8.1383	2.9217	214.2905
N1Cu5	N	Cu	D	1.3	0.0004	0.0001	241.268	3.3833	10.8166	8.9224	128.0919
N1Cu5	N	Cu	D	1.3	0.0006	0.0001	242.959	3.7269	10.8175	8.7056	125.6997
N1Cu5	N	Cu	D	1.3	0.0009	0.0002	239.577	3.5586	10.8248	8.9457	132.8081
N1Cu5	N	Cu	D	1.3	0.0006	0.0001	257.104	3.3848	10.4634	8.7237	128.8666
N1Cu5	N	Cu	D	1.3	0.0006	0.0001	225.431	3.7275	10.4706	8.9018	120.3093
N1Cu5	N	Cu	D	1.3	0.0007	0.0001	250.134	3.5601	10.4652	8.6876	129.9197
N1control	N	control	0	0	1.1002	0.17	1.108	1.4669	6.7682	3.8385	205.132
N1control	N	control	0	0	0.9827	0.1518	1.133	1.4524	6.7775	3.9256	207.41
N1control	N	control	0	0	1.0525	0.1626	1.083	1.4598	6.7812	3.8385	209.6863
N1control	N	control	0	0	1.0451	0.1615	1.241	1.4691	6.8818	3.9282	207.4095
N1control	N	control	0	0	1.0451	0.1615	0.976	1.4531	6.8901	3.8122	192.9186
N1control	N	control	0	0	1.0477	0.1616	1.172	1.4623	6.8873	3.9071	210.5805
N1Pb0	N	Pb	A	0.025	0.9367	0.1988	0.989	2.0065	6.5177	5.3571	201.5578
N1Pb0	N	Pb	A	0.025	0.4463	0.0947	1.064	2.0757	6.5228	5.2727	201.6867
N1Pb0	N	Pb	A	0.025	0.5676	0.1205	0.914	2.0431	6.5245	5.3644	202.7714
N1Pb0	N	Pb	A	0.025	0.6502	0.138	1.092	2.0084	6.8767	5.2968	202.0053

N1Pb0	N	Pb	A	0.025	0.6502	0.138	0.887	2.0777	6.8843	5.3378	186.0825
N1Pb0	N	Pb	A	0.025	0.6788	0.1436	0.983	2.0442	6.8826	5.2606	203.1734
N1Pb10	N	Pb	B	0.25	0.0449	0.0067	27.131	2.3117	8.3053	6.1941	199.2911
N1Pb10	N	Pb	B	0.25	0.0399	0.0059	27.148	2.3368	8.3101	6.2759	199.6237
N1Pb10	N	Pb	B	0.25	0.0295	0.0044	27.114	2.3273	8.3111	6.2105	204.3446
N1Pb10	N	Pb	B	0.25	0.0381	0.0057	28.547	2.313	7.7147	6.2732	201.0865
N1Pb10	N	Pb	B	0.25	0.0381	0.0057	25.716	2.3375	7.7176	6.1832	185.2876
N1Pb10	N	Pb	B	0.25	0.0394	0.0058	28.058	2.3276	7.7156	6.2486	219.6845
N1Pb50	N	Pb	C	1.25	0.0096	0.0019	245.782	1.5964	7.0982	3.7571	199.6957
N1Pb50	N	Pb	C	1.25	0.0043	0.0008	246.508	1.7338	7.0982	3.7474	193.8567
N1Pb50	N	Pb	C	1.25	0.0038	0.0007	245.056	1.6678	7.1075	3.7498	201.245
N1Pb50	N	Pb	C	1.25	0.0059	0.0011	264.73	1.5981	7.4428	3.7886	198.2658
N1Pb50	N	Pb	C	1.25	0.0059	0.0011	226.834	1.7361	7.4513	3.7378	191.638
N1Pb50	N	Pb	C	1.25	0.0061	0.0012	257.688	1.6698	7.4564	3.7426	214.1936
N1Pb100	N	Pb	D	2.5	0.0011	0.0002	983.2	1.5733	8.9846	4.3777	179.8746
N1Pb100	N	Pb	D	2.5	0.0037	0.0007	983.72	1.7788	8.9873	4.4761	180.4573
N1Pb100	N	Pb	D	2.5	0.0035	0.0007	982.68	1.6769	8.9882	4.4165	181.2841
N1Pb100	N	Pb	D	2.5	0.0028	0.0006	1100.352	1.5757	8.8123	4.4942	180.5387
N1Pb100	N	Pb	D	2.5	0.0028	0.0006	866.044	1.7806	8.8214	4.3725	178.371
N1Pb100	N	Pb	D	2.5	0.0029	0.0006	904.576	1.6769	8.8241	4.4528	192.5074
N1control	N	control	0	0	1.1002	0.17	0.248	1.4669	6.7682	3.8385	205.132
N1control	N	control	0	0	0.9827	0.1518	0.217	1.4524	6.7775	3.9256	207.41
N1control	N	control	0	0	1.0525	0.1626	0.259	1.4598	6.7812	3.8385	209.6863
N1control	N	control	0	0	1.0451	0.1615	0.266	1.4691	6.8818	3.9282	207.4095
N1control	N	control	0	0	1.0451	0.1615	0.211	1.4531	6.8901	3.8122	192.9186
N1control	N	control	0	0	1.0477	0.1616	0.214	1.4623	6.8873	3.9071	210.5805
N1Cr0	N	Cr	A	0.036	0.0752	0.0086	1.55	2.2343	7.1553	2.2031	222.5209
N1Cr0	N	Cr	A	0.036	0.2067	0.0235	1.58	2.2014	7.1589	2.3621	221.6588
N1Cr0	N	Cr	A	0.036	0.0768	0.0087	1.52	2.2208	7.1724	2.2083	225.1014
N1Cr0	N	Cr	A	0.036	0.1196	0.0136	1.809	2.2352	6.4031	2.3519	223.0937
N1Cr0	N	Cr	A	0.036	0.1196	0.0136	1.291	2.2014	6.4093	2.198	218.9022

N1Cr0	N	Cr	A	0.036	0.1249	0.014	1.347	2.2211	6.4093	2.3391	224.0356
N1Cr5	N	Cr	B	0.18	0.0448	0.0073	2.75	2.8174	7.1504	2.7938	191.4531
N1Cr5	N	Cr	B	0.18	0.0972	0.0159	2.771	2.7637	7.1562	2.7166	183.4475
N1Cr5	N	Cr	B	0.18	0.0536	0.0087	2.729	2.7949	7.163	2.802	197.2065
N1Cr5	N	Cr	B	0.18	0.0652	0.0106	3.015	2.8184	7.2883	2.7139	190.7024
N1Cr5	N	Cr	B	0.18	0.0652	0.0106	2.486	2.765	7.2922	2.769	172.0433
N1Cr5	N	Cr	B	0.18	0.0684	0.011	2.553	2.7952	7.3086	2.6918	195.2958
N1Cr10	N	Cr	C	0.36	0.0055	0.001	2.871	2.6489	9.7523	2.6393	220.2714
N1Cr10	N	Cr	C	0.36	0.0094	0.0017	2.893	2.5894	9.7603	2.9718	214.5111
N1Cr10	N	Cr	C	0.36	0.0215	0.0039	2.849	2.6222	9.7673	2.6651	220.8109
N1Cr10	N	Cr	C	0.36	0.0121	0.0022	2.995	2.6513	9.2467	2.9976	218.5312
N1Cr10	N	Cr	C	0.36	0.0121	0.0022	2.746	2.5908	9.2578	2.6107	207.1642
N1Cr10	N	Cr	C	0.36	0.0127	0.0023	2.766	2.6239	9.2477	2.9431	232.5463
N1Cr25	N	Cr	D	0.90	0.0231	0.0047	7.819	2.7344	10.0943	5.6893	195.9682
N1Cr25	N	Cr	D	0.90	0.006	0.0012	7.868	3.633	10.0962	5.9836	202.861
N1Cr25	N	Cr	D	0.90	0.0258	0.0053	7.77	3.1863	10.1088	5.7393	195.9817
N1Cr25	N	Cr	D	0.90	0.0183	0.0037	8.093	2.737	10.1953	6.0197	198.2703
N1Cr25	N	Cr	D	0.90	0.0183	0.0037	7.545	3.6339	10.1992	5.6837	187.8056
N1Cr25	N	Cr	D	0.90	0.0187	0.0038	7.588	3.1885	10.2148	5.9642	217.0864
N1control	N	control	0	0	1.1002	0.17	1.476	1.4669	6.7682	3.8385	205.132
N1control	N	control	0	0	0.9827	0.1518	1.576	1.4524	6.7775	3.9256	207.41
N1control	N	control	0	0	1.0525	0.1626	1.376	1.4598	6.7812	3.8385	209.6863
N1control	N	control	0	0	1.0451	0.1615	1.592	1.4691	6.8818	3.9282	207.4095
N1control	N	control	0	0	1.0451	0.1615	1.359	1.4531	6.8901	3.8122	192.9186
N1control	N	control	0	0	1.0477	0.1616	1.298	1.4623	6.8873	3.9071	210.5805
N1Ni0	N	Ni	A	0.025	0.2149	0.0249	9.867	1.6154	6.8	3.6444	235.8319
N1Ni0	N	Ni	A	0.025	0.3117	0.0361	10.067	1.6201	6.808	3.4489	233.0011
N1Ni0	N	Ni	A	0.025	0.3424	0.0396	9.667	1.619	6.8133	3.6343	237.1401
N1Ni0	N	Ni	A	0.025	0.2897	0.0335	9.903	1.616	6.7831	3.4895	235.3244
N1Ni0	N	Ni	A	0.025	0.2897	0.0335	9.83	1.6204	6.7867	3.6317	234.2604
N1Ni0	N	Ni	A	0.025	0.2947	0.0347	9.642	1.621	6.7884	3.4463	247.9634

N1Ni5	N	Ni	B	0.125	0.0449	0.0056	25.47	2.6044	7.1591	6.4676	195.7175
N1Ni5	N	Ni	B	0.125	0.0445	0.0056	26.235	2.6223	7.1647	6.1398	194.1259
N1Ni5	N	Ni	B	0.125	0.0361	0.0045	24.705	2.6161	7.1665	6.4834	200.7363
N1Ni5	N	Ni	B	0.125	0.0418	0.0052	27.539	2.6059	7.062	6.1504	196.8599
N1Ni5	N	Ni	B	0.125	0.0418	0.0052	23.401	2.6241	7.0648	6.4517	178.4052
N1Ni5	N	Ni	B	0.125	0.0433	0.0053	23.326	2.617	7.0731	6.1398	208.8991
N1Ni10	N	Ni	C	0.25	0.0345	0.0045	51.851	2.1752	7.519	5.5737	184.9425
N1Ni10	N	Ni	C	0.25	0.0142	0.0019	51.957	2.2108	7.519	5.6825	183.5937
N1Ni10	N	Ni	C	0.25	0.0217	0.0029	51.745	2.1962	7.5237	5.6029	187.994
N1Ni10	N	Ni	C	0.25	0.0235	0.0031	54.587	2.177	7.9777	5.6878	185.5101
N1Ni10	N	Ni	C	0.25	0.0235	0.0031	49.116	2.212	7.9786	5.5578	175.5178
N1Ni10	N	Ni	C	0.25	0.0246	0.0032	49.922	2.1984	7.9833	5.6586	186.4576
N1Ni25	N	Ni	D	0.625	0.0074	0.0012	55.915	1.9684	7.8939	4.9747	180.6169
N1Ni25	N	Ni	D	0.625	0.0024	0.0004	56.042	2.107	7.8999	5.0659	176.775
N1Ni25	N	Ni	D	0.625	0.019	0.0032	55.788	2.0397	7.9051	4.9624	185.7599
N1Ni25	N	Ni	D	0.625	0.0096	0.0016	56.758	1.9692	8.3175	5.0684	181.0506
N1Ni25	N	Ni	D	0.625	0.0096	0.0016	55.073	2.1076	8.3279	4.9501	166.5402
N1Ni25	N	Ni	D	0.625	0.0101	0.0016	55.227	2.0411	8.3244	5.0561	195.9673
N1control	N	control	0	0	1.1002	0.17	1.382	1.4669	6.7682	3.8385	205.132
N1control	N	control	0	0	0.9827	0.1518	1.418	1.4524	6.7775	3.9256	207.41
N1control	N	control	0	0	1.0525	0.1626	1.346	1.4598	6.7812	3.8385	209.6863
N1control	N	control	0	0	1.0451	0.1615	1.515	1.4691	6.8818	3.9282	207.4095
N1control	N	control	0	0	1.0451	0.1615	1.248	1.4531	6.8901	3.8122	192.9186
N1control	N	control	0	0	1.0477	0.1616	1.257	1.4623	6.8873	3.9071	210.5805
N1Cd0	N	Cd	A	0.0000945	0.7106	0.1071	1.593	1.8077	7.1083	0.7175	229.2065
N1Cd0	N	Cd	A	0.0000945	0.5523	0.0833	1.729	1.3188	7.1119	0.7227	230.4884
N1Cd0	N	Cd	A	0.0000945	0.3446	0.052	1.457	1.5638	7.1156	0.7201	234.7013
N1Cd0	N	Cd	A	0.0000945	0.5358	0.0808	1.736	1.8095	6.4959	0.7149	231.4654
N1Cd0	N	Cd	A	0.0000945	0.5358	0.0808	1.45	1.3212	6.4986	0.7097	215.3815
N1Cd0	N	Cd	A	0.0000945	0.5416	0.0831	1.361	1.5648	6.5014	0.7071	244.4855
N1Cd100	N	Cd	B	0.00945	0.2122	0.0134	15.348	1.5329	7.4956	1.8022	187.8877

N1Cd100	N	Cd	B	0.00945	0.1862	0.0117	15.508	1.409	7.5	1.8302	187.7021
N1Cd100	N	Cd	B	0.00945	0.2089	0.0131	15.188	1.4748	7.508	1.8149	192.3576
N1Cd100	N	Cd	B	0.00945	0.2024	0.0127	17.458	1.5329	7.1708	1.8277	189.3158
N1Cd100	N	Cd	B	0.00945	0.2024	0.0127	13.238	1.4104	7.1806	1.787	177.7535
N1Cd100	N	Cd	B	0.00945	0.2082	0.0128	13.781	1.4757	7.1735	1.8149	195.7408
N1Cd500	N	Cd	C	0.04725	0.0222	0.0029	81.906	1.3895	8.9458	0.8464	164.5424
N1Cd500	N	Cd	C	0.04725	0.0754	0.0097	82.142	1.5244	8.9574	0.7661	172.2558
N1Cd500	N	Cd	C	0.04725	0.147	0.019	81.67	1.4595	8.9632	0.8878	161.8696
N1Cd500	N	Cd	C	0.04725	0.0815	0.0105	88.611	1.3895	10.2091	0.8049	166.2226
N1Cd500	N	Cd	C	0.04725	0.0815	0.0105	75.2	1.5257	10.2101	0.8408	159.181
N1Cd500	N	Cd	C	0.04725	0.0818	0.011	77.199	1.4598	10.2294	0.7523	178.6776
N1Cd1000	N	Cd	D	0.0945	0.0154	0.0039	146.993	1.1983	9.7627	0.4114	159.75
N1Cd1000	N	Cd	D	0.0095	0.0127	0.0032	147.313	1.1705	9.7706	0.2554	162.0061
N1Cd1000	N	Cd	D	0.0945	0.0287	0.0072	146.673	1.1857	9.7706	0.4369	161.477
N1Cd1000	N	Cd	D	0.0945	0.0189	0.0047	166.379	1.1996	9.4945	0.2809	161.0777
N1Cd1000	N	Cd	D	0.0945	0.0189	0.0048	127.607	1.1711	9.4985	0.4001	146.1475
N1Cd1000	N	Cd	D	0.0945	0.0195	0.005	133.749	1.1874	9.4945	0.2412	168.9726
N1control	N	control	0	0	1.1002	0.17	25.681	1.4669	6.7682	3.8385	205.132
N1control	N	control	0	0	0.9827	0.1518	26.704	1.4524	6.7775	3.9256	207.41
N1control	N	control	0	0	1.0525	0.1626	24.658	1.4598	6.7812	3.8385	209.6863
N1control	N	control	0	0	1.0451	0.1615	27.342	1.4691	6.8818	3.9282	207.4095
N1control	N	control	0	0	1.0451	0.1615	24.02	1.4531	6.8901	3.8122	192.9186
N1control	N	control	0	0	1.0477	0.1616	23.551	1.4623	6.8873	3.9071	210.5805
N1Zn0	N	Zn	A	0.28	0.1689	0.0206	243.43	1.9376	7.5836	5.239	224.4993
N1Zn0	N	Zn	A	0.28	0.1306	0.0159	243.732	1.9758	7.5864	5.2	226.7271
N1Zn0	N	Zn	A	0.28	0.0575	0.007	243.128	1.957	7.5991	5.2494	227.1877
N1Zn0	N	Zn	A	0.28	0.119	0.0145	265.806	1.9394	8.5682	5.1896	226.1381
N1Zn0	N	Zn	A	0.28	0.119	0.0145	221.053	1.9773	8.5745	5.226	205.3287
N1Zn0	N	Zn	A	0.28	0.1195	0.0148	228.21	1.9582	8.5773	5.187	229.4104
N1Zn2	N	Zn	B	0.56	0.0936	0.0149	377.157	2.0568	8.5502	4.4854	211.8943
N1Zn2	N	Zn	B	0.56	0.5194	0.0824	377.802	1.9537	8.5547	5	210.9241

N1Zn2	N	Zn	B	0.56	0.1086	0.0172	376.512	2.0066	8.5547	4.5059	213.7796
N1Zn2	N	Zn	B	0.56	0.2405	0.0382	395.751	2.0579	9.3154	5.0026	212.1993
N1Zn2	N	Zn	B	0.56	0.2405	0.0382	358.564	1.9543	9.3244	4.4598	198.2282
N1Zn2	N	Zn	B	0.56	0.2481	0.0397	364.117	2.0069	9.3333	4.9872	214.4053
N1Zn4	N	Zn	C	1.12	0.0138	0.0047	356.836	2.175	8.4389	5.2692	175.5832
N1Zn4	N	Zn	C	1.12	0.0077	0.0026	357.127	2.1512	8.4458	4.9103	171.5925
N1Zn4	N	Zn	C	1.12	0.0208	0.0071	356.545	2.1638	8.4518	5.311	178.4714
N1Zn4	N	Zn	C	1.12	0.0141	0.0048	382.641	2.1756	9.3812	4.8857	175.2157
N1Zn4	N	Zn	C	1.12	0.0141	0.0048	331.03	2.1517	9.3838	5.2692	174.1034
N1Zn4	N	Zn	C	1.12	0.0146	0.005	339.341	2.1655	9.3898	4.8857	184.9066
N1Zn10	N	Zn	D	2.8	0.0008	0.0003	511.377	3.0698	11.2075	9.1756	110.0157
N1Zn10	N	Zn	D	2.8	0.0005	0.0002	511.493	3.0267	11.2164	9.7346	111.0437
N1Zn10	N	Zn	D	2.8	0.0008	0.0003	511.261	3.0484	11.2105	9.2095	109.939
N1Zn10	N	Zn	D	2.8	0.0007	0.0002	525.398	3.0705	11.3557	9.7487	110.3328
N1Zn10	N	Zn	D	2.8	0.0007	0.0003	497.356	3.029	11.3557	9.17	104.5272
N1Zn10	N	Zn	D	2.8	0.0007	0.0003	520.608	3.0507	11.3666	9.7205	116.8031
T1control	T	control	0	0	1.25	0.1432	5.725	1.4055	6.171	2.3049	237.6365
T1control	T	control	0	0	1.102	0.1263	5.845	1.2841	6.1727	2.408	230.196
T1control	T	control	0	0	0.092	0.0105	5.605	1.3471	6.1813	2.3466	239.4143
T1control	T	control	0	0	0.8147	0.0933	6.454	1.4069	5.8711	2.435	235.7489
T1control	T	control	0	0	0.8147	0.0933	4.996	1.2864	5.8771	2.3024	217.3781
T1control	T	control	0	0	0.8459	0.0948	6.091	1.3482	5.8883	2.3932	252.1255
T1Cu1/2	T	Cu	A	0.13	0.0234	0.003	36.007	1.4797	4.082	2.5891	184.8072
T1Cu1/2	T	Cu	A	0.13	0.0265	0.0034	37.517	1.4812	4.0847	2.6958	206.8857
T1Cu1/2	T	Cu	A	0.13	0.0334	0.0042	34.497	1.4836	4.0984	2.6151	191.9433
T1Cu1/2	T	Cu	A	0.13	0.0274	0.0035	37.015	1.4885	3.5556	2.714	201.1056
T1Cu1/2	T	Cu	A	0.13	0.0277	0.0035	34.999	1.4924	3.5583	2.5813	179.4373
T1Cu1/2	T	Cu	A	0.13	0.0289	0.0036	35.169	1.4885	3.571	2.6932	200.1888
T1Cu0	T	Cu	B	0.26	0.0161	0.003	85.155	1.6306	4.6379	1.5736	181.8072
T1Cu0	T	Cu	B	0.26	0.0041	0.0008	85.254	1.7205	4.6497	1.7815	183.7073
T1Cu0	T	Cu	B	0.26	0.0071	0.0013	85.056	1.6757	4.6481	1.5833	186.6018

T1Cu0	T	Cu	B	0.26	0.009	0.0017	90.004	1.6328	5.0017	1.7887	184.0387
T1Cu0	T	Cu	B	0.26	0.0091	0.0017	80.306	1.7208	5.0059	1.5712	177.7457
T1Cu0	T	Cu	B	0.26	0.0095	0.0018	88.289	1.6771	5.0068	1.7815	197.1888
T1Cu2.5	T	Cu	C	0.65	0.0122	0.0024	328.426	2.8418	6.5599	5.4147	129.766
T1Cu2.5	T	Cu	C	0.65	0.0072	0.0014	328.889	2.8443	6.5693	5.527	130.0078
T1Cu2.5	T	Cu	C	0.65	0.0052	0.001	327.963	2.8446	6.5758	5.4334	129.7409
T1Cu2.5	T	Cu	C	0.65	0.008	0.0016	351.705	2.8421	6.0159	5.5511	129.8383
T1Cu2.5	T	Cu	C	0.65	0.0082	0.0016	305.147	2.843	6.0187	5.4013	124.0492
T1Cu2.5	T	Cu	C	0.65	0.0085	0.0017	343.482	2.8443	6.0243	5.511	139.4344
T1Cu5	T	Cu	D	1.3	0.0021	0.0005	369.026	3.6133	8.6728	7.1318	59.7822
T1Cu5	T	Cu	D	1.3	0.0061	0.0014	336.0047	3.6136	8.6805	7.0491	58.2968
T1Cu5	T	Cu	D	1.3	0.0041	0.0009	420.0218	3.6133	8.6863	7.1594	62.5833
T1Cu5	T	Cu	D	1.3	0.004	0.0009	492.8164	3.5351	8.916	7.0463	63.3269
T1Cu5	T	Cu	D	1.3	0.0041	0.0009	355.4572	3.5389	8.9257	7.1263	59.1836
T1Cu5	T	Cu	D	1.3	0.0042	0.001	377.0435	3.5373	8.9305	7.0353	56.745
T1control	T	control	0	0	1.25	0.1432	0.297	1.4055	6.171	2.3049	237.6365
T1control	T	control	0	0	1.102	0.1263	0.323	1.2841	6.1727	2.408	230.196
T1control	T	control	0	0	0.092	0.0105	0.271	1.3471	6.1813	2.3466	239.4143
T1control	T	control	0	0	0.8147	0.0933	0.397	1.4069	5.8711	2.435	235.7489
T1control	T	control	0	0	0.8147	0.0933	0.297	1.2864	5.8771	2.3024	217.3781
T1control	T	control	0	0	0.8459	0.0948	0.337	1.3482	5.8883	2.3932	252.1255
T1Pb0	T	Pb	A	0.025	0.268	0.0411	0.495	2.1026	7.1616	4.832	229.6723
T1Pb0	T	Pb	A	0.025	0.321	0.0492	0.416	2.1144	7.1683	4.7473	226.6263
T1Pb0	T	Pb	A	0.025	0.224	0.0344	0.474	2.1122	7.1692	4.8156	231.2384
T1Pb0	T	Pb	A	0.025	0.271	0.0416	0.505	2.1029	7.7782	4.7774	229.179
T1Pb0	T	Pb	A	0.025	0.271	0.0416	0.485	2.1166	7.7878	4.8156	213.2224
T1Pb0	T	Pb	A	0.025	0.2785	0.0431	0.381	2.1147	7.7811	4.7446	238.6075
T1Pb10	T	Pb	B	0.25	0.1251	0.018	38.6	1.4426	5.8151	2.617	210.3221
T1Pb10	T	Pb	B	0.25	0.1691	0.0243	46.7	1.4345	5.8245	2.7478	209.2471
T1Pb10	T	Pb	B	0.25	0.1841	0.0264	46.5	1.4413	5.8263	2.6411	212.2549
T1Pb10	T	Pb	B	0.25	0.1593	0.0229	45.4	1.4444	5.7526	2.7664	210.608

T1Pb10	T	Pb	B	0.25	0.1594	0.0229	41.9	1.4357	5.7572	2.593	203.2306
T1Pb10	T	Pb	B	0.25	0.1613	0.0234	44.4	1.4435	5.7666	2.7371	214.3487
T1Pb50	T	Pb	C	1.25	0.0113	0.0018	275.243	3.4808	9.819	7.8846	181.8703
T1Pb50	T	Pb	C	1.25	0.0193	0.003	284.7985	3.5704	9.8218	7.8584	175.9568
T1Pb50	T	Pb	C	1.25	0.0153	0.0024	262.2333	3.5274	9.8199	7.9002	184.3313
T1Pb50	T	Pb	C	1.25	0.015	0.0024	295.8498	3.4832	9.7441	7.895	180.7195
T1Pb50	T	Pb	C	1.25	0.0152	0.0024	254.8965	3.5722	9.7486	7.8663	176.0813
T1Pb50	T	Pb	C	1.25	0.0159	0.0025	262.4166	3.5277	9.7578	7.848	191.3379
T1Pb100	T	Pb	D	2.5	0.0092	0.0017	937.7676	5.2531	12.798	10.9442	123.2534
T1Pb100	T	Pb	D	2.5	0.0132	0.0024	964.786	5.3008	12.803	10.4017	126.086
T1Pb100	T	Pb	D	2.5	0.0042	0.0008	941.7514	5.2816	12.802	10.9703	126.1388
T1Pb100	T	Pb	D	2.5	0.0087	0.0016	1053.6386	5.2538	12.8203	10.4249	125.1594
T1Pb100	T	Pb	D	2.5	0.0088	0.0016	773.1527	5.3032	12.8244	10.9442	113.1492
T1Pb100	T	Pb	D	2.5	0.0091	0.0017	833.2952	5.2839	12.8284	10.3988	127.8826
T1control	T	control	0	0	1.25	0.1432	1.687	1.4055	6.171	2.3049	237.6365
T1control	T	control	0	0	1.102	0.1263	1.725	1.2841	6.1727	2.408	230.196
T1control	T	control	0	0	0.092	0.0105	1.649	1.3471	6.1813	2.3466	239.4143
T1control	T	control	0	0	0.8147	0.0933	1.742	1.4069	5.8711	2.435	235.7489
T1control	T	control	0	0	0.8147	0.0933	1.632	1.2864	5.8771	2.3024	217.3781
T1control	T	control	0	0	0.8459	0.0948	1.686	1.3482	5.8883	2.3932	252.1255
T1Cr0	T	Cr	A	0.036	0.8514	0.0793	2.068	1.9	4.3058	1.2308	222.9126
T1Cr0	T	Cr	A	0.036	0.9544	0.0889	2.181	1.9151	4.3077	1.2473	221.7864
T1Cr0	T	Cr	A	0.036	1.0104	0.0941	1.955	1.908	4.3212	1.228	226.2947
T1Cr0	T	Cr	A	0.036	0.9383	0.0874	2.204	1.9016	3.4087	1.2775	223.6645
T1Cr0	T	Cr	A	0.036	0.9386	0.0874	1.932	1.916	3.4096	1.2225	213.0934
T1Cr0	T	Cr	A	0.036	0.9788	0.0896	2.045	1.909	3.4163	1.2335	240.5534
T1Cr5	T	Cr	B	0.18	0.1052	0.0149	6.073	1.986	4.8691	1.3644	251.3646
T1Cr5	T	Cr	B	0.18	0.1192	0.0169	6.1	1.9502	4.8701	1.3253	247.9893
T1Cr5	T	Cr	B	0.18	0.1062	0.0151	6.046	1.9691	4.875	1.3644	254.0301
T1Cr5	T	Cr	B	0.18	0.11	0.0156	6.136	1.986	3.9395	1.3421	251.128
T1Cr5	T	Cr	B	0.18	0.1102	0.0156	6.01	1.9515	3.9424	1.3532	245.8746

T1Cr5	T	Cr	B	0.18	0.1113	0.0162	6.088	1.9701	3.9453	1.303	256.3627
T1Cr10	T	Cr	C	0.36	0.0264	0.0031	9.451	2.4986	6.3045	3.2347	230.5907
T1Cr10	T	Cr	C	0.36	0.0154	0.0018	9.516	2.7697	6.3155	3.4853	235.8436
T1Cr10	T	Cr	C	0.36	0.0144	0.0017	9.386	2.6366	6.3174	3.2479	234.1573
T1Cr10	T	Cr	C	0.36	0.0183	0.0022	9.525	2.5005	5.4572	3.4853	233.5305
T1Cr10	T	Cr	C	0.36	0.0186	0.0022	9.378	2.7712	5.4673	3.219	213.8135
T1Cr10	T	Cr	C	0.36	0.0194	0.0023	9.435	2.6381	5.4747	3.4722	238.736
T1Cr25	T	Cr	D	0.90	0.012	0.0032	141.203	5.6198	11.0269	10.1638	164.8509
T1Cr25	T	Cr	D	0.90	0.008	0.0021	141.715	5.6893	11.0349	10.1837	168.3659
T1Cr25	T	Cr	D	0.90	0.021	0.0056	140.691	5.6567	11.0369	10.2094	166.7035
T1Cr25	T	Cr	D	0.90	0.0137	0.0036	154.71	5.6221	11.0209	10.2094	166.6401
T1Cr25	T	Cr	D	0.90	0.0137	0.0036	127.695	5.6906	11.0319	10.1609	150.0718
T1Cr25	T	Cr	D	0.90	0.0141	0.0037	149.696	5.6577	11.0229	10.178	169.8817
T1control	T	control	0	0	1.25	0.1432	2.135	1.4055	6.171	2.3049	237.6365
T1control	T	control	0	0	1.102	0.1263	2.227	1.2841	6.1727	2.408	230.196
T1control	T	control	0	0	0.092	0.0105	2.043	1.3471	6.1813	2.3466	239.4143
T1control	T	control	0	0	0.8147	0.0933	2.137	1.4069	5.8711	2.435	235.7489
T1control	T	control	0	0	0.8147	0.0933	2.132	1.2864	5.8771	2.3024	217.3781
T1control	T	control	0	0	0.8459	0.0948	2.044	1.3482	5.8883	2.3932	252.1255
T1Ni0	T	Ni	A	0.025	0.1491	0.0146	23.803	1.3995	5.5906	5.6925	216.7767
T1Ni0	T	Ni	A	0.025	0.2191	0.0215	24.229	1.7141	5.5973	5.6543	216.6148
T1Ni0	T	Ni	A	0.025	0.2661	0.0261	23.377	1.5611	5.6011	5.6952	218.9994
T1Ni0	T	Ni	A	0.025	0.2113	0.0207	25.143	1.4001	5.7004	5.657	217.4636
T1Ni0	T	Ni	A	0.025	0.2114	0.0207	22.463	1.7163	5.7071	5.6897	201.7511
T1Ni0	T	Ni	A	0.025	0.2145	0.0215	24.27	1.562	5.7023	5.6407	218.3947
T1Ni5	T	Ni	B	0.125	0.0723	0.0105	184.909	1.2359	5.4835	4.7583	177.6597
T1Ni5	T	Ni	B	0.125	0.0513	0.0074	184.96	1.3296	5.4882	4.8822	174.6947
T1Ni5	T	Ni	B	0.125	0.0543	0.0079	184.858	1.2834	5.4976	4.7502	179.7923
T1Ni5	T	Ni	B	0.125	0.059	0.0085	208.354	1.2375	5.4835	4.8876	177.3822
T1Ni5	T	Ni	B	0.125	0.0592	0.0086	161.464	1.3299	5.4844	4.7448	168.6957
T1Ni5	T	Ni	B	0.125	0.0593	0.009	169.228	1.285	5.4901	4.8714	184.2651

T1Ni10	T	Ni	C	0.25	0.0262	0.004	118.919	2.5725	7.91	5.7441	177.5996
T1Ni10	T	Ni	C	0.25	0.0412	0.0062	119.627	2.5182	7.9194	5.6953	175.6459
T1Ni10	T	Ni	C	0.25	0.0342	0.0052	118.211	2.5491	7.927	5.7712	177.5863
T1Ni10	T	Ni	C	0.25	0.0337	0.0051	119.036	2.5744	8.1422	5.6737	176.944
T1Ni10	T	Ni	C	0.25	0.0338	0.0051	118.802	2.5204	8.1536	5.7414	175.9079
T1Ni10	T	Ni	C	0.25	0.0351	0.0052	118.133	2.5498	8.1573	5.6682	182.9579
T1Ni25	T	Ni	D	0.625	0.0071	0.0012	149.354	2.4332	7.8993	7.0318	146.4202
T1Ni25	T	Ni	D	0.625	0.0051	0.0008	149.484	2.47	7.9037	6.8703	148.1922
T1Ni25	T	Ni	D	0.625	0.0141	0.0023	149.224	2.4544	7.9019	7.0192	150.5743
T1Ni25	T	Ni	D	0.625	0.0087	0.0014	155.967	2.4346	8.4099	6.8627	148.3956
T1Ni25	T	Ni	D	0.625	0.0088	0.0014	142.742	2.4717	8.4134	7.0167	142.2239
T1Ni25	T	Ni	D	0.625	0.009	0.0015	144.816	2.4564	8.4231	6.8501	157.3231
T1control	T	control	0	0	1.25	0.1432	0.352	1.4055	6.171	2.3049	237.6365
T1control	T	control	0	0	1.102	0.1263	0.378	1.2841	6.1727	2.408	230.196
T1control	T	control	0	0	0.092	0.0105	0.326	1.3471	6.1813	2.3466	239.4143
T1control	T	control	0	0	0.8147	0.0933	0.346	1.4069	5.8711	2.435	235.7489
T1control	T	control	0	0	0.8147	0.0933	0.358	1.2864	5.8771	2.3024	217.3781
T1control	T	control	0	0	0.8459	0.0948	0.323	1.3482	5.8883	2.3932	252.1255
T1Cd0	T	Cd	A	0.0000945	0.3632	0.0377	1.807	1.696	3.2578	4.2464	198.6959
T1Cd0	T	Cd	A	0.0000945	0.4092	0.0425	1.813	1.6465	3.267	3.8689	206.8857
T1Cd0	T	Cd	A	0.0000945	0.2582	0.0268	1.801	1.6752	3.2752	4.2647	197.5748
T1Cd0	T	Cd	A	0.0000945	0.3433	0.0357	2.031	1.6966	3.2404	3.8873	201.0522
T1Cd0	T	Cd	A	0.0000945	0.3435	0.0357	1.583	1.6474	3.2468	4.2307	181.1289
T1Cd0	T	Cd	A	0.0000945	0.3585	0.0374	1.652	1.6761	3.2587	3.8506	219.7805
T1Cd100	T	Cd	B	0.00945	0.0613	0.0068	79.916	1.6602	3.4	3.0159	197.8446
T1Cd100	T	Cd	B	0.00945	0.1163	0.0129	80.171	1.45	3.4065	3.0212	193.6952
T1Cd100	T	Cd	B	0.00945	0.0943	0.0105	79.661	1.5552	3.4056	3.0106	198.5452
T1Cd100	T	Cd	B	0.00945	0.0903	0.01	86.26	1.6623	3.1528	3.0238	196.695
T1Cd100	T	Cd	B	0.00945	0.0906	0.0101	63.572	1.4503	3.1611	3.0053	179.7815
T1Cd100	T	Cd	B	0.00945	0.0924	0.0105	68.765	1.5552	3.1574	3.0079	211.4139
T1Cd500	T	Cd	C	0.04725	0.0473	0.0062	454.628	1.1502	3.4966	3.2215	106.0349

T1Cd500	T	Cd	C	0.04725	0.1053	0.0137	455.685	1.6337	3.5053	3.342	106.477
T1Cd500	T	Cd	C	0.04725	0.0253	0.0033	453.571	1.3931	3.5014	3.2297	108.0466
T1Cd500	T	Cd	C	0.04725	0.059	0.0077	467.714	1.1518	3.2531	3.3584	106.8528
T1Cd500	T	Cd	C	0.04725	0.0592	0.0077	441.542	1.6353	3.256	3.2105	104.3214
T1Cd500	T	Cd	C	0.04725	0.0597	0.008	444.847	1.3944	3.2637	3.3338	116.5813
T1Cd1000	T	Cd	D	0.0945	0.0143	0.0033	538.328	2.276	5.6894	7.0907	85.465
T1Cd1000	T	Cd	D	0.0095	0.0073	0.0017	540.106	2.3179	5.6904	6.9176	85.4043
T1Cd1000	T	Cd	D	0.0945	0.0023	0.0005	536.55	2.3	5.7029	7.1236	91.2155
T1Cd1000	T	Cd	D	0.0945	0.0077	0.0018	559.161	2.2769	4.8769	6.9533	87.3616
T1Cd1000	T	Cd	D	0.0945	0.0079	0.0018	517.494	2.3192	4.8856	7.0769	82.4296
T1Cd1000	T	Cd	D	0.0945	0.008	0.0019	522.661	2.3026	4.8962	6.9038	92.5293
T1control	T	control	0	0	1.25	0.1432	29.728	1.4055	6.171	2.3049	237.6365
T1control	T	control	0	0	1.102	0.1263	31.931	1.2841	6.1727	2.408	230.196
T1control	T	control	0	0	0.092	0.0105	27.525	1.3471	6.1813	2.3466	239.4143
T1control	T	control	0	0	0.8147	0.0933	31.282	1.4069	5.8711	2.435	235.7489
T1control	T	control	0	0	0.8147	0.0933	28.175	1.2864	5.8771	2.3024	217.3781
T1control	T	control	0	0	0.8459	0.0948	26.489	1.3482	5.8883	2.3932	252.1255
T1Zn0	T	Zn	A	0.28	0.0752	0.0104	553.47	2.5711	7.6906	6.0008	189.1445
T1Zn0	T	Zn	A	0.28	0.1062	0.0147	555.115	2.492	7.701	5.9576	190.7201
T1Zn0	T	Zn	A	0.28	0.0352	0.0049	551.825	2.533	7.6954	6.0278	191.7493
T1Zn0	T	Zn	A	0.28	0.072	0.01	609.562	2.5736	7.9281	5.9765	190.5379
T1Zn0	T	Zn	A	0.28	0.0722	0.01	497.378	2.4923	7.9385	5.9765	180.0464
T1Zn0	T	Zn	A	0.28	0.0724	0.0101	514.431	2.533	7.9366	5.9332	197.7271
T1Zn2	T	Zn	B	0.56	0.0532	0.0079	1003.147	1.8755	6.9157	4.2663	151.6044
T1Zn2	T	Zn	B	0.56	0.0682	0.0101	1003.519	1.8374	6.9238	4.502	148.5056
T1Zn2	T	Zn	B	0.56	0.0772	0.0114	1002.775	1.858	6.9248	4.2637	151.8552
T1Zn2	T	Zn	B	0.56	0.066	0.0098	1017.795	1.8764	7.0535	4.5175	150.6551
T1Zn2	T	Zn	B	0.56	0.0662	0.0098	988.5	1.8398	7.0617	4.2559	147.5736
T1Zn2	T	Zn	B	0.56	0.0688	0.0099	993.01	1.8598	7.0544	4.4891	158.973
T1Zn4	T	Zn	C	1.12	0.0322	0.0059	1374.456	2.161	8.3725	5.6033	109.601
T1Zn4	T	Zn	C	1.12	0.0492	0.0091	1376.046	2.1524	8.3815	5.6375	108.4899

T1Zn4	T	Zn	C	1.12	0.0242	0.0045	1372.866	2.16	8.3835	5.6118	114.7745
T1Zn4	T	Zn	C	1.12	0.035	0.0064	1444.04	2.1637	8.2998	5.6517	110.9551
T1Zn4	T	Zn	C	1.12	0.0352	0.0065	1304.872	2.1544	8.3118	5.5777	100.6187
T1Zn4	T	Zn	C	1.12	0.0368	0.0066	1326.476	2.16	8.3078	5.609	115.4467
T1Zn10	T	Zn	D	2.8	0.0022	0.0004	2526.246	4.7979	13.5822	11.3516	56.9371
T1Zn10	T	Zn	D	2.8	0.0082	0.0014	2527.981	4.9177	13.585	11.4286	58.2968
T1Zn10	T	Zn	D	2.8	0.0032	0.0006	2524.511	4.8598	13.5822	11.3782	61.9082
T1Zn10	T	Zn	D	2.8	0.0043	0.0008	2558.5	4.7998	13.4058	11.4551	59.0474
T1Zn10	T	Zn	D	2.8	0.0045	0.0008	2493.992	4.9201	13.416	11.3437	58.9301
T1Zn10	T	Zn	D	2.8	0.0046	0.0008	2503.009	4.862	13.4095	11.4127	62.6046
P1control	P	control	0	0	0.7665	0.071	5.312	2.5246	6.4491	0.614	239.7521
P1control	P	control	0	0	1.0949	0.1015	5.495	2.5263	6.4605	0.4982	236.582
P1control	P	control	0	0	0.0834	0.0077	5.129	2.5254	6.4535	0.6491	242.232
P1control	P	control	0	0	0.6483	0.0601	5.821	2.5266	6.6193	0.4907	239.522
P1control	P	control	0	0	0.6483	0.0601	4.803	2.5287	6.6272	0.614	234.1651
P1control	P	control	0	0	0.6684	0.0603	4.79	2.526	6.6193	0.4782	250.2915
P1Cu1/2	P	Cu	A	0.13	0.0072	0.0012	105.185	2.2802	6.999	1.8345	198.6959
P1Cu1/2	P	Cu	A	0.13	0.0232	0.0037	105.344	2.2815	7.009	1.9655	191.5452
P1Cu1/2	P	Cu	A	0.13	0.0218	0.0035	105.026	2.2805	7.002	1.8373	199.2971
P1Cu1/2	P	Cu	A	0.13	0.0174	0.0028	114.648	2.2768	7.0877	1.9855	192.5454
P1Cu1/2	P	Cu	A	0.13	0.0174	0.0028	95.723	2.2795	7.0917	1.8146	177.7457
P1Cu1/2	P	Cu	A	0.13	0.018	0.0029	98.718	2.2785	7.0977	1.9598	209.7805
P1Cu0	P	Cu	B	0.26	0.0021	0.0004	209.657	2.5331	8.3082	4.0443	167.8601
P1Cu0	P	Cu	B	0.26	0.0018	0.0003	210.094	2.5401	8.3191	4.192	167.3202
P1Cu0	P	Cu	B	0.26	0.0037	0.0007	209.22	2.5384	8.3201	4.0755	169.6409
P1Cu0	P	Cu	B	0.26	0.0025	0.0005	222.493	2.5331	8.2704	4.1892	168.2737
P1Cu0	P	Cu	B	0.26	0.0025	0.0005	196.822	2.5414	8.2744	4.0244	154.7084
P1Cu0	P	Cu	B	0.26	0.0026	0.0005	200.663	2.5391	8.2843	4.1863	170.0231
P1Cu2.5	P	Cu	C	0.65	0.0005	0.0001	906.381	2.9896	12.7288	15.9891	68.1203
P1Cu2.5	P	Cu	C	0.65	0.0005	0.0001	906.495	2.9064	12.7324	15.8764	67.1462
P1Cu2.5	P	Cu	C	0.65	0.0007	0.0002	906.267	2.9522	12.7395	15.9754	68.693

P1Cu2.5	P	Cu	C	0.65	0.0006	0.0002	917.04	2.992	12.0346	15.8696	67.9865
P1Cu2.5	P	Cu	C	0.65	0.0006	0.0002	895.722	2.908	12.0418	15.9549	65.3154
P1Cu2.5	P	Cu	C	0.65	0.0006	0.0002	899.161	2.953	12.0382	15.8559	73.2618
P1Cu5	P	Cu	D	1.3	0.0003	0.0001	904.381	4.0227	11.6035	15.3988	56.9371
P1Cu5	P	Cu	D	1.3	0.0004	0.0001	866.495	4.0235	11.6058	15.1511	59.4158
P1Cu5	P	Cu	D	1.3	0.0008	0.0002	906.267	4.0247	11.6139	15.3988	62.9177
P1Cu5	P	Cu	D	1.3	0.0005	0.0001	912.04	4.062	11.6983	15.1544	62.1296
P1Cu5	P	Cu	D	1.3	0.0005	0.0001	885.22	4.0655	11.711	15.3955	55.8487
P1Cu5	P	Cu	D	1.3	0.0005	0.0001	909.137	4.0655	11.7133	15.1214	67.2125
P1control	P	control	0	0	0.7665	0.071	1.94	2.5246	6.4491	0.614	239.7521
P1control	P	control	0	0	1.0949	0.1015	1.96	2.5263	6.4605	0.4982	236.582
P1control	P	control	0	0	0.0834	0.0077	1.92	2.5254	6.4535	0.6491	242.232
P1control	P	control	0	0	0.6483	0.0601	2.129	2.5266	6.6193	0.4907	239.522
P1control	P	control	0	0	0.6483	0.0601	1.751	2.5287	6.6272	0.614	234.1651
P1control	P	control	0	0	0.6684	0.0603	1.794	2.526	6.6193	0.4782	250.2915
P1Pb0	P	Pb	A	0.025	0.2586	0.0325	0.963	1.6213	6.3163	0.9036	238.3974
P1Pb0	P	Pb	A	0.025	0.5483	0.0688	1.078	1.6706	6.3254	0.5876	231.5713
P1Pb0	P	Pb	A	0.025	0.224	0.0281	0.848	1.6496	6.3282	0.8932	242.076
P1Pb0	P	Pb	A	0.025	0.3436	0.0431	0.972	1.6213	6.2413	0.619	237.3482
P1Pb0	P	Pb	A	0.025	0.3436	0.0431	0.955	1.6709	6.245	0.8801	229.0411
P1Pb0	P	Pb	A	0.025	0.3579	0.0448	0.843	1.6514	6.2541	0.5667	242.1317
P1Pb10	P	Pb	B	0.25	0.1081	0.014	46.382	2.704	8.5943	3.803	229.798
P1Pb10	P	Pb	B	0.25	0.1619	0.0209	46.693	2.6798	8.5963	3.904	235.9708
P1Pb10	P	Pb	B	0.25	0.1341	0.0173	46.071	2.6932	8.5992	3.8198	233.1783
P1Pb10	P	Pb	B	0.25	0.1347	0.0174	48.947	2.7053	8.722	3.9096	232.9824
P1Pb10	P	Pb	B	0.25	0.1347	0.0174	43.816	2.6811	8.723	3.7749	213.5638
P1Pb10	P	Pb	B	0.25	0.1363	0.0178	44.36	2.6938	8.7407	3.8844	245.6769
P1Pb50	P	Pb	C	1.25	0.0049	0.001	289.3553	2.6434	8.9189	3.9395	169.2435
P1Pb50	P	Pb	C	1.25	0.013	0.0027	310.139	2.6349	8.9237	4.0921	165.4709
P1Pb50	P	Pb	C	1.25	0.0124	0.0025	260.7688	2.6479	8.937	3.9449	167.8785
P1Pb50	P	Pb	C	1.25	0.0101	0.0021	317.5569	2.645	9.4208	4.0812	172.054

P1Pb50	P	Pb	C	1.25	0.0101	0.0021	269.8601	2.6358	9.4208	3.9204	165.6331
P1Pb50	P	Pb	C	1.25	0.0106	0.0022	278.2293	2.6479	9.438	4.0731	188.6675
P1Pb100	P	Pb	D	2.5	0.0058	0.0014	545.5931	2.7536	9.3025	5.1682	123.0591
P1Pb100	P	Pb	D	2.5	0.0038	0.0009	498.2816	2.7551	9.308	4.7516	110.2242
P1Pb100	P	Pb	D	2.5	0.0013	0.0003	478.0479	2.7566	9.3161	5.1993	111.7552
P1Pb100	P	Pb	D	2.5	0.0036	0.0009	561.6049	2.7612	9.3986	4.9671	115.914
P1Pb100	P	Pb	D	2.5	0.0036	0.0009	424.6352	2.7627	9.4103	5.1605	104.7335
P1Pb100	P	Pb	D	2.5	0.0036	0.0009	466.3875	2.7627	9.4112	4.7516	123.5776
P1control	P	control	0	0	0.7665	0.071	1.934	2.5246	6.4491	0.614	239.7521
P1control	P	control	0	0	1.0949	0.1015	2.019	2.5263	6.4605	0.4982	236.582
P1control	P	control	0	0	0.0834	0.0077	1.849	2.5254	6.4535	0.6491	242.232
P1control	P	control	0	0	0.6483	0.0601	2.183	2.5266	6.6193	0.4907	239.522
P1control	P	control	0	0	0.6483	0.0601	1.684	2.5287	6.6272	0.614	234.1651
P1control	P	control	0	0	0.6684	0.0603	1.682	2.526	6.6193	0.4782	250.2915
P1Cr0	P	Cr	A	0.036	0.8483	0.1029	1.477	1.3589	6.55	0.9167	231.8204
P1Cr0	P	Cr	A	0.036	0.2204	0.0267	1.513	1.4094	6.5603	0.8018	221.0949
P1Cr0	P	Cr	A	0.036	1.0067	0.1221	1.441	1.3882	6.5622	0.9328	235.1955
P1Cr0	P	Cr	A	0.036	0.6918	0.0839	1.592	1.3595	5.8877	0.8446	229.3703
P1Cr0	P	Cr	A	0.036	0.6918	0.0839	1.361	1.4116	5.8915	0.9114	222.3743
P1Cr0	P	Cr	A	0.036	0.695	0.0843	1.364	1.3891	5.9018	0.7938	237.8185
P1Cr5	P	Cr	B	0.18	0.1035	0.0119	4.047	1.5213	6.7884	1.5711	241.6813
P1Cr5	P	Cr	B	0.18	0.1183	0.0136	4.079	1.502	6.7994	1.631	243.329
P1Cr5	P	Cr	B	0.18	0.1332	0.0154	4.015	1.5126	6.8024	1.5768	241.2625
P1Cr5	P	Cr	B	0.18	0.1183	0.0136	4.329	1.523	6.4242	1.6396	242.0909
P1Cr5	P	Cr	B	0.18	0.1183	0.0136	3.765	1.5027	6.4311	1.5711	228.4956
P1Cr5	P	Cr	B	0.18	0.1238	0.0138	3.827	1.5133	6.4451	1.6196	261.0268
P1Cr10	P	Cr	C	0.36	0.0102	0.0012	7.01	1.9358	6.8659	2.3222	219.4719
P1Cr10	P	Cr	C	0.36	0.0111	0.0013	7.052	1.9343	6.8713	2.2014	221.9334
P1Cr10	P	Cr	C	0.36	0.0096	0.0011	6.968	1.937	6.8704	1.5533	222.7087
P1Cr10	P	Cr	C	0.36	0.0103	0.0012	7.628	1.9373	6.1566	1.4607	221.3713
P1Cr10	P	Cr	C	0.36	0.0103	0.0012	6.392	1.9355	6.1602	2.3119	206.3344

P1Cr10	P	Cr	C	0.36	0.0105	0.0012	6.556	1.9379	6.1656	2.1834	231.2349
P1Cr25	P	Cr	D	0.90	0.0114	0.003	127.0827	3.265	7.9127	3.2665	148.3659
P1Cr25	P	Cr	D	0.90	0.0035	0.0009	127.5435	3.3052	7.9165	3.505	151.5294
P1Cr25	P	Cr	D	0.90	0.0125	0.0033	126.6219	3.2679	7.925	3.2773	150.0331
P1Cr25	P	Cr	D	0.90	0.0091	0.0024	139.239	3.2672	8.0797	3.5104	149.9761
P1Cr25	P	Cr	D	0.90	0.0091	0.0024	114.9255	3.3074	8.0835	3.2529	135.0646
P1Cr25	P	Cr	D	0.90	0.0092	0.0025	134.7264	3.2685	8.093	3.486	152.8936
P1control	P	control	0	0	0.7665	0.071	1.538	2.5246	6.4491	0.614	239.7521
P1control	P	control	0	0	1.0949	0.1015	1.548	2.5263	6.4605	0.4982	236.582
P1control	P	control	0	0	0.0834	0.0077	1.528	2.5254	6.4535	0.6491	242.232
P1control	P	control	0	0	0.6483	0.0601	1.551	2.5266	6.6193	0.4907	239.522
P1control	P	control	0	0	0.6483	0.0601	1.525	2.5287	6.6272	0.614	234.1651
P1control	P	control	0	0	0.6684	0.0603	1.519	2.526	6.6193	0.4782	250.2915
P1Ni0	P	Ni	A	0.025	0.1327	0.0157	13.3	2.0491	6.8093	1.0164	232.2986
P1Ni0	P	Ni	A	0.025	0.886	0.1047	13.748	2.0198	6.8123	1.0136	228.0943
P1Ni0	P	Ni	A	0.025	0.2587	0.0306	12.852	2.0349	6.8182	1.0107	233.0319
P1Ni0	P	Ni	A	0.025	0.4258	0.0503	13.563	2.0504	6.2638	1.0333	231.1416
P1Ni0	P	Ni	A	0.025	0.4258	0.0503	13.037	2.0221	6.2727	1.0023	219.4301
P1Ni0	P	Ni	A	0.025	0.4346	0.0506	12.676	2.0375	6.2727	1.0023	242.3184
P1Ni5	P	Ni	B	0.125	0.0557	0.0067	27.618	2.5932	8.0275	3.6185	194.7418
P1Ni5	P	Ni	B	0.125	0.0441	0.0053	28.297	2.5349	8.0344	3.4274	185.4587
P1Ni5	P	Ni	B	0.125	0.0721	0.0087	26.939	2.5677	8.0413	3.6241	202.3148
P1Ni5	P	Ni	B	0.125	0.0573	0.0069	30.135	2.5949	7.9528	3.4331	194.1718
P1Ni5	P	Ni	B	0.125	0.0573	0.0069	25.1	2.5362	7.9538	3.5988	189.0009
P1Ni5	P	Ni	B	0.125	0.0591	0.0072	25.26	2.5687	7.9528	3.4246	201.2726
P1Ni10	P	Ni	C	0.25	0.0144	0.0029	98.177	2.0995	6.7674	1.7302	189.8667
P1Ni10	P	Ni	C	0.25	0.04	0.008	98.467	2.0816	6.774	1.9429	177.7375
P1Ni10	P	Ni	C	0.25	0.0275	0.0055	97.887	2.093	6.774	1.7116	198.7154
P1Ni10	P	Ni	C	0.25	0.0273	0.0055	107.197	2.1005	6.6912	1.9748	188.7732
P1Ni10	P	Ni	C	0.25	0.0273	0.0055	89.157	2.0819	6.7023	1.7063	184.744
P1Ni10	P	Ni	C	0.25	0.0281	0.0055	91.874	2.0943	6.7033	1.9375	196.1309

P1Ni25	P	Ni	D	0.625	0.012	0.0022	123.4709	2.7009	8.9159	5.4683	121.0456
P1Ni25	P	Ni	D	0.625	0.005	0.0009	121.6727	2.7009	8.9266	5.4459	120.6213
P1Ni25	P	Ni	D	0.625	0.0041	0.0008	125.5412	2.7019	8.9266	5.4906	126.6772
P1Ni25	P	Ni	D	0.625	0.007	0.0013	129.0459	2.6957	9.1663	5.4655	122.7813
P1Ni25	P	Ni	D	0.625	0.007	0.0013	118.6393	2.6986	9.1703	5.4627	118.2087
P1Ni25	P	Ni	D	0.625	0.0074	0.0013	118.0021	2.699	9.1693	5.4319	128.1934
P1control	P	control	0	0	0.7665	0.071	0.349	2.5246	6.4491	0.614	239.7521
P1control	P	control	0	0	1.0949	0.1015	0.375	2.5263	6.4605	0.4982	236.582
P1control	P	control	0	0	0.0834	0.0077	0.323	2.5254	6.4535	0.6491	242.232
P1control	P	control	0	0	0.6483	0.0601	0.384	2.5266	6.6193	0.4907	239.522
P1control	P	control	0	0	0.6483	0.0601	0.315	2.5287	6.6272	0.614	234.1651
P1control	P	control	0	0	0.6684	0.0603	0.3	2.526	6.6193	0.4782	250.2915
P1Cd0	P	Cd	A	0.0000945	0.3524	0.0363	1.889	2.4822	7.5578	5.8994	191.0488
P1Cd0	P	Cd	A	0.0000945	0.4253	0.0438	2.007	2.4577	7.5657	5.5942	186.7421
P1Cd0	P	Cd	A	0.0000945	0.2547	0.0263	1.771	2.4708	7.5604	5.9194	193.6149
P1Cd0	P	Cd	A	0.0000945	0.3441	0.0355	1.905	2.4828	7.7023	5.6092	190.4686
P1Cd0	P	Cd	A	0.0000945	0.3441	0.0355	1.873	2.458	7.7067	5.8894	177.5634
P1Cd0	P	Cd	A	0.0000945	0.3504	0.0362	1.761	2.472	7.7058	5.5692	193.5568
P1Cd100	P	Cd	B	0.00945	0.051	0.0049	32.019	2.3759	7.4918	5.6072	188.8358
P1Cd100	P	Cd	B	0.00945	0.1077	0.0102	32.086	2.2888	7.4995	5.9558	170.9738
P1Cd100	P	Cd	B	0.00945	0.0496	0.0047	31.952	2.3349	7.5024	5.5935	199.2853
P1Cd100	P	Cd	B	0.00945	0.0694	0.0066	33.607	2.3766	7.5985	5.9503	186.3649
P1Cd100	P	Cd	B	0.00945	0.0694	0.0066	30.431	2.2895	7.6023	5.588	180.8385
P1Cd100	P	Cd	B	0.00945	0.0712	0.0067	30.894	2.3362	7.611	5.9476	196.4367
P1Cd500	P	Cd	C	0.04725	0.0295	0.0036	114.983	2.1398	7.5222	2.7351	133.2844
P1Cd500	P	Cd	C	0.04725	0.1022	0.0126	115.244	2.2188	7.5275	2.6205	134.1883
P1Cd500	P	Cd	C	0.04725	0.0185	0.0023	114.722	2.1825	7.5405	2.7301	139.2793
P1Cd500	P	Cd	C	0.04725	0.0501	0.0062	128.38	2.1407	7.367	2.613	135.584
P1Cd500	P	Cd	C	0.04725	0.0501	0.0062	101.587	2.2209	7.3697	2.7226	124.7439
P1Cd500	P	Cd	C	0.04725	0.051	0.0063	105.791	2.1831	7.3688	2.608	148.2505
P1Cd1000	P	Cd	D	0.0945	0.0063	0.0017	407.8204	2.5011	9.8052	6.8504	98.2847

P1Cd1000	P	Cd	D	0.0095	0.0054	0.0015	367.8463	2.5906	9.8061	6.683	98.215
P1Cd1000	P	Cd	D	0.0945	0.0018	0.0005	382.8388	2.5981	9.8188	6.8556	104.8978
P1Cd1000	P	Cd	D	0.0945	0.0045	0.0012	294.6212	2.5017	9.6673	6.6727	100.4658
P1Cd1000	P	Cd	D	0.0945	0.0045	0.0012	453.5602	2.5927	9.6763	6.8273	94.7941
P1Cd1000	P	Cd	D	0.0945	0.0047	0.0013	403.063	2.5984	9.6772	6.6572	106.4086
P1control	P	control	0	0	0.7665	0.071	21.082	2.5246	6.4491	0.614	239.7521
P1control	P	control	0	0	1.0949	0.1015	21.979	2.5263	6.4605	0.4982	236.582
P1control	P	control	0	0	0.0834	0.0077	20.185	2.5254	6.4535	0.6491	242.232
P1control	P	control	0	0	0.6483	0.0601	22.878	2.5266	6.6193	0.4907	239.522
P1control	P	control	0	0	0.6483	0.0601	19.287	2.5287	6.6272	0.614	234.1651
P1control	P	control	0	0	0.6684	0.0603	18.988	2.526	6.6193	0.4782	250.2915
P1Zn0	P	Zn	A	0.28	0.0651	0.0097	680.684	0.9534	6.5747	1.5055	211.6799
P1Zn0	P	Zn	A	0.28	0.0447	0.0067	681.202	1.0671	6.5781	1.9097	208.2433
P1Zn0	P	Zn	A	0.28	0.0278	0.0042	680.166	1.0124	6.5859	1.5179	214.9838
P1Zn0	P	Zn	A	0.28	0.0459	0.0069	754.751	0.954	6.3099	1.942	211.6357
P1Zn0	P	Zn	A	0.28	0.0459	0.0069	606.618	1.0683	6.3194	1.4931	196.7124
P1Zn0	P	Zn	A	0.28	0.0474	0.0069	630.788	1.0136	6.3255	1.9097	213.8532
P1Zn2	P	Zn	B	0.56	0.0432	0.006	910.31	2.308	7.4259	3.6529	135.458
P1Zn2	P	Zn	B	0.56	0.0624	0.0087	910.889	2.3695	7.4306	3.0337	126.0093
P1Zn2	P	Zn	B	0.56	0.0584	0.0081	909.731	2.3406	7.4268	3.6502	141.7282
P1Zn2	P	Zn	B	0.56	0.0547	0.0076	947.586	2.3089	7.3203	3.0418	134.3985
P1Zn2	P	Zn	B	0.56	0.0547	0.0076	873.034	2.3707	7.3251	3.6257	125.3193
P1Zn2	P	Zn	B	0.56	0.0554	0.0076	884.881	2.3428	7.3356	3.031	139.2336
P1Zn4	P	Zn	C	1.12	0.0225	0.0071	1109.276	2.3624	8.1609	4.387	122.9137
P1Zn4	P	Zn	C	1.12	0.0472	0.0149	1109.973	2.3474	8.1686	4.2392	122.5672
P1Zn4	P	Zn	C	1.12	0.0168	0.0053	1108.579	2.3576	8.1734	4.3706	125.165
P1Zn4	P	Zn	C	1.12	0.0288	0.0091	1136.819	2.3624	11.0738	4.2693	123.5486
P1Zn4	P	Zn	C	1.12	0.0288	0.0091	1081.734	2.3487	11.0814	4.3623	122.7138
P1Zn4	P	Zn	C	1.12	0.0296	0.0095	1090.217	2.3589	11.0862	4.2255	125.5331
P1Zn10	P	Zn	D	2.8	0.0065	0.0018	2183.094783	6.1826	13.5822	14.2291	58.716
P1Zn10	P	Zn	D	2.8	0.003	0.0009	2185.36087	6.1887	13.585	14.0264	58.1353

P1Zn10	P	Zn	D	2.8	0.0013	0.0004	2180.828696	6.1887	13.5822	14.2621	58.5863
P1Zn10	P	Zn	D	2.8	0.0036	0.001	2201.977826	6.1854	13.4058	14.05	58.4792
P1Zn10	P	Zn	D	2.8	0.0036	0.001	2164.212174	6.192	13.416	14.1867	55.3773
P1Zn10	P	Zn	D	2.8	0.0036	0.0011	2193.417391	6.1925	13.4095	14.017	60.1959
