# Supplementary material for: Production of Antioxidant Molecules in Polygonum aviculare (L.) and Senecio vulgaris (L.) under Metal Stress: A Possible Tool in the Evaluation of Plant Metal Tolerance
Source: Int J Mol Sci. 2020 Oct 3;21(19):7317. doi: 10.3390/ijms21197317 (PMC7582341; doi:10.3390/ijms21197317)
Supplement: Supplementary file 1 [file ijms-21-07317-s001.zip › Supplementary Table S2 dataset Senecio.pdf]

Supplementary Table S2. Complete dataset of *Senecio vulgaris.*

Sample	populat	treatment	factor_conc	metal_conc	fresh_wt	dry_wt	metal_ppm_shoot	flavon	polyp	act_antiox	photo_pigm
B2control	B	control	0	0	1.2467	0.3181	6.617	0.3354	1.0502	0.3889	117.8779
B2control	B	control	0	0	0.8355	0.2132	6.8	0.3245	1.0506	0.3677	116.6123
B2control	B	control	0	0	0.4312	0.11	6.434	0.33	1.0509	0.3879	120.9179
B2control	B	control	0	0	0.8378	0.2138	6.984	0.3361	1.0654	0.3672	118.4694
B2control	B	control	0	0	0.8378	0.2138	6.25	0.3245	1.0654	0.3869	112.566
B2control	B	control	0	0	0.8671	0.2188	6.678	0.3301	1.0685	0.367	126.1357
B2Cu1/2	B	Cu	A	0.13	0.0972	0.018	31.784	0.5824	1.0461	0.2442	115.5976
B2Cu1/2	B	Cu	A	0.13	0.0594	0.011	32.539	0.5871	1.0466	0.1606	115.306
B2Cu1/2	B	Cu	A	0.13	0.0824	0.0153	31.029	0.5861	1.0469	0.2465	116.8968
B2Cu1/2	B	Cu	A	0.13	0.0797	0.0148	33.374	0.5829	1.0188	0.1604	115.9335
B2Cu1/2	B	Cu	A	0.13	0.0797	0.0148	30.194	0.5872	1.0199	0.2436	105.2347
B2Cu1/2	B	Cu	A	0.13	0.0813	0.0152	32.089	0.5868	1.0206	0.1584	117.4066
B2Cu0	B	Cu	B	0.26	0.0523	0.0124	39.169	0.5794	1.1802	0.395	113.9439
B2Cu0	B	Cu	B	0.26	0.0442	0.0105	42.819	0.5619	1.1815	0.3827	115.1558
B2Cu0	B	Cu	B	0.26	0.0427	0.0101	35.519	0.5709	1.1822	0.3981	113.2563
B2Cu0	B	Cu	B	0.26	0.0464	0.011	41.276	0.5795	1.1833	0.3852	114.1187
B2Cu0	B	Cu	B	0.26	0.0464	0.011	37.061	0.5622	1.1848	0.3936	113.1842
B2Cu0	B	Cu	B	0.26	0.0479	0.011	36.923	0.5715	1.1866	0.3808	121.2547
B2Cu2.5	B	Cu	C	0.65	0.0211	0.0047	63.606	0.6046	1.066	0.4077	64.2212
B2Cu2.5	B	Cu	C	0.65	0.0305	0.0068	59.748	0.5909	1.0671	0.3921	65.1966
B2Cu2.5	B	Cu	C	0.65	0.0374	0.0084	47.464	0.5833	1.0674	0.4111	65.4331
B2Cu2.5	B	Cu	C	0.65	0.0297	0.0066	65.318	0.6053	1.0655	0.391	64.9503
B2Cu2.5	B	Cu	C	0.65	0.0297	0.0066	61.894	0.5915	1.0655	0.4077	61.071
B2Cu2.5	B	Cu	C	0.65	0.0312	0.0068	58.605	0.5834	1.0677	0.39	66.2302
B2Cu5	B	Cu	D	1.3	0.0021	0.001	411.001	0.9549	1.4642	0.5917	52.0942
B2Cu5	B	Cu	D	1.3	0.0029	0.0014	445.421	0.9692	1.4652	0.6065	61.2375
B2Cu5	B	Cu	D	1.3	0.0233	0.011	376.58	0.9622	1.4664	0.5928	60.019
B2Cu5	B	Cu	D	1.3	0.0094	0.0044	419.749	0.9555	1.4417	0.6044	61.4701

B2Cu5	B	Cu	D	1.3	0.0094	0.0045	402.252	0.9697	1.4433	0.5897	58.9301
B2Cu5	B	Cu	D	1.3	0.0097	0.0045	382.413	0.9629	1.4419	0.6044	62.945
B2control	B	control	0	0	1.2467	0.3181	0.297	0.3354	1.0502	0.3889	117.8779
B2control	B	control	0	0	0.8355	0.2132	0.317	0.3245	1.0506	0.3677	116.6123
B2control	B	control	0	0	0.4312	0.11	0.277	0.33	1.0509	0.3879	120.9179
B2control	B	control	0	0	0.8378	0.2138	0.335	0.3361	1.0654	0.3672	118.4694
B2control	B	control	0	0	0.8378	0.2138	0.258	0.3245	1.0654	0.3869	112.566
B2control	B	control	0	0	0.8671	0.2188	0.302	0.3301	1.0685	0.367	126.1357
B2Pb0	B	Pb	A	0.025	0.2082	0.0444	7.005	0.1806	1.0526	0.3323	126.3889
B2Pb0	B	Pb	A	0.025	0.3389	0.0723	8.247	0.2232	1.054	0.324	124.1155
B2Pb0	B	Pb	A	0.025	0.3426	0.0731	5.762	0.2288	1.0544	0.3337	129.2644
B2Pb0	B	Pb	A	0.025	0.2966	0.0633	7.308	0.1807	1.0392	0.3268	126.5896
B2Pb0	B	Pb	A	0.025	0.2966	0.0633	6.701	0.2236	1.0395	0.3305	117.772
B2Pb0	B	Pb	A	0.025	0.3097	0.0656	5.965	0.2292	1.0398	0.3221	127.694
B2Pb10	B	Pb	B	0.25	0.0617	0.015	372.446	0.273	1.0702	0.32	145.0207
B2Pb10	B	Pb	B	0.25	0.1512	0.0368	402.383	0.2745	1.0712	0.2925	143.6079
B2Pb10	B	Pb	B	0.25	0.0441	0.0107	342.508	0.2741	1.0717	0.3177	147.182
B2Pb10	B	Pb	B	0.25	0.0857	0.0208	381.739	0.2733	1.0609	0.2914	145.2702
B2Pb10	B	Pb	B	0.25	0.0857	0.0208	363.153	0.2748	1.0617	0.3177	131.9969
B2Pb10	B	Pb	B	0.25	0.089	0.0215	348.704	0.2744	1.0633	0.2909	148.9507
B2Pb50	B	Pb	C	1.25	0.0276	0.0069	762.901	0.9149	1.5357	0.0871	55.3173
B2Pb50	B	Pb	C	1.25	0.0061	0.0015	895.108	0.9521	1.5357	0.0948	55.6364
B2Pb50	B	Pb	C	1.25	0.0088	0.0022	630.693	0.9782	1.5391	0.0876	57.5341
B2Pb50	B	Pb	C	1.25	0.0142	0.0035	799.195	0.9154	1.5036	0.0976	56.1626
B2Pb50	B	Pb	C	1.25	0.0142	0.0035	726.606	0.9529	1.5067	0.0861	55.6887
B2Pb50	B	Pb	C	1.25	0.0144	0.0036	654.889	0.9789	1.5053	0.0924	60.4464
B2Pb100	B	Pb	D	2.5	0.0089	0.0029	3082.151	0.8957	1.3804	0.0985	65.9135
B2Pb100	B	Pb	D	2.5	0.0126	0.0042	3362.33	0.8754	1.3818	0.1129	67.4385
B2Pb100	B	Pb	D	2.5	0.0152	0.005	2801.972	0.8873	1.3848	0.1021	70.5858
B2Pb100	B	Pb	D	2.5	0.0122	0.004	3155.325	0.8962	1.401	0.1144	67.9793
B2Pb100	B	Pb	D	2.5	0.0122	0.004	3008.976	0.8762	1.4029	0.0974	61.4841

B2Pb100	B	Pb	D	2.5	0.0128	0.0042	2850.755	0.8883	1.4013	0.1129	70.4807
B2control	B	control	0	0	1.2467	0.3181	8.042	0.3354	1.0502	0.3889	117.8779
B2control	B	control	0	0	0.8355	0.2132	8.127	0.3245	1.0506	0.3677	116.6123
B2control	B	control	0	0	0.4312	0.11	7.957	0.33	1.0509	0.3879	120.9179
B2control	B	control	0	0	0.8378	0.2138	8.612	0.3361	1.0654	0.3672	118.4694
B2control	B	control	0	0	0.8378	0.2138	7.472	0.3245	1.0654	0.3869	112.566
B2control	B	control	0	0	0.8671	0.2188	8.337	0.3301	1.0685	0.367	126.1357
B2Cr0	B	Cr	A	0.036	0.4346	0.1037	1.456	0.3115	1.012	0.3725	170.6848
B2Cr0	B	Cr	A	0.036	0.2975	0.071	2.109	0.3601	1.0129	0.3494	173.8281
B2Cr0	B	Cr	A	0.036	0.5782	0.138	1.804	0.3445	1.0129	0.3756	169.8687
B2Cr0	B	Cr	A	0.036	0.4368	0.1042	1.59	0.3122	1.0027	0.3522	171.4605
B2Cr0	B	Cr	A	0.036	0.4368	0.1042	1.522	0.3604	1.0038	0.3714	159.1687
B2Cr0	B	Cr	A	0.036	0.4463	0.1089	1.893	0.345	1.0032	0.3491	186.4534
B2Cr5	B	Cr	B	0.18	0.2055	0.0495	211.852	0.4312	1.0499	0.3314	120.5663
B2Cr5	B	Cr	B	0.18	0.137	0.033	231.451	0.4337	1.0517	0.3245	121.6125
B2Cr5	B	Cr	B	0.18	0.4251	0.1024	192.252	0.4333	1.0517	0.3322	124.6411
B2Cr5	B	Cr	B	0.18	0.2559	0.0616	232.669	0.4312	1.0667	0.3242	122.2733
B2Cr5	B	Cr	B	0.18	0.2559	0.0616	191.034	0.4338	1.0671	0.3289	112.2911
B2Cr5	B	Cr	B	0.18	0.2657	0.0645	206.131	0.4335	1.0679	0.3223	125.09
B2Cr10	B	Cr	C	0.36	0.0549	0.0115	810.851	0.5771	1.1463	0.3676	122.9956
B2Cr10	B	Cr	C	0.36	0.0853	0.0179	830.45	0.5864	1.1475	0.3007	125.8638
B2Cr10	B	Cr	C	0.36	0.121	0.0254	791.252	0.5826	1.1463	0.3702	119.9697
B2Cr10	B	Cr	C	0.36	0.0871	0.0183	828.5	0.5771	1.1285	0.3007	122.9431
B2Cr10	B	Cr	C	0.36	0.0871	0.0183	793.202	0.5866	1.1296	0.3668	114.3073
B2Cr10	B	Cr	C	0.36	0.0906	0.019	803.018	0.5831	1.1296	0.2993	131.1531
B2Cr25	B	Cr	D	0.9	0.0941	0.018	861.471	0.87	1.6083	0.4264	82.4404
B2Cr25	B	Cr	D	0.9	0.0322	0.0062	934.918	0.8284	1.6092	0.3451	80.9327
B2Cr25	B	Cr	D	0.9	0.038	0.0073	788.024	0.8121	1.612	0.4275	83.1595
B2Cr25	B	Cr	D	0.9	0.0548	0.0105	929.705	0.8703	1.6152	0.3518	82.1775
B2Cr25	B	Cr	D	0.9	0.0548	0.0105	793.237	0.8293	1.6163	0.4241	82.119
B2Cr25	B	Cr	D	0.9	0.0562	0.011	833.513	0.8127	1.6165	0.3451	89.0919

B2control	B	control	0	0	1.2467	0.3181	1.587	0.3354	1.0502	0.3889	117.8779
B2control	B	control	0	0	0.8355	0.2132	1.597	0.3245	1.0506	0.3677	116.6123
B2control	B	control	0	0	0.4312	0.11	1.577	0.33	1.0509	0.3879	120.9179
B2control	B	control	0	0	0.8378	0.2138	1.6	0.3361	1.0654	0.3672	118.4694
B2control	B	control	0	0	0.8378	0.2138	1.575	0.3245	1.0654	0.3869	112.566
B2control	B	control	0	0	0.8671	0.2188	1.586	0.3301	1.0685	0.367	126.1357
B2Ni0	B	Ni	A	0.025	0.822	0.1838	21.497	0.3481	1.0087	0.3256	115.0172
B2Ni0	B	Ni	A	0.025	0.4522	0.1011	18.874	0.3493	1.0101	0.3245	114.5151
B2Ni0	B	Ni	A	0.025	0.9052	0.2024	17.12	0.3493	1.0098	0.3261	118.5033
B2Ni0	B	Ni	A	0.025	0.7265	0.1625	22.949	0.3482	0.9859	0.3235	116.0119
B2Ni0	B	Ni	A	0.025	0.7265	0.1625	20.045	0.3498	0.9861	0.3247	114.994
B2Ni0	B	Ni	A	0.025	0.7294	0.1666	17.088	0.3495	0.9861	0.3226	118.0939
B2Ni5	B	Ni	B	0.125	0.8638	0.2303	70.789	0.3207	1.0761	0.3356	125.8911
B2Ni5	B	Ni	B	0.125	1.0259	0.2735	81.298	0.2466	1.0764	0.3197	130.5946
B2Ni5	B	Ni	B	0.125	0.7485	0.1995	66.28	0.2839	1.0769	0.3392	127.1433
B2Ni5	B	Ni	B	0.125	0.8794	0.2344	71.679	0.3208	1.0711	0.3225	127.8764
B2Ni5	B	Ni	B	0.125	0.8794	0.2344	69.9	0.2471	1.0719	0.3353	121.3097
B2Ni5	B	Ni	B	0.125	0.884	0.2345	56.874	0.284	1.0712	0.3189	139.6396
B2Ni10	B	Ni	C	0.25	0.2874	0.0736	130.996	0.3524	1.053	0.2664	44.4146
B2Ni10	B	Ni	C	0.25	0.7924	0.203	146.316	0.3435	1.0543	0.2971	46.0563
B2Ni10	B	Ni	C	0.25	0.2241	0.0574	115.676	0.349	1.0559	0.2667	49.1032
B2Ni10	B	Ni	C	0.25	0.4346	0.1113	131.732	0.3529	1.0646	0.2974	46.5247
B2Ni10	B	Ni	C	0.25	0.4346	0.1113	130.26	0.3437	1.0646	0.2656	42.6946
B2Ni10	B	Ni	C	0.25	0.4557	0.1154	116.167	0.3497	1.0676	0.2949	48.1457
B2Ni25	B	Ni	D	0.625	0.2261	0.0672	187.3243	0.5053	1.3711	0.5314	50.788
B2Ni25	B	Ni	D	0.625	0.1028	0.0305	209.2319	0.5328	1.3728	0.5112	47.0033
B2Ni25	B	Ni	D	0.625	0.1043	0.031	165.4167	0.4888	1.3743	0.5302	51.2206
B2Ni25	B	Ni	D	0.625	0.1444	0.0429	188.3768	0.5058	1.3786	0.5089	52.7554
B2Ni25	B	Ni	D	0.625	0.1444	0.0429	186.2718	0.533	1.3801	0.5294	50.1577
B2Ni25	B	Ni	D	0.625	0.1512	0.0438	166.1188	0.4889	1.3791	0.5089	51.3442
B2control	B	control	0	0	1.2467	0.3181	0.374	0.3354	1.0502	0.3889	117.8779

B2control	B	control	0	0	0.8355	0.2132	0.4	0.3245	1.0506	0.3677	116.6123
B2control	B	control	0	0	0.4312	0.11	0.348	0.33	1.0509	0.3879	120.9179
B2control	B	control	0	0	0.8378	0.2138	0.394	0.3361	1.0654	0.3672	118.4694
B2control	B	control	0	0	0.8378	0.2138	0.355	0.3245	1.0654	0.3869	112.566
B2control	B	control	0	0	0.8671	0.2188	0.328	0.3301	1.0685	0.367	126.1357
B2Cd0	B	Cd	A	0.0000945	0.7619	0.1471	2.113	0.2376	0.9831	0.3087	137.977
B2Cd0	B	Cd	A	0.0000945	0.4654	0.0898	2.603	0.2939	0.9845	0.311	136.6569
B2Cd0	B	Cd	A	0.0000945	0.4807	0.0928	2.024	0.2661	0.9845	0.3125	140.8853
B2Cd0	B	Cd	A	0.0000945	0.5693	0.1099	2.222	0.2382	0.959	0.3095	138.5064
B2Cd0	B	Cd	A	0.0000945	0.5693	0.1099	2.005	0.2942	0.9602	0.3077	124.8559
B2Cd0	B	Cd	A	0.0000945	0.5744	0.1138	2.097	0.2666	0.9611	0.3087	146.7143
B2Cd100	B	Cd	B	0.00945	0.2264	0.065	255.787	0.325	0.8736	0.2285	116.7785
B2Cd100	B	Cd	B	0.00945	0.2728	0.0783	255.361	0.331	0.8739	0.2085	117.5334
B2Cd100	B	Cd	B	0.00945	0.1504	0.0432	246.213	0.328	0.8742	0.2292	119.0247
B2Cd100	B	Cd	B	0.00945	0.2165	0.0621	271.38	0.3257	0.8736	0.2093	117.7789
B2Cd100	B	Cd	B	0.00945	0.2165	0.0622	240.195	0.3312	0.8742	0.2262	115.2911
B2Cd100	B	Cd	B	0.00945	0.2237	0.0652	246.609	0.3281	0.8741	0.2065	124.6508
B2Cd500	B	Cd	C	0.04725	0.1353	0.0346	645.75	0.4432	0.9012	0.1427	51.9126
B2Cd500	B	Cd	C	0.04725	0.2072	0.053	647.593	0.4001	0.9017	0.167	52.2894
B2Cd500	B	Cd	C	0.04725	0.1777	0.0455	643.907	0.3778	0.902	0.1453	55.2906
B2Cd500	B	Cd	C	0.04725	0.1734	0.0444	672.233	0.4439	0.9249	0.1673	53.1642
B2Cd500	B	Cd	C	0.04725	0.1734	0.0444	619.267	0.4006	0.9264	0.1407	52.7331
B2Cd500	B	Cd	C	0.04725	0.18	0.0465	626.252	0.3778	0.9264	0.1665	55.3535
B2Cd1000	B	Cd	D	0.0945	0.1122	0.0266	709.647	1.4142	1.3629	0.2323	69.5738
B2Cd1000	B	Cd	D	0.0945	0.09	0.0214	659.107	1.3208	1.3645	0.2205	70.7655
B2Cd1000	B	Cd	D	0.0945	0.0977	0.0232	760.187	1.3208	1.3652	0.2358	72.5523
B2Cd1000	B	Cd	D	0.0945	0.1	0.0237	713.18	1.4144	1.3512	0.2213	70.9639
B2Cd1000	B	Cd	D	0.0945	0.1	0.0237	706.114	1.3215	1.3517	0.2319	70.1433
B2Cd1000	B	Cd	D	0.0945	0.1011	0.0241	657.831	1.3217	1.3553	0.2189	72.2157
B2control	B	control	0	0	1.2467	0.3181	38.87	0.3354	1.0502	0.3889	117.8779
B2control	B	control	0	0	0.8355	0.2132	39.767	0.3245	1.0506	0.3677	116.6123

B2control	B	control	0	0	0.4312	0.11	37.973	0.33	1.0509	0.3879	120.9179
B2control	B	control	0	0	0.8378	0.2138	39.283	0.3361	1.0654	0.3672	118.4694
B2control	B	control	0	0	0.8378	0.2138	38.456	0.3245	1.0654	0.3869	112.566
B2control	B	control	0	0	0.8671	0.2188	37.697	0.3301	1.0685	0.367	126.1357
B2Zn0	B	Zn	A	0.28	0.2192	0.0226	543.051	0.3287	1.0318	0.3058	126.542
B2Zn0	B	Zn	A	0.28	0.4001	0.0413	544.816	0.3267	1.032	0.3259	120.9752
B2Zn0	B	Zn	A	0.28	0.1503	0.0155	541.286	0.3282	1.0339	0.3096	132.6186
B2Zn0	B	Zn	A	0.28	0.2565	0.0265	565.151	0.329	1.0635	0.3248	126.712
B2Zn0	B	Zn	A	0.28	0.2565	0.0265	520.952	0.3274	1.065	0.3055	124.5271
B2Zn0	B	Zn	A	0.28	0.2569	0.0272	526.553	0.3286	1.065	0.3235	138.8013
B2Zn2	B	Zn	B	0.56	0.4413	0.1175	1481.21	0.335	1.0192	0.3478	88.1376
B2Zn2	B	Zn	B	0.56	0.2491	0.0663	1569.176	0.3246	1.0195	0.3412	87.9164
B2Zn2	B	Zn	B	0.56	0.4011	0.1068	1393.245	0.3233	1.02	0.3481	92.1762
B2Zn2	B	Zn	B	0.56	0.3638	0.0968	1504.489	0.3351	1.0353	0.3435	89.4101
B2Zn2	B	Zn	B	0.56	0.3638	0.0969	1457.932	0.3248	1.0364	0.3465	83.1558
B2Zn2	B	Zn	B	0.56	0.3811	0.1003	1377.726	0.3237	1.0361	0.3407	91.2912
B2Zn4	B	Zn	C	1.12	0.1965	0.0451	1626.366	0.6378	1.2848	0.4628	51.0465
B2Zn4	B	Zn	C	1.12	0.0525	0.012	1627.333	0.6335	1.2863	0.4225	49.498
B2Zn4	B	Zn	C	1.12	0.1706	0.0391	1625.399	0.6357	1.2868	0.463	57.7216
B2Zn4	B	Zn	C	1.12	0.1399	0.0321	1655.272	0.6383	1.2574	0.4222	52.7554
B2Zn4	B	Zn	C	1.12	0.1399	0.0321	1597.46	0.6338	1.2578	0.462	50.3715
B2Zn4	B	Zn	C	1.12	0.144	0.0326	1606.128	0.6361	1.2586	0.4219	56.213
B2Zn10	B	Zn	D	2.8	0.0179	0.0056	1821.53	1.1252	1.6171	0.6345	48.7013
B2Zn10	B	Zn	D	2.8	0.0127	0.004	1822.613	1.1252	1.6171	0.6327	48.1396
B2Zn10	B	Zn	D	2.8	0.0765	0.0239	1820.447	1.0809	1.6206	0.6336	52.9416
B2Zn10	B	Zn	D	2.8	0.0357	0.0111	1853.905	1.126	1.6314	0.6351	44.8421
B2Zn10	B	Zn	D	2.8	0.0357	0.0112	1789.155	1.1259	1.6321	0.6324	48.6687
B2Zn10	B	Zn	D	2.8	0.0362	0.0113	1798.863	1.0815	1.6325	0.6324	52.3469
M2control	M	control	0	0	0.5598	0.115	11.742	1.1876	1.0046	0.7149	135.0545
M2control	M	control	0	0	0.347	0.0713	12.005	1.1489	1.005	0.7797	142.9999
M2control	M	control	0	0	0.7495	0.154	11.479	1.1684	1.0076	0.7139	139.4581

M2control	M	control	0	0	0.5521	0.1134	12.044	1.1894	0.9647	0.7807	139.1708
M2control	M	control	0	0	0.5521	0.1134	11.44	1.1498	0.9661	0.7131	134.9877
M2control	M	control	0	0	0.5674	0.1191	11.68	1.1705	0.9649	0.7786	150.4054
M2Cu1/2	M	Cu	A	0.13	0.04	0.0102	36.231	1.9745	1.0221	1.2184	114.786
M2Cu1/2	M	Cu	A	0.13	0.0872	0.0222	33.91	1.9903	1.0228	1.2316	118.381
M2Cu1/2	M	Cu	A	0.13	0.027	0.0069	28.552	1.9869	1.0228	1.2207	119.134
M2Cu1/2	M	Cu	A	0.13	0.0514	0.0131	37.726	1.9752	1.1193	1.2316	117.4336
M2Cu1/2	M	Cu	A	0.13	0.0514	0.0131	34.736	1.9906	1.1201	1.2164	108.4079
M2Cu1/2	M	Cu	A	0.13	0.0533	0.0135	29.548	1.9876	1.1196	1.2296	121.4728
M2Cu0	M	Cu	B	0.26	0.0355	0.0152	40.293	1.5283	0.9387	1.265	112.1175
M2Cu0	M	Cu	B	0.26	0.0713	0.0306	40.843	1.4823	0.9401	1.2457	110.697
M2Cu0	M	Cu	B	0.26	0.019	0.0082	39.743	1.5061	0.9401	1.2652	119.1976
M2Cu0	M	Cu	B	0.26	0.0419	0.018	43.272	1.5294	1.004	1.2453	114.004
M2Cu0	M	Cu	B	0.26	0.0419	0.018	37.315	1.4828	1.0047	1.263	112.7611
M2Cu0	M	Cu	B	0.26	0.0419	0.0182	41.729	1.5066	1.0043	1.2446	122.0143
M2Cu2.5	M	Cu	C	0.65	0.031	0.0108	227.152	1.5251	1.1828	1.2931	106.1595
M2Cu2.5	M	Cu	C	0.65	0.0468	0.0164	227.29	1.4843	1.183	1.2875	107.9992
M2Cu2.5	M	Cu	C	0.65	0.0333	0.0116	227.014	1.505	1.1853	1.2958	112.5681
M2Cu2.5	M	Cu	C	0.65	0.037	0.0129	239.116	1.5251	1.2574	1.2859	108.9089
M2Cu2.5	M	Cu	C	0.65	0.037	0.0129	215.188	1.4859	1.2577	1.2922	105.6205
M2Cu2.5	M	Cu	C	0.65	0.0388	0.0133	234.99	1.5073	1.2575	1.2853	112.909
M2Cu5	M	Cu	D	1.3	0.0107	0.0038	246.8826	3.0667	1.4686	1.6275	56.0942
M2Cu5	M	Cu	D	1.3	0.0085	0.003	259.6134	3.1126	1.4696	1.618	51.2375
M2Cu5	M	Cu	D	1.3	0.0073	0.0026	234.5168	3.0902	1.471	1.6311	51.019
M2Cu5	M	Cu	D	1.3	0.0088	0.0031	223.6323	3.0674	1.484	1.62	54.4701
M2Cu5	M	Cu	D	1.3	0.0088	0.0031	247.9354	3.1139	1.4854	1.6272	58.9301
M2Cu5	M	Cu	D	1.3	0.0088	0.0031	257.7832	3.0911	1.4863	1.6166	52.945
M2control	M	control	0	0	0.5598	0.115	1.365	1.1876	1.0046	0.7149	135.0545
M2control	M	control	0	0	0.347	0.0713	1.378	1.1489	1.005	0.7797	142.9999
M2control	M	control	0	0	0.7495	0.154	1.352	1.1684	1.0076	0.7139	139.4581
M2control	M	control	0	0	0.5521	0.1134	1.582	1.1894	0.9647	0.7807	139.1708

M2control	M	control	0	0	0.5521	0.1134	1.148	1.1498	0.9661	0.7131	134.9877
M2control	M	control	0	0	0.5674	0.1191	1.496	1.1705	0.9649	0.7786	150.4054
M2Pb0	M	Pb	A	0.025	0.5419	0.1657	7.049	0.9353	1.259	0.558	153.0549
M2Pb0	M	Pb	A	0.025	0.4703	0.1438	7.07	0.9403	1.2604	0.493	152.2131
M2Pb0	M	Pb	A	0.025	0.6618	0.2023	7.028	0.9391	1.2607	0.5615	154.7441
M2Pb0	M	Pb	A	0.025	0.558	0.1706	7.588	0.9366	1.3094	0.4973	153.3374
M2Pb0	M	Pb	A	0.025	0.558	0.1706	6.51	0.9416	1.3113	0.5567	151.458
M2Pb0	M	Pb	A	0.025	0.5661	0.1761	7.387	0.9403	1.3105	0.493	159.9701
M2Pb10	M	Pb	B	0.25	0.0496	0.0135	254.204	1.0563	1.4594	0.5207	123.1093
M2Pb10	M	Pb	B	0.25	0.0399	0.0108	254.349	1.0019	1.4609	0.5398	129.8722
M2Pb10	M	Pb	B	0.25	0.1075	0.0292	254.059	1.0294	1.4625	0.5227	122.5421
M2Pb10	M	Pb	B	0.25	0.0657	0.0178	254.874	1.0585	1.4913	0.5398	125.1746
M2Pb10	M	Pb	B	0.25	0.0657	0.0178	253.535	1.0032	1.4914	0.5185	116.6946
M2Pb10	M	Pb	B	0.25	0.0679	0.0181	254.506	1.031	1.4918	0.5387	136.7051
M2Pb50	M	Pb	C	1.25	0.0514	0.0164	3714.052	0.826	1.7721	0.6474	49.3994
M2Pb50	M	Pb	C	1.25	0.0291	0.0093	3714.146	0.7789	1.7723	0.6234	50.8998
M2Pb50	M	Pb	C	1.25	0.0135	0.0043	3713.958	0.8038	1.7745	0.6507	54.5563
M2Pb50	M	Pb	C	1.25	0.0313	0.01	3714.28	0.8263	1.8092	0.6244	51.6185
M2Pb50	M	Pb	C	1.25	0.0313	0.01	3713.823	0.7813	1.8102	0.6457	46.7063
M2Pb50	M	Pb	C	1.25	0.0324	0.0105	3714.11	0.8054	1.8094	0.6207	55.0058
M2Pb100	M	Pb	D	2.5	0.0052	0.002	6277.83	1.0656	2.2611	0.9483	61.2887
M2Pb100	M	Pb	D	2.5	0.0156	0.0059	6277.842	1.0779	2.2618	0.931	63.3051
M2Pb100	M	Pb	D	2.5	0.015	0.0057	6277.819	1.0766	2.2641	0.9519	67.9739
M2Pb100	M	Pb	D	2.5	0.0119	0.0045	6278.915	1.0679	2.2487	0.9306	64.1892
M2Pb100	M	Pb	D	2.5	0.0119	0.0045	6276.746	1.0816	2.251	0.9479	58.7183
M2Pb100	M	Pb	D	2.5	0.0121	0.0046	6278.542	1.0793	2.2515	0.9283	66.8893
M2control	M	control	0	0	0.5598	0.115	3.316	1.1876	1.0046	0.7149	135.0545
M2control	M	control	0	0	0.347	0.0713	3.454	1.1489	1.005	0.7797	142.9999
M2control	M	control	0	0	0.7495	0.154	3.178	1.1684	1.0076	0.7139	139.4581
M2control	M	control	0	0	0.5521	0.1134	3.592	1.1894	0.9647	0.7807	139.1708
M2control	M	control	0	0	0.5521	0.1134	3.04	1.1498	0.9661	0.7131	134.9877

M2control	M	control	0	0	0.5674	0.1191	3.362	1.1705	0.9649	0.7786	150.4054
M2Cr0	M	Cr	A	0.036	0.3327	0.0806	9.69	0.9249	1.2166	0.8195	150.9389
M2Cr0	M	Cr	A	0.036	0.2745	0.0665	9.801	0.9268	1.2181	0.8548	148.857
M2Cr0	M	Cr	A	0.036	0.0589	0.0143	9.579	0.9286	1.2186	0.8219	153.3179
M2Cr0	M	Cr	A	0.036	0.222	0.0538	9.94	0.9255	1.1929	0.8563	151.0379
M2Cr0	M	Cr	A	0.036	0.222	0.0538	9.44	0.9271	1.1931	0.8182	148.5722
M2Cr0	M	Cr	A	0.036	0.2291	0.0562	9.746	0.9302	1.1942	0.8537	152.259
M2Cr5	M	Cr	B	0.18	0.2488	0.0615	4.666	1.3905	1.2801	1.0603	162.0858
M2Cr5	M	Cr	B	0.18	0.6496	0.1606	4.701	1.3986	1.2811	1.0672	159.9719
M2Cr5	M	Cr	B	0.18	0.3026	0.0748	4.631	1.3973	1.2833	1.06	166.1064
M2Cr5	M	Cr	B	0.18	0.4003	0.099	4.759	1.3905	1.359	1.0683	162.7214
M2Cr5	M	Cr	B	0.18	0.4003	0.099	4.573	1.3989	1.3591	1.059	151.7309
M2Cr5	M	Cr	B	0.18	0.4027	0.1029	4.693	1.3986	1.3604	1.0648	167.1296
M2Cr10	M	Cr	C	0.36	0.0417	0.0093	52.854	1.8605	1.5441	1.4489	143.8254
M2Cr10	M	Cr	C	0.36	0.0874	0.0196	53.002	1.8906	1.5449	1.3367	144.3867
M2Cr10	M	Cr	C	0.36	0.0633	0.0142	52.706	1.8784	1.5472	1.4522	147.5503
M2Cr10	M	Cr	C	0.36	0.0641	0.0144	53.812	1.8615	1.5813	1.3414	145.2541
M2Cr10	M	Cr	C	0.36	0.0641	0.0144	51.895	1.8922	1.5821	1.4478	137.5065
M2Cr10	M	Cr	C	0.36	0.0661	0.0145	53.345	1.8797	1.5846	1.3359	152.0525
M2Cr25	M	Cr	D	0.9	0.0819	0.0175	1036.215	2.2682	1.9577	1.9665	114.6427
M2Cr25	M	Cr	D	0.9	0.0539	0.0115	1036.43	2.3632	1.9587	1.9582	114.9299
M2Cr25	M	Cr	D	0.9	0.0226	0.0048	1036	2.3166	1.9577	1.9649	117.67
M2Cr25	M	Cr	D	0.9	0.0528	0.0113	1037.932	2.2686	2.0306	1.9579	115.7475
M2Cr25	M	Cr	D	0.9	0.0528	0.0113	1034.499	2.3643	2.0327	1.9649	111.1191
M2Cr25	M	Cr	D	0.9	0.0547	0.0117	1037.145	2.3177	2.0329	1.9552	120.6306
M2control	M	control	0	0	0.5598	0.115	3.21	1.1876	1.0046	0.7149	135.0545
M2control	M	control	0	0	0.347	0.0713	3.351	1.1489	1.005	0.7797	142.9999
M2control	M	control	0	0	0.7495	0.154	3.069	1.1684	1.0076	0.7139	139.4581
M2control	M	control	0	0	0.5521	0.1134	3.337	1.1894	0.9647	0.7807	139.1708
M2control	M	control	0	0	0.5521	0.1134	3.084	1.1498	0.9661	0.7131	134.9877
M2control	M	control	0	0	0.5674	0.1191	3.154	1.1705	0.9649	0.7786	150.4054

M2Ni0	M	Ni	A	0.025	0.7326	0.149	49.559	1.3343	1.3144	0.8656	125.6281
M2Ni0	M	Ni	A	0.025	0.3725	0.0758	50.463	1.3391	1.315	0.8366	132.3514
M2Ni0	M	Ni	A	0.025	0.4254	0.0865	48.655	1.3391	1.3145	0.8684	123.9587
M2Ni0	M	Ni	A	0.025	0.5102	0.1038	51.148	1.3352	1.326	0.8377	127.3127
M2Ni0	M	Ni	A	0.025	0.5102	0.1038	47.971	1.3397	1.326	0.8645	115.8402
M2Ni0	M	Ni	A	0.025	0.5321	0.104	49.714	1.3404	1.3271	0.8347	134.0832
M2Ni5	M	Ni	B	0.125	0.3279	0.0652	231.653	1.0652	1.5923	0.5236	94.733
M2Ni5	M	Ni	B	0.125	0.9036	0.1798	232.092	0.8189	1.5935	0.5138	96.5933
M2Ni5	M	Ni	B	0.125	0.2102	0.0418	231.214	0.9429	1.5943	0.5216	100.0679
M2Ni5	M	Ni	B	0.125	0.4806	0.0956	234.023	1.0656	1.4739	0.5124	97.1314
M2Ni5	M	Ni	B	0.125	0.4806	0.0956	229.283	0.8202	1.4757	0.5208	95.3513
M2Ni5	M	Ni	B	0.125	0.4889	0.0997	232.794	0.9445	1.4766	0.5111	106.4609
M2Ni10	M	Ni	C	0.25	0.4558	0.1128	523.4082	1.2083	1.8862	0.7805	70.2171
M2Ni10	M	Ni	C	0.25	0.4676	0.1157	523.578	1.1776	1.8874	0.7564	72.0584
M2Ni10	M	Ni	C	0.25	0.3679	0.0911	523.2383	1.1965	1.8879	0.7833	74.1622
M2Ni10	M	Ni	C	0.25	0.4304	0.1065	533.2668	1.2089	1.8671	0.7586	72.1459
M2Ni10	M	Ni	C	0.25	0.4304	0.1065	513.5495	1.1786	1.8675	0.7788	69.8119
M2Ni10	M	Ni	C	0.25	0.4465	0.1114	529.811	1.1988	1.8676	0.7553	75.165
M2Ni25	M	Ni	D	0.625	0.5118	0.1287	532.5633	1.4871	2.051	1.4004	41.3961
M2Ni25	M	Ni	D	0.625	0.1365	0.0343	532.5289	1.4875	2.051	1.4178	45.9043
M2Ni25	M	Ni	D	0.625	0.1513	0.0381	528.3633	1.4878	2.0524	1.4013	45.0004
M2Ni25	M	Ni	D	0.625	0.2665	0.067	540.2357	1.5032	2.057	1.4169	48.419
M2Ni25	M	Ni	D	0.625	0.2665	0.067	519.9161	1.5039	2.0578	1.4001	41.3684
M2Ni25	M	Ni	D	0.625	0.2722	0.0673	538.4257	1.5068	2.0581	1.4166	49.1367
M2control	M	control	0	0	0.5598	0.115	1.317	1.1876	1.0046	0.7149	135.0545
M2control	M	control	0	0	0.347	0.0713	1.633	1.1489	1.005	0.7797	142.9999
M2control	M	control	0	0	0.7495	0.154	1.7	1.1684	1.0076	0.7139	139.4581
M2control	M	control	0	0	0.5521	0.1134	1.517	1.1894	0.9647	0.7807	139.1708
M2control	M	control	0	0	0.5521	0.1134	1.516	1.1498	0.9661	0.7131	134.9877
M2control	M	control	0	0	0.5674	0.1191	1.834	1.1705	0.9649	0.7786	150.4054
M2Cd0	M	Cd	A	0.0000945	0.4609	0.0862	7.316	0.8701	1.1779	0.7913	165.5152

M2Cd0	M	Cd	A	0.0000945	0.5577	0.1043	8.21	1.0768	1.179	0.8205	164.3672
M2Cd0	M	Cd	A	0.0000945	0.3394	0.0635	6.422	0.9746	1.1784	0.7913	167.9058
M2Cd0	M	Cd	A	0.0000945	0.4527	0.0847	7.645	0.8717	1.2274	0.8188	165.9294
M2Cd0	M	Cd	A	0.0000945	0.4527	0.0847	6.986	1.0778	1.2282	0.7888	149.4741
M2Cd0	M	Cd	A	0.0000945	0.4666	0.0872	6.641	0.9772	1.2297	0.818	170.8798
M2Cd100	M	Cd	B	0.00945	0.0796	0.0132	224.227	0.6255	1.1594	1.2763	135.1718
M2Cd100	M	Cd	B	0.00945	0.332	0.0552	224.67	0.6321	1.1608	1.2871	136.2253
M2Cd100	M	Cd	B	0.00945	0.129	0.0214	223.784	0.6296	1.1602	1.2787	139.9783
M2Cd100	M	Cd	B	0.00945	0.1802	0.03	251.104	0.6267	1.1764	1.2884	137.1251
M2Cd100	M	Cd	B	0.00945	0.1802	0.03	197.35	0.6324	1.1783	1.2755	136.4865
M2Cd100	M	Cd	B	0.00945	0.1814	0.03	205.866	0.6318	1.1788	1.2857	149.262
M2Cd500	M	Cd	C	0.04725	0.0427	0.0065	728.021	1.1881	1.7958	1.1338	56.7841
M2Cd500	M	Cd	C	0.04725	0.0644	0.0098	728.211	1.3442	1.7973	1.1173	57.8862
M2Cd500	M	Cd	C	0.04725	0.1042	0.0158	727.831	1.2691	1.7969	1.1374	59.1142
M2Cd500	M	Cd	C	0.04725	0.0704	0.0107	788.47	1.189	1.7829	1.1178	57.9282
M2Cd500	M	Cd	C	0.04725	0.0704	0.0107	667.573	1.3448	1.7835	1.1333	52.3339
M2Cd500	M	Cd	C	0.04725	0.0729	0.0112	687.532	1.2694	1.7836	1.1173	60.7006
M2Cd1000	M	Cd	D	0.0945	0.1255	0.0202	1004.162	1.3333	2.3355	1.3	50.1999
M2Cd1000	M	Cd	D	0.0945	0.0849	0.0137	1004.702	1.358	2.3357	1.3099	52.2197
M2Cd1000	M	Cd	D	0.0945	0.1294	0.0209	1003.622	1.3457	2.3391	1.3037	55.4301
M2Cd1000	M	Cd	D	0.0945	0.1133	0.0183	1005.464	1.3355	2.3458	1.3127	52.6166
M2Cd1000	M	Cd	D	0.0945	0.1133	0.0183	1002.86	1.3587	2.3475	1.2988	51.0166
M2Cd1000	M	Cd	D	0.0945	0.1136	0.0192	1002.754	1.3475	2.3467	1.3081	53.6824
M2control	M	control	0	0	0.5598	0.115	82.584	1.1876	1.0046	0.7149	135.0545
M2control	M	control	0	0	0.347	0.0713	83.21	1.1489	1.005	0.7797	142.9999
M2control	M	control	0	0	0.7495	0.154	81.958	1.1684	1.0076	0.7139	139.4581
M2control	M	control	0	0	0.5521	0.1134	85.195	1.1894	0.9647	0.7807	139.1708
M2control	M	control	0	0	0.5521	0.1134	79.972	1.1498	0.9661	0.7131	134.9877
M2control	M	control	0	0	0.5674	0.1191	80.217	1.1705	0.9649	0.7786	150.4054
M2Zn0	M	Zn	A	0.28	0.8271	0.1277	835.053	0.6057	1.012	0.3467	139.8818
M2Zn0	M	Zn	A	0.28	0.4517	0.0697	837.779	0.6087	1.0123	0.344	140.6245

M2Zn0	M	Zn	A	0.28	0.5137	0.0793	832.327	0.6098	1.0129	0.349	141.2475
M2Zn0	M	Zn	A	0.28	0.5975	0.0923	856.122	0.6066	1.006	0.3457	140.5846
M2Zn0	M	Zn	A	0.28	0.5975	0.0923	813.984	0.6095	1.0072	0.3465	140.4337
M2Zn0	M	Zn	A	0.28	0.6173	0.093	818.281	0.611	1.0066	0.3427	153.1266
M2Zn2	M	Zn	B	0.56	0.6318	0.0872	1029.389	1.122	1.4855	0.7077	117.4377
M2Zn2	M	Zn	B	0.56	0.4116	0.0568	1030.121	1.1151	1.4867	0.7554	123.2055
M2Zn2	M	Zn	B	0.56	1.047	0.1444	1028.657	1.1203	1.4883	0.7077	117.3418
M2Zn2	M	Zn	B	0.56	0.6968	0.0961	1030.445	1.1223	1.5924	0.7551	119.3283
M2Zn2	M	Zn	B	0.56	0.6968	0.0961	1028.332	1.1171	1.5928	0.7061	118.7122
M2Zn2	M	Zn	B	0.56	0.7264	0.0963	1027.952	1.1223	1.5953	0.7546	123.8202
M2Zn4	M	Zn	C	1.12	0.256	0.0736	1511.049	2.1375	1.5858	1.3209	59.7506
M2Zn4	M	Zn	C	1.12	0.1868	0.0537	1511.998	2.1232	1.586	1.3491	59.4775
M2Zn4	M	Zn	C	1.12	0.24	0.069	1510.1	2.1305	1.588	1.3245	60.2596
M2Zn4	M	Zn	C	1.12	0.2276	0.0654	1512.773	2.1378	1.5389	1.3499	59.8292
M2Zn4	M	Zn	C	1.12	0.2276	0.0654	1509.326	2.1247	1.5404	1.3204	59.0091
M2Zn4	M	Zn	C	1.12	0.2381	0.0655	1508.951	2.1327	1.5415	1.3481	61.062
M2Zn10	M	Zn	D	2.8	0.1585	0.047	1783.038	2.4509	1.9436	1.7485	51.0465
M2Zn10	M	Zn	D	2.8	0.0361	0.0107	1784.158	2.4528	1.9444	1.7745	50.5559
M2Zn10	M	Zn	D	2.8	0.0787	0.0233	1781.918	2.4554	1.9436	1.7485	57.7216
M2Zn10	M	Zn	D	2.8	0.0911	0.027	1785.072	2.4132	1.987	1.7761	50.4669
M2Zn10	M	Zn	D	2.8	0.0911	0.027	1781.005	2.4135	1.9873	1.7474	50.3715
M2Zn10	M	Zn	D	2.8	0.0953	0.0277	1780.562	2.4164	1.9874	1.7723	51.9027
N2control	N	control	0	0	0.9841	0.1958	3.852	0.3506	1.1391	0.4466	125.3829
N2control	N	control	0	0	1.3421	0.267	4.053	0.3392	1.1404	0.4093	122.6318
N2control	N	control	0	0	0.7551	0.1502	3.651	0.345	1.142	0.4505	128.603
N2control	N	control	0	0	1.027	0.2043	4.08	0.3512	1.116	0.4114	125.5392
N2control	N	control	0	0	1.0271	0.2043	3.625	0.3394	1.1174	0.4455	120.5056
N2control	N	control	0	0	1.0353	0.2132	3.5	0.3457	1.1179	0.408	130.5224
N2Cu1/2	N	Cu	A	0.13	0.0523	0.0107	18.499	0.5946	1.1375	0.3487	115.9878
N2Cu1/2	N	Cu	A	0.13	0.0557	0.0114	18.952	0.5993	1.1385	0.3519	115.5858
N2Cu1/2	N	Cu	A	0.13	0.0303	0.0062	18.046	0.5983	1.1386	0.3493	118.0146

N2Cu1/2	N	Cu	A	0.13	0.0458	0.0094	20.392	0.5951	1.1583	0.3522	116.5294
N2Cu1/2	N	Cu	A	0.13	0.046	0.0094	16.607	0.5995	1.1594	0.3476	114.1836
N2Cu1/2	N	Cu	A	0.13	0.0471	0.0097	16.785	0.5985	1.1606	0.3502	125.3421
N2Cu0	N	Cu	B	0.26	0.0422	0.0095	46.459	0.6082	1.1133	0.3469	116.2885
N2Cu0	N	Cu	B	0.26	0.0682	0.0154	46.827	0.646	1.1134	0.3847	113.1618
N2Cu0	N	Cu	B	0.26	0.0142	0.0032	46.091	0.6164	1.1165	0.3502	119.9567
N2Cu0	N	Cu	B	0.26	0.0413	0.0093	48.804	0.609	1.1076	0.3836	116.469
N2Cu0	N	Cu	B	0.26	0.0415	0.0094	44.114	0.646	1.1078	0.345	108.0921
N2Cu0	N	Cu	B	0.26	0.0429	0.0095	44.527	0.6172	1.1096	0.3831	126.6351
N2Cu2.5	N	Cu	C	0.65	0.0431	0.0113	211.396	0.4778	1.2675	0.3106	113.8276
N2Cu2.5	N	Cu	C	0.65	0.0401	0.0105	211.515	0.4651	1.2691	0.3647	112.8803
N2Cu2.5	N	Cu	C	0.65	0.0361	0.0095	211.277	0.4716	1.2686	0.3106	117.6149
N2Cu2.5	N	Cu	C	0.65	0.0397	0.0104	212.623	0.4784	1.239	0.3661	114.7743
N2Cu2.5	N	Cu	C	0.65	0.0398	0.0104	210.17	0.4652	1.2397	0.3106	112.4538
N2Cu2.5	N	Cu	C	0.65	0.0416	0.0107	210.46	0.4719	1.2426	0.3635	119.7547
N2Cu5	N	Cu	D	1.3	0.0042	0.0013	449.026	0.978	1.3892	0.5966	56.0942
N2Cu5	N	Cu	D	1.3	0.0022	0.0007	410.5268	0.9926	1.3893	0.6017	51.2375
N2Cu5	N	Cu	D	1.3	0.0142	0.0043	368.9785	0.9855	1.3918	0.5972	51.019
N2Cu5	N	Cu	D	1.3	0.0067	0.002	391.8683	0.9785	1.4018	0.6008	54.4701
N2Cu5	N	Cu	D	1.3	0.0068	0.0021	383.7998	0.9927	1.4037	0.594	58.9301
N2Cu5	N	Cu	D	1.3	0.0071	0.0022	363.776	0.9856	1.402	0.6005	52.945
N2control	N	control	0	0	0.9841	0.1958	0.289	0.3506	1.1391	0.4466	125.3829
N2control	N	control	0	0	1.3421	0.267	0.331	0.3392	1.1404	0.4093	122.6318
N2control	N	control	0	0	0.7551	0.1502	0.347	0.345	1.142	0.4505	128.603
N2control	N	control	0	0	1.027	0.2043	0.349	0.3512	1.116	0.4114	125.5392
N2control	N	control	0	0	1.0271	0.2043	0.388	0.3394	1.1174	0.4455	120.5056
N2control	N	control	0	0	1.0353	0.2132	0.313	0.3457	1.1179	0.408	130.5224
N2Pb0	N	Pb	A	0.025	0.5572	0.1305	9.05	0.2022	1.2036	0.4173	114.6646
N2Pb0	N	Pb	A	0.025	0.0744	0.0174	11.453	0.14	1.2042	0.4192	120.58
N2Pb0	N	Pb	A	0.025	0.6672	0.1563	10.647	0.1207	1.2054	0.4219	116.3345
N2Pb0	N	Pb	A	0.025	0.4327	0.1014	9.447	0.2025	1.1763	0.4192	117.193

N2Pb0	N	Pb	A	0.025	0.4329	0.1014	8.653	0.1402	1.1771	0.4167	114.0341
N2Pb0	N	Pb	A	0.025	0.4492	0.1014	10.383	0.1209	1.1794	0.4184	128.4413
N2Pb10	N	Pb	B	0.25	0.0533	0.0133	190.799	0.3258	1.1866	0.3756	141.408
N2Pb10	N	Pb	B	0.25	0.0373	0.0093	190.805	0.3091	1.1876	0.4341	148.0912
N2Pb10	N	Pb	B	0.25	0.0493	0.0123	190.793	0.3175	1.1891	0.3767	138.9137
N2Pb10	N	Pb	B	0.25	0.0463	0.0115	195.236	0.3261	1.1633	0.435	142.8043
N2Pb10	N	Pb	B	0.25	0.0466	0.0116	186.361	0.3092	1.1633	0.3727	138.6348
N2Pb10	N	Pb	B	0.25	0.0482	0.0116	187.835	0.3176	1.1657	0.4313	149.5931
N2Pb50	N	Pb	C	1.25	0.0543	0.0132	2950.804	0.6999	1.2514	0.3291	62.3871
N2Pb50	N	Pb	C	1.25	0.0213	0.0052	2950.841	0.7186	1.2523	0.2928	61.4025
N2Pb50	N	Pb	C	1.25	0.0243	0.0059	2950.767	0.7254	1.2535	0.3317	68.5082
N2Pb50	N	Pb	C	1.25	0.033	0.008	2953.974	0.7004	1.2711	0.2915	64.0993
N2Pb50	N	Pb	C	1.25	0.0332	0.0081	2947.635	0.7193	1.2732	0.3285	61.0551
N2Pb50	N	Pb	C	1.25	0.0339	0.0084	2948.654	0.726	1.275	0.2902	67.9181
N2Pb100	N	Pb	D	2.5	0.0123	0.003	5360.563	0.9275	1.2803	0.4237	65.6326
N2Pb100	N	Pb	D	2.5	0.0133	0.0032	5361.232	0.8826	1.2818	0.447	65.8126
N2Pb100	N	Pb	D	2.5	0.0193	0.0046	5359.894	0.9784	1.2813	0.4278	71.4787
N2Pb100	N	Pb	D	2.5	0.0147	0.0035	5437.4	0.9278	1.3095	0.446	67.6413
N2Pb100	N	Pb	D	2.5	0.0149	0.0036	5283.726	0.8831	1.3097	0.4218	64.944
N2Pb100	N	Pb	D	2.5	0.0156	0.0036	5308.669	0.9787	1.311	0.4451	70.2151
N2control	N	control	0	0	0.9841	0.1958	1.638	0.3506	1.1391	0.4466	125.3829
N2control	N	control	0	0	1.3421	0.267	1.689	0.3392	1.1404	0.4093	122.6318
N2control	N	control	0	0	0.7551	0.1502	1.587	0.345	1.142	0.4505	128.603
N2control	N	control	0	0	1.027	0.2043	1.879	0.3512	1.116	0.4114	125.5392
N2control	N	control	0	0	1.0271	0.2043	1.397	0.3394	1.1174	0.4455	120.5056
N2control	N	control	0	0	1.0353	0.2132	1.427	0.3457	1.1179	0.408	130.5224
N2Cr0	N	Cr	A	0.036	0.2641	0.06	13.39	0.2778	1.0964	0.2786	153.0653
N2Cr0	N	Cr	A	0.036	0.6421	0.1458	13.613	0.2923	1.0974	0.2354	151.2827
N2Cr0	N	Cr	A	0.036	0.4181	0.0949	13.167	0.2974	1.0996	0.2806	154.2307
N2Cr0	N	Cr	A	0.036	0.4413	0.1002	15.261	0.2782	1.1097	0.2374	152.8596
N2Cr0	N	Cr	A	0.036	0.4414	0.1002	11.518	0.2928	1.1108	0.2778	150.7839

N2Cr0	N	Cr	A	0.036	0.4512	0.1044	11.919	0.298	1.1128	0.2346	156.4407
N2Cr5	N	Cr	B	0.18	0.3213	0.0844	4.001	0.4455	1.2803	0.3974	138.3252
N2Cr5	N	Cr	B	0.18	0.0843	0.0221	4.038	0.4481	1.2818	0.3994	141.3926
N2Cr5	N	Cr	B	0.18	0.0115	0.003	3.964	0.4477	1.2831	0.3974	139.7425
N2Cr5	N	Cr	B	0.18	0.1387	0.0364	4.119	0.446	1.2544	0.4023	139.8201
N2Cr5	N	Cr	B	0.18	0.139	0.0365	3.883	0.4482	1.2549	0.3955	129.9681
N2Cr5	N	Cr	B	0.18	0.1397	0.0371	3.885	0.4485	1.2569	0.3989	142.0526
N2Cr10	N	Cr	C	0.36	0.0511	0.0134	58.803	0.5723	1.2592	0.3848	133.8209
N2Cr10	N	Cr	C	0.36	0.0841	0.022	58.85	0.5815	1.2595	0.4028	134.1327
N2Cr10	N	Cr	C	0.36	0.0731	0.0191	58.756	0.5778	1.2592	0.3873	136.9526
N2Cr10	N	Cr	C	0.36	0.0693	0.0181	60.286	0.573	1.2603	0.4039	134.9687
N2Cr10	N	Cr	C	0.36	0.0694	0.0182	57.319	0.5816	1.2618	0.382	126.9481
N2Cr10	N	Cr	C	0.36	0.0712	0.0187	57.766	0.5783	1.2626	0.4002	138.1036
N2Cr25	N	Cr	D	0.9	0.041	0.009	480.442	1.1301	1.4961	0.4559	98.8813
N2Cr25	N	Cr	D	0.9	0.051	0.0112	482.242	1.188	1.4975	0.4942	98.508
N2Cr25	N	Cr	D	0.9	0.024	0.0053	478.642	1.188	1.4972	0.4583	99.2001
N2Cr25	N	Cr	D	0.9	0.0387	0.0085	491.395	1.1301	1.4972	0.4969	98.8632
N2Cr25	N	Cr	D	0.9	0.0387	0.0085	469.488	1.1885	1.4986	0.4559	97.4635
N2Cr25	N	Cr	D	0.9	0.039	0.0089	471.339	1.1887	1.4974	0.4912	102.6872
N2control	N	control	0	0	0.9841	0.1958	2.442	0.3506	1.1391	0.4466	125.3829
N2control	N	control	0	0	1.3421	0.267	2.502	0.3392	1.1404	0.4093	122.6318
N2control	N	control	0	0	0.7551	0.1502	2.382	0.345	1.142	0.4505	128.603
N2control	N	control	0	0	1.027	0.2043	2.543	0.3512	1.116	0.4114	125.5392
N2control	N	control	0	0	1.0271	0.2043	2.34	0.3394	1.1174	0.4455	120.5056
N2control	N	control	0	0	1.0353	0.2132	2.314	0.3457	1.1179	0.408	130.5224
N2Ni0	N	Ni	A	0.025	0.7472	0.1601	48.959	0.402	1.1231	0.3071	136.0864
N2Ni0	N	Ni	A	0.025	0.6372	0.1365	49.728	0.4035	1.1246	0.3543	137.6464
N2Ni0	N	Ni	A	0.025	0.4362	0.0935	48.19	0.4035	1.1239	0.3055	139.7925
N2Ni0	N	Ni	A	0.025	0.6067	0.13	49.067	0.4025	1.1114	0.3549	137.8418
N2Ni0	N	Ni	A	0.025	0.6068	0.13	48.85	0.4037	1.1132	0.3044	134.9325
N2Ni0	N	Ni	A	0.025	0.6175	0.1325	48.118	0.4042	1.1132	0.3521	149.6299

N2Ni5	N	Ni	B	0.125	0.3373	0.0887	281.603	0.3184	1.1345	0.3126	75.557
N2Ni5	N	Ni	B	0.125	0.8983	0.2363	281.642	0.2448	1.1353	0.3666	76.4866
N2Ni5	N	Ni	B	0.125	0.2953	0.0777	281.564	0.2819	1.1377	0.3134	76.7096
N2Ni5	N	Ni	B	0.125	0.51	0.1342	303.319	0.3188	1.0915	0.3647	76.251
N2Ni5	N	Ni	B	0.125	0.5102	0.1342	259.887	0.2456	1.0925	0.3109	73.0824
N2Ni5	N	Ni	B	0.125	0.5106	0.1403	267.086	0.2821	1.0918	0.3644	82.1252
N2Ni10	N	Ni	C	0.25	0.463	0.1135	430.402	0.3802	1.0888	0.3522	56.9666
N2Ni10	N	Ni	C	0.25	0.465	0.114	430.823	0.3633	1.0903	0.3665	58.6545
N2Ni10	N	Ni	C	0.25	0.369	0.0905	429.981	0.3819	1.0902	0.3509	61.6088
N2Ni10	N	Ni	C	0.25	0.4323	0.106	434.872	0.3808	1.0651	0.3678	59.0767
N2Ni10	N	Ni	C	0.25	0.4323	0.106	425.932	0.3637	1.0668	0.3509	57.669
N2Ni10	N	Ni	C	0.25	0.4395	0.1061	427.001	0.382	1.067	0.3643	59.9437
N2Ni25	N	Ni	D	0.625	0.094	0.0223	447.6181	1.0728	1.592	0.6324	43.3895
N2Ni25	N	Ni	D	0.625	0.132	0.0314	448.0559	1.0588	1.5926	0.6311	50.2104
N2Ni25	N	Ni	D	0.625	0.153	0.0363	447.1802	1.1307	1.5948	0.6334	49.0634
N2Ni25	N	Ni	D	0.625	0.1263	0.03	452.2669	1.0736	1.5901	0.6318	47.0137
N2Ni25	N	Ni	D	0.625	0.1263	0.03	442.9693	1.0591	1.5916	0.6295	42.8158
N2Ni25	N	Ni	D	0.625	0.1276	0.0308	444.081	1.1312	1.5927	0.6295	54.4996
N2control	N	control	0	0	0.9841	0.1958	0.406	0.3506	1.1391	0.4466	125.3829
N2control	N	control	0	0	1.3421	0.267	0.441	0.3392	1.1404	0.4093	122.6318
N2control	N	control	0	0	0.7551	0.1502	0.445	0.345	1.142	0.4505	128.603
N2control	N	control	0	0	1.027	0.2043	0.441	0.3512	1.116	0.4114	125.5392
N2control	N	control	0	0	1.0271	0.2043	0.444	0.3394	1.1174	0.4455	120.5056
N2control	N	control	0	0	1.0353	0.2132	0.451	0.3457	1.1179	0.408	130.5224
N2Cd0	N	Cd	A	0.0000945	0.4661	0.096	15.682	0.2648	1.0875	0.5305	131.133
N2Cd0	N	Cd	A	0.0000945	0.5141	0.1059	16.609	0.3275	1.0889	0.5123	128.6669
N2Cd0	N	Cd	A	0.0000945	0.3491	0.0719	14.755	0.2965	1.0881	0.5324	135.4997
N2Cd0	N	Cd	A	0.0000945	0.443	0.0912	16.449	0.2649	1.119	0.5121	131.7665
N2Cd0	N	Cd	A	0.0000945	0.4431	0.0913	14.915	0.3277	1.1197	0.5299	120.2784
N2Cd0	N	Cd	A	0.0000945	0.4432	0.0922	15.266	0.2967	1.1207	0.5104	134.6499
N2Cd100	N	Cd	B	0.00945	0.081	0.0176	300.798	0.3158	0.9288	0.321	135.4652

N2Cd100	N	Cd	B	0.00945	0.325	0.0707	301.78	0.3078	0.9304	0.3047	135.8541
N2Cd100	N	Cd	B	0.00945	0.152	0.0331	299.816	0.3128	0.9305	0.32	138.3243
N2Cd100	N	Cd	B	0.00945	0.186	0.0405	310.384	0.3163	0.9387	0.3057	136.5479
N2Cd100	N	Cd	B	0.00945	0.186	0.0405	291.212	0.3078	0.94	0.3188	129.2666
N2Cd100	N	Cd	B	0.00945	0.1915	0.0422	306.207	0.3134	0.9414	0.3042	141.9326
N2Cd500	N	Cd	C	0.04725	0.0543	0.011	663.634	0.3323	0.9668	0.1744	68.1641
N2Cd500	N	Cd	C	0.04725	0.0633	0.0128	664.986	0.3759	0.9675	0.2323	67.4083
N2Cd500	N	Cd	C	0.04725	0.1133	0.0229	662.282	0.355	0.9696	0.1739	73.5819
N2Cd500	N	Cd	C	0.04725	0.0767	0.0155	731.31	0.3326	0.978	0.2345	69.7181
N2Cd500	N	Cd	C	0.04725	0.0769	0.0156	595.959	0.3763	0.9794	0.1736	68.8618
N2Cd500	N	Cd	C	0.04725	0.0781	0.0156	707.399	0.355	0.9797	0.2318	74.2632
N2Cd1000	N	Cd	D	0.0945	0.0029	0.0005	646.18	0.5574	0.9737	0.1109	39.0114
N2Cd1000	N	Cd	D	0.0945	0.0081	0.0015	646.543	0.567	0.9753	0.1854	39.4403
N2Cd1000	N	Cd	D	0.0945	0.0321	0.0059	645.817	0.5946	0.974	0.1106	42.6513
N2Cd1000	N	Cd	D	0.0945	0.0143	0.0026	696.688	0.558	0.9632	0.1871	40.3676
N2Cd1000	N	Cd	D	0.0945	0.0144	0.0026	595.672	0.5672	0.9635	0.1094	38.5593
N2Cd1000	N	Cd	D	0.0945	0.015	0.0027	679.489	0.5952	0.9658	0.1839	43.7673
N2control	N	control	0	0	0.9841	0.1958	23.306	0.3506	1.1391	0.4466	125.3829
N2control	N	control	0	0	1.3421	0.267	24.269	0.3392	1.1404	0.4093	122.6318
N2control	N	control	0	0	0.7551	0.1502	22.343	0.345	1.142	0.4505	128.603
N2control	N	control	0	0	1.027	0.2043	25.374	0.3512	1.116	0.4114	125.5392
N2control	N	control	0	0	1.0271	0.2043	21.237	0.3394	1.1174	0.4455	120.5056
N2control	N	control	0	0	1.0353	0.2132	23.722	0.3457	1.1179	0.408	130.5224
N2Zn0	N	Zn	A	0.28	0.8271	0.1866	1304.169	0.3513	1.1065	0.3479	113.3539
N2Zn0	N	Zn	A	0.28	0.5721	0.1291	1304.756	0.3492	1.1084	0.3197	113.6191
N2Zn0	N	Zn	A	0.28	0.5191	0.1171	1303.582	0.3508	1.1072	0.3484	113.6921
N2Zn0	N	Zn	A	0.28	0.6393	0.1442	1306.456	0.3517	1.0974	0.3206	113.555
N2Zn0	N	Zn	A	0.28	0.6394	0.1443	1301.882	0.3492	1.0989	0.347	104.1588
N2Zn0	N	Zn	A	0.28	0.6665	0.151	1305.107	0.351	1.0986	0.3177	123.9913
N2Zn2	N	Zn	B	0.56	0.6492	0.1485	1266.127	0.3488	1.1136	0.35	74.4298
N2Zn2	N	Zn	B	0.56	0.4092	0.0936	1267.141	0.3552	1.115	0.3862	74.0218

N2Zn2	N	Zn	B	0.56	0.4612	0.1055	1265.113	0.352	1.1163	0.3521	78.2942
N2Zn2	N	Zn	B	0.56	0.5063	0.1158	1268.768	0.3489	1.0911	0.3886	75.5819
N2Zn2	N	Zn	B	0.56	0.5065	0.1159	1263.487	0.3554	1.0923	0.3481	75.353
N2Zn2	N	Zn	B	0.56	0.5085	0.1202	1266.874	0.3526	1.094	0.3854	83.43
N2Zn4	N	Zn	C	1.12	0.2612	0.0644	1677.482	0.5811	1.7674	0.7309	48.7013
N2Zn4	N	Zn	C	1.12	0.1852	0.0457	1679.105	0.5736	1.7675	0.7312	50.2104
N2Zn4	N	Zn	C	1.12	0.2402	0.0593	1675.859	0.5889	1.7695	0.7322	52.9416
N2Zn4	N	Zn	C	1.12	0.2287	0.0564	1680.195	0.5815	1.7259	0.7341	50.6178
N2Zn4	N	Zn	C	1.12	0.2288	0.0565	1674.769	0.5741	1.7269	0.7296	48.6687
N2Zn4	N	Zn	C	1.12	0.2296	0.0586	1677.667	0.5895	1.7286	0.7298	54.4996
N2Zn10	N	Zn	D	2.8	0.0491	0.0128	1929.104	1.3704	2.2942	1.1133	47.0643
N2Zn10	N	Zn	D	2.8	0.0291	0.0076	1930.971	1.3035	2.2952	1.09	42.6789
N2Zn10	N	Zn	D	2.8	0.0891	0.0232	1927.238	1.252	2.2966	1.1133	51.1895
N2Zn10	N	Zn	D	2.8	0.0557	0.0145	1932.224	1.3711	2.2954	1.0924	50.8548
N2Zn10	N	Zn	D	2.8	0.0558	0.0145	1925.984	1.3043	2.2974	1.1122	46.6764
N2Zn10	N	Zn	D	2.8	0.0575	0.0148	1929.317	1.2527	2.2972	1.0882	46.3247
T2control	T	control	0	0	0.5988	0.1125	7.515	0.3348	1.0692	0.5697	142.3843
T2control	T	control	0	0	1.2097	0.2272	7.824	0.3239	1.0707	0.5724	141.3293
T2control	T	control	0	0	1.0061	0.189	7.206	0.3294	1.0694	0.5692	149.1246
T2control	T	control	0	0	0.9382	0.1762	7.97	0.3349	1.0627	0.5719	144.2794
T2control	T	control	0	0	0.9382	0.1762	7.059	0.3245	1.063	0.5677	140.1134
T2control	T	control	0	0	0.9694	0.1816	6.902	0.3295	1.0656	0.5707	158.9114
T2Cu1/2	T	Cu	A	0.13	0.0802	0.0173	37.351	0.351	1.2116	0.6212	137.7592
T2Cu1/2	T	Cu	A	0.13	0.2321	0.05	37.929	0.3055	1.2124	0.6507	135.8613
T2Cu1/2	T	Cu	A	0.13	0.0972	0.0209	36.773	0.305	1.2126	0.624	142.7944
T2Cu1/2	T	Cu	A	0.13	0.1365	0.0294	38.259	0.3516	1.2275	0.6513	138.805
T2Cu1/2	T	Cu	A	0.13	0.1365	0.0294	36.442	0.3062	1.2287	0.6203	133.4293
T2Cu1/2	T	Cu	A	0.13	0.1418	0.0296	36.167	0.3056	1.2287	0.6482	139.2649
T2Cu0	T	Cu	B	0.26	0.0995	0.0276	66.128	0.5966	1.1413	0.744	119.4475
T2Cu0	T	Cu	B	0.26	0.0663	0.0184	66.378	0.5786	1.1426	0.7546	117.7579
T2Cu0	T	Cu	B	0.26	0.0638	0.0177	65.878	0.5879	1.1448	0.7451	124.9202

T2Cu0	T	Cu	B	0.26	0.0765	0.0212	68.354	0.5974	1.1344	0.756	120.7085
T2Cu0	T	Cu	B	0.26	0.0765	0.0212	68.205	0.5789	1.1347	0.7434	118.0718
T2Cu0	T	Cu	B	0.26	0.0766	0.0215	65.878	0.5884	1.1358	0.7537	121.833
T2Cu2.5	T	Cu	C	0.65	0.0042	0.001	538.5824	0.675	1.8245	0.8531	89.6574
T2Cu2.5	T	Cu	C	0.65	0.0066	0.0016	539.3824	0.657	1.826	0.909	88.4214
T2Cu2.5	T	Cu	C	0.65	0.0056	0.0014	537.7824	0.6661	1.8252	0.8539	92.3778
T2Cu2.5	T	Cu	C	0.65	0.0055	0.0014	596.9648	0.6759	1.8252	0.9082	90.1522
T2Cu2.5	T	Cu	C	0.65	0.0055	0.0014	480.2008	0.6577	1.8274	0.8522	86.2967
T2Cu2.5	T	Cu	C	0.65	0.0055	0.0014	498.8616	0.6669	1.8302	0.9069	94.3782
T2Cu5	T	Cu	D	1.3	0.0185	0.0056	575.24	1.142	1.9892	1.0096	59.1281
T2Cu5	T	Cu	D	1.3	0.0141	0.0043	643.1474	1.0728	1.9905	0.9943	54.6506
T2Cu5	T	Cu	D	1.3	0.0003	0.0001	579.6105	1.0971	1.9934	1.0092	51.9755
T2Cu5	T	Cu	D	1.3	0.011	0.0033	615.6045	1.1423	1.8439	0.9975	55.4992
T2Cu5	T	Cu	D	1.3	0.011	0.0033	589.0541	1.0738	1.8454	1.0071	60.0605
T2Cu5	T	Cu	D	1.3	0.0114	0.0034	573.3809	1.098	1.847	0.9932	53.5005
T2control	T	control	0	0	0.5988	0.1125	1.434	0.3348	1.0692	0.5697	142.3843
T2control	T	control	0	0	1.2097	0.2272	1.459	0.3239	1.0707	0.5724	141.3293
T2control	T	control	0	0	1.0061	0.189	1.409	0.3294	1.0694	0.5692	149.1246
T2control	T	control	0	0	0.9382	0.1762	1.536	0.3349	1.0627	0.5719	144.2794
T2control	T	control	0	0	0.9382	0.1762	1.331	0.3245	1.063	0.5677	140.1134
T2control	T	control	0	0	0.9694	0.1816	1.34	0.3295	1.0656	0.5707	158.9114
T2Pb0	T	Pb	A	0.025	0.7197	0.1716	16.831	0.2653	0.9683	0.5588	146.3062
T2Pb0	T	Pb	A	0.025	0.8006	0.1909	17.193	0.2667	0.9691	0.58	143.2838
T2Pb0	T	Pb	A	0.025	0.1567	0.0374	16.469	0.2663	0.9696	0.5623	150.2362
T2Pb0	T	Pb	A	0.025	0.559	0.1333	17.44	0.2655	0.9608	0.581	146.6087
T2Pb0	T	Pb	A	0.025	0.559	0.1333	16.223	0.2672	0.9624	0.5578	136.7309
T2Pb0	T	Pb	A	0.025	0.5825	0.1373	16.063	0.2668	0.9609	0.579	159.4662
T2Pb10	T	Pb	B	0.25	0.06	0.0105	194.342	0.3154	1.0596	0.5802	131.5495
T2Pb10	T	Pb	B	0.25	0.0587	0.0103	194.344	0.2992	1.0596	0.594	129.6693
T2Pb10	T	Pb	B	0.25	0.0697	0.0122	194.34	0.3074	1.0625	0.5797	137.1198
T2Pb10	T	Pb	B	0.25	0.0628	0.011	196.035	0.3157	1.0357	0.5962	132.7795

T2Pb10	T	Pb	B	0.25	0.0628	0.011	192.648	0.2996	1.0362	0.5778	120.0214
T2Pb10	T	Pb	B	0.25	0.0659	0.0111	193.211	0.3074	1.0358	0.5929	140.0651
T2Pb50	T	Pb	C	1.25	0.0216	0.0058	1132.747	1.0298	1.7474	1.2172	79.771
T2Pb50	T	Pb	C	1.25	0.0258	0.007	1132.866	1.0195	1.7486	1.2534	81.2797
T2Pb50	T	Pb	C	1.25	0.0201	0.0054	1132.628	1.0111	1.7477	1.2158	88.9017
T2Pb50	T	Pb	C	1.25	0.0225	0.0061	1179.79	1.031	1.7257	1.2544	83.3175
T2Pb50	T	Pb	C	1.25	0.0225	0.0061	1085.705	1.02	1.7257	1.2128	82.565
T2Pb50	T	Pb	C	1.25	0.0232	0.0061	1101.267	1.0111	1.7286	1.25	84.8197
T2Pb100	T	Pb	D	2.5	0.017	0.0057	3851.341	1.3169	2.3687	1.2528	59.7506
T2Pb100	T	Pb	D	2.5	0.0056	0.0019	3851.746	1.2693	2.3706	1.2717	59.4775
T2Pb100	T	Pb	D	2.5	0.0084	0.0028	3850.937	1.3259	2.3715	1.249	60.2596
T2Pb100	T	Pb	D	2.5	0.0103	0.0035	4011.285	1.3179	2.377	1.2751	59.8292
T2Pb100	T	Pb	D	2.5	0.0103	0.0035	3691.397	1.2697	2.3778	1.248	59.0091
T2Pb100	T	Pb	D	2.5	0.0105	0.0036	3744.307	1.3262	2.377	1.2703	61.062
T2control	T	control	0	0	0.5988	0.1125	2.756	0.3348	1.0692	0.5697	142.3843
T2control	T	control	0	0	1.2097	0.2272	2.86	0.3239	1.0707	0.5724	141.3293
T2control	T	control	0	0	1.0061	0.189	2.652	0.3294	1.0694	0.5692	149.1246
T2control	T	control	0	0	0.9382	0.1762	3.053	0.3349	1.0627	0.5719	144.2794
T2control	T	control	0	0	0.9382	0.1762	2.458	0.3245	1.063	0.5677	140.1134
T2control	T	control	0	0	0.9694	0.1816	2.453	0.3295	1.0656	0.5707	158.9114
T2Cr0	T	Cr	A	0.036	0.5732	0.1185	10.325	0.2834	1.103	0.5447	142.4156
T2Cr0	T	Cr	A	0.036	0.1371	0.0283	10.708	0.2839	1.1046	0.5525	145.7447
T2Cr0	T	Cr	A	0.036	0.3215	0.0664	9.942	0.2845	1.1046	0.5447	142.6822
T2Cr0	T	Cr	A	0.036	0.3439	0.0711	10.36	0.2838	1.0901	0.5558	143.6142
T2Cr0	T	Cr	A	0.036	0.3439	0.0711	10.289	0.2846	1.0909	0.5433	139.8857
T2Cr0	T	Cr	A	0.036	0.3467	0.0736	9.918	0.2847	1.0906	0.552	147.3842
T2Cr5	T	Cr	B	0.18	0.2688	0.0606	16.992	0.4074	1.17	0.5115	135.1012
T2Cr5	T	Cr	B	0.18	0.1078	0.0243	17.109	0.4097	1.1705	0.4873	135.7296
T2Cr5	T	Cr	B	0.18	0.2503	0.0565	16.875	0.4094	1.1717	0.5162	139.7178
T2Cr5	T	Cr	B	0.18	0.209	0.0471	17.878	0.4077	1.1941	0.4863	136.8495
T2Cr5	T	Cr	B	0.18	0.209	0.0471	16.106	0.4104	1.195	0.5112	124.2843

T2Cr5	T	Cr	B	0.18	0.2125	0.0485	17.465	0.4097	1.1944	0.4858	143.86
T2Cr10	T	Cr	C	0.36	0.0719	0.0184	70.047	0.4038	1.2764	0.6202	124.7549
T2Cr10	T	Cr	C	0.36	0.0818	0.0209	70.074	0.3857	1.2781	0.6002	125.5912
T2Cr10	T	Cr	C	0.36	0.0803	0.0205	70.02	0.3745	1.2787	0.6223	126.191
T2Cr10	T	Cr	C	0.36	0.078	0.0199	70.331	0.4042	1.3301	0.5989	125.5124
T2Cr10	T	Cr	C	0.36	0.078	0.0199	69.763	0.3858	1.3318	0.6184	113.6359
T2Cr10	T	Cr	C	0.36	0.0785	0.0209	70.21	0.3746	1.3329	0.5989	132.7179
T2Cr25	T	Cr	D	0.9	0.1471	0.0417	958.451	0.934	2.4196	0.7025	132.756
T2Cr25	T	Cr	D	0.9	0.0424	0.012	959.658	0.8013	2.4196	0.7333	169.3311
T2Cr25	T	Cr	D	0.9	0.0513	0.0146	957.244	0.9148	2.4225	0.702	141.9831
T2Cr25	T	Cr	D	0.9	0.0803	0.0228	959.063	0.9348	2.532	0.7315	148.0234
T2Cr25	T	Cr	D	0.9	0.0803	0.0228	957.838	0.8024	2.5328	0.7011	140.092
T2Cr25	T	Cr	D	0.9	0.0831	0.0234	957.652	0.9152	2.5354	0.7306	156.6803
T2control	T	control	0	0	0.5988	0.1125	5.077	0.3348	1.0692	0.5697	142.3843
T2control	T	control	0	0	1.2097	0.2272	5.4	0.3239	1.0707	0.5724	141.3293
T2control	T	control	0	0	1.0061	0.189	4.754	0.3294	1.0694	0.5692	149.1246
T2control	T	control	0	0	0.9382	0.1762	5.143	0.3349	1.0627	0.5719	144.2794
T2control	T	control	0	0	0.9382	0.1762	5.011	0.3245	1.063	0.5677	140.1134
T2control	T	control	0	0	0.9694	0.1816	4.798	0.3295	1.0656	0.5707	158.9114
T2Ni0	T	Ni	A	0.025	0.9127	0.1845	32.239	0.26	1.022	0.5192	169.7534
T2Ni0	T	Ni	A	0.025	0.6272	0.1268	32.351	0.251	1.0237	0.5602	169.0549
T2Ni0	T	Ni	A	0.025	0.9523	0.1925	32.127	0.2565	1.024	0.5187	171.6658
T2Ni0	T	Ni	A	0.025	0.8307	0.1679	33.445	0.2605	1.0107	0.5588	170.158
T2Ni0	T	Ni	A	0.025	0.8307	0.1679	31.034	0.2514	1.0119	0.5173	158.1105
T2Ni0	T	Ni	A	0.025	0.8706	0.175	32.931	0.2568	1.0135	0.5577	177.2054
T2Ni5	T	Ni	B	0.125	0.4177	0.0721	137.617	0.3127	1.116	0.5331	97.5095
T2Ni5	T	Ni	B	0.125	0.3774	0.0651	137.953	0.2406	1.1171	0.5312	98.7436
T2Ni5	T	Ni	B	0.125	0.3976	0.0686	137.281	0.2769	1.1161	0.5386	101.1381
T2Ni5	T	Ni	B	0.125	0.3976	0.0686	153.764	0.3129	1.1174	0.5353	99.1304
T2Ni5	T	Ni	B	0.125	0.3976	0.0686	121.471	0.2413	1.1187	0.5331	95.314
T2Ni5	T	Ni	B	0.125	0.4069	0.0704	148.046	0.277	1.1195	0.5301	106.3533

T2Ni10	T	Ni	C	0.25	0.4502	0.1182	370.1457	0.3584	1.0617	0.4974	76.0959
T2Ni10	T	Ni	C	0.25	0.3721	0.0977	370.5078	0.3493	1.0622	0.5131	77.1385
T2Ni10	T	Ni	C	0.25	0.2723	0.0715	369.7837	0.3549	1.0632	0.4993	80.8459
T2Ni10	T	Ni	C	0.25	0.3649	0.0958	373.9899	0.3591	1.0627	0.5142	78.0267
T2Ni10	T	Ni	C	0.25	0.3649	0.0958	366.3015	0.3496	1.063	0.4954	72.8354
T2Ni10	T	Ni	C	0.25	0.3828	0.1002	367.2209	0.3551	1.065	0.5128	80.5046
T2Ni25	T	Ni	D	0.625	0.0732	0.0196	392.3545	0.5927	1.4537	0.7044	47.1835
T2Ni25	T	Ni	D	0.625	0.1366	0.0367	392.7382	0.6143	1.4556	0.7179	49.498
T2Ni25	T	Ni	D	0.625	0.1274	0.0342	391.9707	0.5979	1.4565	0.7047	53.1974
T2Ni25	T	Ni	D	0.625	0.1124	0.0302	396.4293	0.5931	1.4916	0.7197	50.6178
T2Ni25	T	Ni	D	0.625	0.1124	0.0302	388.2796	0.6147	1.4934	0.7038	48.8958
T2Ni25	T	Ni	D	0.625	0.1168	0.031	389.2541	0.5981	1.493	0.717	56.213
T2control	T	control	0	0	0.5988	0.1125	0.547	0.3348	1.0692	0.5697	142.3843
T2control	T	control	0	0	1.2097	0.2272	0.504	0.3239	1.0707	0.5724	141.3293
T2control	T	control	0	0	1.0061	0.189	0.529	0.3294	1.0694	0.5692	149.1246
T2control	T	control	0	0	0.9382	0.1762	0.56	0.3349	1.0627	0.5719	144.2794
T2control	T	control	0	0	0.9382	0.1762	0.55	0.3245	1.063	0.5677	140.1134
T2control	T	control	0	0	0.9694	0.1816	0.592	0.3295	1.0656	0.5707	158.9114
T2Cd0	T	Cd	A	0.0000945	0.7028	0.1464	11.408	0.184	1.1471	0.737	142.6038
T2Cd0	T	Cd	A	0.0000945	0.4004	0.0834	13.142	0.186	1.1477	0.6763	144.1258
T2Cd0	T	Cd	A	0.0000945	0.8514	0.1773	9.674	0.1852	1.1473	0.7388	144.0714
T2Cd0	T	Cd	A	0.0000945	0.6515	0.1357	11.848	0.1846	1.1466	0.6786	143.6003
T2Cd0	T	Cd	A	0.0000945	0.6515	0.1357	10.967	0.1866	1.1474	0.7357	142.5135
T2Cd0	T	Cd	A	0.0000945	0.6768	0.1372	9.967	0.186	1.1479	0.6763	149.7512
T2Cd100	T	Cd	B	0.00945	0.6953	0.1376	39.565	0.2964	1.2079	0.5119	31.2459
T2Cd100	T	Cd	B	0.00945	0.2494	0.0493	40.372	0.2872	1.2089	0.5234	31.953
T2Cd100	T	Cd	B	0.00945	0.2108	0.0417	38.758	0.3008	1.2107	0.5166	34.482
T2Cd100	T	Cd	B	0.00945	0.3852	0.0762	42.322	0.2967	1.2577	0.5245	32.5603
T2Cd100	T	Cd	B	0.00945	0.3852	0.0762	36.807	0.2879	1.2593	0.5114	31.6447
T2Cd100	T	Cd	B	0.00945	0.3969	0.0775	40.596	0.3014	1.2601	0.5207	34.4024
T2Cd500	T	Cd	C	0.04725	0.3547	0.066	353.142	0.8264	3.1055	0.5934	306.4077

T2Cd500	T	Cd	C	0.04725	0.1593	0.0296	353.502	0.8294	3.1059	0.5235	285.9277
T2Cd500	T	Cd	C	0.04725	0.0586	0.0109	352.782	0.8294	3.1091	0.5945	322.6354
T2Cd500	T	Cd	C	0.04725	0.1909	0.0355	366.983	0.8266	3.1045	0.5213	304.9903
T2Cd500	T	Cd	C	0.04725	0.1909	0.0355	339.301	0.8298	3.1075	0.5917	300.8419
T2Cd500	T	Cd	C	0.04725	0.1937	0.0368	362.009	0.8304	3.1052	0.5196	324.6083
T2Cd1000	T	Cd	D	0.0945	0.0905	0.0231	686.332	0.592	1.4127	0.5005	99.3723
T2Cd1000	T	Cd	D	0.0945	0.2398	0.0613	686.594	0.5792	1.4133	0.4593	98.7508
T2Cd1000	T	Cd	D	0.0945	0.0195	0.005	686.07	0.5908	1.4138	0.5046	102.9821
T2Cd1000	T	Cd	D	0.0945	0.1166	0.0298	714.819	0.592	1.3931	0.461	100.3684
T2Cd1000	T	Cd	D	0.0945	0.1166	0.0298	657.846	0.5799	1.3934	0.5	94.1785
T2Cd1000	T	Cd	D	0.0945	0.122	0.0305	705.062	0.5908	1.3948	0.4586	101.2687
T2control	T	control	0	0	0.5988	0.1125	44.502	0.3348	1.0692	0.5697	142.3843
T2control	T	control	0	0	1.2097	0.2272	44.763	0.3239	1.0707	0.5724	141.3293
T2control	T	control	0	0	1.0061	0.189	44.241	0.3294	1.0694	0.5692	149.1246
T2control	T	control	0	0	0.9382	0.1762	44.547	0.3349	1.0627	0.5719	144.2794
T2control	T	control	0	0	0.9382	0.1762	44.457	0.3245	1.063	0.5677	140.1134
T2control	T	control	0	0	0.9694	0.1816	44.271	0.3295	1.0656	0.5707	158.9114
T2Zn0	T	Zn	A	0.28	0.8642	0.1888	1238.219	0.211	1.0995	0.6033	139.2501
T2Zn0	T	Zn	A	0.28	0.6044	0.1321	1238.245	0.212	1.1015	0.6277	137.9796
T2Zn0	T	Zn	A	0.28	0.5741	0.1254	1238.193	0.2124	1.1007	0.6022	141.3905
T2Zn0	T	Zn	A	0.28	0.6809	0.1488	1239.612	0.2114	1.0817	0.6303	139.54
T2Zn0	T	Zn	A	0.28	0.6809	0.1488	1236.826	0.2128	1.0821	0.6016	139.16
T2Zn0	T	Zn	A	0.28	0.6852	0.1546	1239.121	0.2127	1.0826	0.626	149.6915
T2Zn2	T	Zn	B	0.56	0.3214	0.0635	1396.06	0.459	1.2004	0.7403	101.572
T2Zn2	T	Zn	B	0.56	1.1584	0.2289	1396.692	0.4379	1.2012	0.7601	101.3904
T2Zn2	T	Zn	B	0.56	1.4235	0.2813	1395.428	0.4619	1.2035	0.7397	104.6074
T2Zn2	T	Zn	B	0.56	0.9678	0.1913	1397.36	0.4591	1.229	0.7621	102.5233
T2Zn2	T	Zn	B	0.56	0.9678	0.1913	1394.76	0.4386	1.2308	0.7389	101.0765
T2Zn2	T	Zn	B	0.56	0.9836	0.1979	1396.295	0.4622	1.2299	0.7585	110.6574
T2Zn4	T	Zn	C	1.12	0.5212	0.1136	1745.385	0.9938	1.8104	0.7871	94.8202
T2Zn4	T	Zn	C	1.12	0.2936	0.064	1745.857	0.9871	1.8123	0.8697	97.7161

T2Zn4	T	Zn	C	1.12	0.3417	0.0745	1744.913	0.9905	1.8149	0.7867	99.7188
T2Zn4	T	Zn	C	1.12	0.3855	0.0841	1746.148	0.9941	1.8322	0.8733	97.4184
T2Zn4	T	Zn	C	1.12	0.3855	0.0841	1744.623	0.9877	1.8343	0.7859	97.2063
T2Zn4	T	Zn	C	1.12	0.3957	0.0878	1745.422	0.9912	1.8336	0.8681	106.3776
T2Zn10	T	Zn	D	2.8	0.178	0.0632	1900.724	1.3639	1.9244	1.1314	47.0395
T2Zn10	T	Zn	D	2.8	0.103	0.0366	1901.238	1.4098	1.9265	1.0832	42.0733
T2Zn10	T	Zn	D	2.8	0.1267	0.045	1900.21	1.405	1.9253	1.1314	50.6526
T2Zn10	T	Zn	D	2.8	0.1359	0.0483	1901.555	1.3649	1.9454	1.0883	43.0251
T2Zn10	T	Zn	D	2.8	0.1359	0.0483	1899.894	1.4108	1.9458	1.1306	45.9251
T2Zn10	T	Zn	D	2.8	0.1369	0.0504	1900.765	1.4053	1.9467	1.0824	47.781
P2control	P	control	0	0	1.23	0.2999	14.715	0.7426	0.7589	0.5142	165.205
P2control	P	control	0	0	0.84	0.2048	14.801	0.7391	0.7579	0.5683	165.1449
P2control	P	control	0	0	1.071	0.2611	14.629	0.7348	0.755	0.5093	165.3422
P2control	P	control	0	0	1.047	0.2553	15.307	0.7874	0.8221	0.5658	165.2307
P2control	P	control	0	0	1.047	0.2553	14.123	0.7874	0.8198	0.4932	150.4464
P2control	P	control	0	0	1.0993	0.264	15.024	0.7847	0.8204	0.5461	173.4276
P2Cu1/2	P	Cu	A	0.13	0.0814	0.018	27.704	0.6785	0.6262	0.7057	148.9548
P2Cu1/2	P	Cu	A	0.13	0.0914	0.0202	28.599	0.7398	0.6267	0.6465	148.937
P2Cu1/2	P	Cu	A	0.13	0.0764	0.0169	26.809	0.7632	0.6284	0.7005	148.9843
P2Cu1/2	P	Cu	A	0.13	0.0827	0.0183	28.821	0.7418	0.6507	0.6639	148.9587
P2Cu1/2	P	Cu	A	0.13	0.083	0.0184	26.588	0.6708	0.6487	0.7325	148.8002
P2Cu1/2	P	Cu	A	0.13	0.0853	0.0191	27.554	0.6785	0.6465	0.6651	149.0982
P2Cu0	P	Cu	B	0.26	0.0514	0.0184	63.546	0.6713	0.7376	0.6655	141.9403
P2Cu0	P	Cu	B	0.26	0.0464	0.0166	63.861	0.6695	0.7444	0.6458	141.7629
P2Cu0	P	Cu	B	0.26	0.0334	0.012	63.231	0.6695	0.7473	0.6655	141.7861
P2Cu0	P	Cu	B	0.26	0.0433	0.0155	65.071	0.6964	0.7102	0.6458	141.8298
P2Cu0	P	Cu	B	0.26	0.0436	0.0156	62.021	0.6887	0.7076	0.6642	135.6889
P2Cu0	P	Cu	B	0.26	0.045	0.0158	64.247	0.71	0.7047	0.6609	147.0331
P2Cu2.5	P	Cu	C	0.65	0.0172	0.0049	212.696	0.5426	0.7406	0.6381	105.5551
P2Cu2.5	P	Cu	C	0.65	0.0322	0.0092	213.225	0.5416	0.7365	0.6239	105.8859
P2Cu2.5	P	Cu	C	0.65	0.0292	0.0084	212.167	0.5647	0.7341	0.6361	105.7616

P2Cu2.5	P	Cu	C	0.65	0.026	0.0074	231.031	0.6223	0.7513	0.6226	105.7342
P2Cu2.5	P	Cu	C	0.65	0.0262	0.0075	194.361	0.6201	0.7521	0.6811	102.6632
P2Cu2.5	P	Cu	C	0.65	0.0263	0.0076	224.39	0.6213	0.7456	0.6831	108.9932
P2Cu5	P	Cu	D	1.3	0.0142	0.0047	245.4771	0.6346	0.8213	0.9589	57.0266
P2Cu5	P	Cu	D	1.3	0.0082	0.0027	254.5335	0.6348	0.8233	0.9795	58.1591
P2Cu5	P	Cu	D	1.3	0.0042	0.0014	220.3367	0.6348	0.8223	0.9609	62.4011
P2Cu5	P	Cu	D	1.3	0.0087	0.0029	217.1134	0.6434	0.8278	0.9824	59.0474
P2Cu5	P	Cu	D	1.3	0.0088	0.0029	240.9934	0.6434	0.8305	0.957	59.6925
P2Cu5	P	Cu	D	1.3	0.009	0.003	248.8428	0.6456	0.8289	0.9782	65.1377
P2control	P	control	0	0	1.23	0.2999	2.749	0.7426	0.7589	0.5142	165.205
P2control	P	control	0	0	0.84	0.2048	2.839	0.7391	0.7579	0.5683	165.1449
P2control	P	control	0	0	1.071	0.2611	2.659	0.7348	0.755	0.5093	165.3422
P2control	P	control	0	0	1.047	0.2553	2.871	0.7874	0.8221	0.5658	165.2307
P2control	P	control	0	0	1.047	0.2553	2.626	0.7874	0.8198	0.4932	150.4464
P2control	P	control	0	0	1.0993	0.264	2.74	0.7847	0.8204	0.5461	173.4276
P2Pb0	P	Pb	A	0.025	0.2043	0.0393	7.825	0.8476	0.579	0.6329	214.8122
P2Pb0	P	Pb	A	0.025	0.2063	0.0397	8.149	0.8479	0.5734	0.5663	214.6337
P2Pb0	P	Pb	A	0.025	0.3393	0.0652	7.501	0.8479	0.5756	0.5952	213.45
P2Pb0	P	Pb	A	0.025	0.2497	0.048	7.978	0.9123	0.5716	0.566	214.2986
P2Pb0	P	Pb	A	0.025	0.2499	0.0481	7.672	0.9094	0.5703	0.6293	214.2319
P2Pb0	P	Pb	A	0.025	0.2616	0.0504	7.603	0.9038	0.5712	0.5612	232.4325
P2Pb10	P	Pb	B	0.25	0.094	0.0254	122.792	0.7474	0.6462	0.538	184.2055
P2Pb10	P	Pb	B	0.25	0.158	0.0427	122.805	0.7461	0.6456	0.5584	183.9649
P2Pb10	P	Pb	B	0.25	0.034	0.0092	122.779	0.7487	0.6471	0.5368	183.902
P2Pb10	P	Pb	B	0.25	0.0953	0.0258	151.127	0.8359	0.6361	0.5562	184.0241
P2Pb10	P	Pb	B	0.25	0.0953	0.0258	124.458	0.8295	0.6355	0.5324	180.7201
P2Pb10	P	Pb	B	0.25	0.0987	0.0268	141.669	0.8321	0.6364	0.5515	201.1495
P2Pb50	P	Pb	C	1.25	0.0173	0.0041	3465.937	0.6794	0.6646	0.4825	95.9707
P2Pb50	P	Pb	C	1.25	0.0143	0.0034	3465.946	0.6889	0.6626	0.4657	95.0332
P2Pb50	P	Pb	C	1.25	0.0023	0.0005	3465.928	0.6818	0.7147	0.4795	94.7334
P2Pb50	P	Pb	C	1.25	0.011	0.0026	3468.767	0.7513	0.7265	0.5063	95.2457

P2Pb50	P	Pb	C	1.25	0.0112	0.0027	3463.106	0.7481	0.7244	0.4729	87.3816
P2Pb50	P	Pb	C	1.25	0.0117	0.0028	3467.815	0.7455	0.7248	0.5066	99.4636
P2Pb100	P	Pb	D	2.5	0.0052	0.0014	4393.388	0.6468	0.8131	0.5344	93.045
P2Pb100	P	Pb	D	2.5	0.0132	0.0035	4393.455	0.631	0.8085	0.5398	93.4985
P2Pb100	P	Pb	D	2.5	0.0072	0.0019	4393.321	0.6383	0.8057	0.539	93.1886
P2Pb100	P	Pb	D	2.5	0.0083	0.0022	4395.073	0.6553	0.8674	0.5677	93.244
P2Pb100	P	Pb	D	2.5	0.0085	0.0023	4391.703	0.6406	0.8658	0.5386	84.5757
P2Pb100	P	Pb	D	2.5	0.0088	0.0023	4394.444	0.6394	0.8679	0.56	101.5207
P2control	P	control	0	0	1.23	0.2999	6.372	0.7426	0.7589	0.5142	165.205
P2control	P	control	0	0	0.84	0.2048	6.617	0.7391	0.7579	0.5683	165.1449
P2control	P	control	0	0	1.071	0.2611	6.127	0.7348	0.755	0.5093	165.3422
P2control	P	control	0	0	1.047	0.2553	7.105	0.7874	0.8221	0.5658	165.2307
P2control	P	control	0	0	1.047	0.2553	5.639	0.7874	0.8198	0.4932	150.4464
P2control	P	control	0	0	1.0993	0.264	6.615	0.7847	0.8204	0.5461	173.4276
P2Cr0	P	Cr	A	0.036	0.4322	0.0792	6.357	0.6845	0.4793	0.4465	181.6091
P2Cr0	P	Cr	A	0.036	0.5312	0.0973	6.545	0.6782	0.4798	0.5031	181.769
P2Cr0	P	Cr	A	0.036	0.5762	0.1055	6.169	0.6702	0.4784	0.4503	181.7438
P2Cr0	P	Cr	A	0.036	0.513	0.094	6.618	0.7178	0.4882	0.4969	181.7073
P2Cr0	P	Cr	A	0.036	0.5132	0.094	6.097	0.7209	0.4759	0.4622	171.04
P2Cr0	P	Cr	A	0.036	0.5368	0.0978	6.343	0.7146	0.4873	0.4926	197.9749
P2Cr5	P	Cr	B	0.18	0.2274	0.0429	4.538	0.7711	0.4735	0.557	185.4997
P2Cr5	P	Cr	B	0.18	0.1404	0.0265	4.568	0.7737	0.5019	0.5896	185.7921
P2Cr5	P	Cr	B	0.18	0.4144	0.0782	4.508	0.7703	0.5045	0.5492	187.0353
P2Cr5	P	Cr	B	0.18	0.2603	0.0491	4.675	0.8147	0.5019	0.5511	186.1091
P2Cr5	P	Cr	B	0.18	0.2606	0.0492	4.402	0.8239	0.5006	0.5605	179.1302
P2Cr5	P	Cr	B	0.18	0.2736	0.0513	4.6	0.8256	0.4996	0.5808	195.9157
P2Cr10	P	Cr	C	0.36	0.0473	0.0107	6.524	0.7594	0.5213	0.6849	155.3428
P2Cr10	P	Cr	C	0.36	0.0933	0.0211	6.542	0.757	0.5206	0.6591	155.4477
P2Cr10	P	Cr	C	0.36	0.1163	0.0263	6.506	0.7509	0.5191	0.6818	155.1467
P2Cr10	P	Cr	C	0.36	0.0853	0.0193	6.719	0.863	0.5421	0.656	155.3124
P2Cr10	P	Cr	C	0.36	0.0856	0.0194	6.328	0.9143	0.5413	0.6758	143.5313

P2Cr10	P	Cr	C	0.36	0.0871	0.0197	6.375	0.9049	0.5409	0.65	156.1514
P2Cr25	P	Cr	D	0.9	0.0823	0.0188	894.657	0.8641	1.04	0.7721	118.8467
P2Cr25	P	Cr	D	0.9	0.0393	0.009	894.858	0.8698	1.0394	0.753	119.0817
P2Cr25	P	Cr	D	0.9	0.0643	0.0147	894.456	0.8698	1.0455	0.7683	118.9003
P2Cr25	P	Cr	D	0.9	0.0617	0.0141	903.755	0.9143	1.0576	0.7498	118.9429
P2Cr25	P	Cr	D	0.9	0.0619	0.0142	885.56	0.914	1.0572	0.7591	109.8888
P2Cr25	P	Cr	D	0.9	0.0634	0.0143	888.391	0.9132	1.0577	0.7377	127.65
P2control	P	control	0	0	1.23	0.2999	2.909	0.7426	0.7589	0.5142	165.205
P2control	P	control	0	0	0.84	0.2048	2.968	0.7391	0.7579	0.5683	165.1449
P2control	P	control	0	0	1.071	0.2611	2.85	0.7348	0.755	0.5093	165.3422
P2control	P	control	0	0	1.047	0.2553	3.195	0.7874	0.8221	0.5658	165.2307
P2control	P	control	0	0	1.047	0.2553	2.623	0.7874	0.8198	0.4932	150.4464
P2control	P	control	0	0	1.0993	0.264	2.66	0.7847	0.8204	0.5461	173.4276
P2Ni0	P	Ni	A	0.025	0.5671	0.1226	52.507	0.8247	0.6394	0.5981	165.7995
P2Ni0	P	Ni	A	0.025	1.0351	0.2237	53.521	0.8296	0.638	0.5537	165.8465
P2Ni0	P	Ni	A	0.025	0.8951	0.1934	51.493	0.8296	0.639	0.5941	165.7622
P2Ni0	P	Ni	A	0.025	0.8323	0.1799	56.751	0.9111	0.6795	0.5533	165.8028
P2Ni0	P	Ni	A	0.025	0.8324	0.1799	48.263	0.908	0.6755	0.5858	155.8977
P2Ni0	P	Ni	A	0.025	0.8656	0.1876	48.664	0.9016	0.6738	0.5464	179.9363
P2Ni5	P	Ni	B	0.125	0.8511	0.2115	179.072	0.703	0.6566	0.6069	70.5096
P2Ni5	P	Ni	B	0.125	0.6641	0.165	179.324	0.7018	0.6554	0.5944	70.4426
P2Ni5	P	Ni	B	0.125	0.7411	0.1841	178.82	0.7043	0.655	0.6052	70.3128
P2Ni5	P	Ni	B	0.125	0.752	0.1868	201.639	0.6888	0.663	0.5938	70.4217
P2Ni5	P	Ni	B	0.125	0.7521	0.1869	156.505	0.6804	0.662	0.5968	66.1927
P2Ni5	P	Ni	B	0.125	0.7602	0.1877	163.775	0.6879	0.6653	0.5886	70.8651
P2Ni10	P	Ni	C	0.25	0.2863	0.06	316.76	0.6684	0.8698	0.7297	67.0506
P2Ni10	P	Ni	C	0.25	0.7933	0.1662	317.557	0.6664	0.8704	0.6261	67.0729
P2Ni10	P	Ni	C	0.25	0.2243	0.047	315.963	0.6671	0.8709	0.7258	66.829
P2Ni10	P	Ni	C	0.25	0.4343	0.091	341.765	0.6649	0.8673	0.6242	66.9842
P2Ni10	P	Ni	C	0.25	0.4346	0.0911	291.756	0.6475	0.8682	0.7203	62.0383
P2Ni10	P	Ni	C	0.25	0.4541	0.0931	299.294	0.6524	0.8676	0.6182	73.0733

P2Ni25	P	Ni	D	0.625	0.2173	0.053	370.6092	0.8296	0.9893	0.738	56.993
P2Ni25	P	Ni	D	0.625	0.1073	0.0262	371.5417	0.8314	0.9919	0.7423	57.0119
P2Ni25	P	Ni	D	0.625	0.1013	0.0247	369.6767	0.8305	0.9897	0.7417	56.8046
P2Ni25	P	Ni	D	0.625	0.1417	0.0346	399.8651	0.8281	0.982	0.7423	56.9365
P2Ni25	P	Ni	D	0.625	0.1419	0.0346	341.3545	0.8298	0.9835	0.7377	52.7325
P2Ni25	P	Ni	D	0.625	0.1448	0.0358	350.174	0.8288	0.9844	0.7395	62.1123
P2control	P	control	0	0	1.23	0.2999	4.925	0.7426	0.7589	0.5142	165.205
P2control	P	control	0	0	0.84	0.2048	4.937	0.7391	0.7579	0.5683	165.1449
P2control	P	control	0	0	1.071	0.2611	4.913	0.7348	0.755	0.5093	165.3422
P2control	P	control	0	0	1.047	0.2553	4.991	0.7874	0.8221	0.5658	165.2307
P2control	P	control	0	0	1.047	0.2553	4.859	0.7874	0.8198	0.4932	150.4464
P2control	P	control	0	0	1.0993	0.264	4.869	0.7847	0.8204	0.5461	173.4276
P2Cd0	P	Cd	A	0.0000945	0.7572	0.1809	5.138	0.732	0.462	0.4229	193.1515
P2Cd0	P	Cd	A	0.0000945	0.5922	0.1415	5.006	0.732	0.4627	0.4438	193.6803
P2Cd0	P	Cd	A	0.0000945	0.4762	0.1138	5.27	0.7313	0.4581	0.4184	193.6998
P2Cd0	P	Cd	A	0.0000945	0.6083	0.1453	5.727	0.7848	0.4857	0.4355	193.5105
P2Cd0	P	Cd	A	0.0000945	0.6085	0.1454	5.49	0.7898	0.4862	0.4075	181.3248
P2Cd0	P	Cd	A	0.0000945	0.6357	0.152	5.777	0.7848	0.4818	0.409	211.0341
P2Cd100	P	Cd	B	0.00945	0.0981	0.0225	283.544	0.6561	0.4792	0.5201	128.2584
P2Cd100	P	Cd	B	0.00945	0.2801	0.0643	284.147	0.6488	0.478	0.492	128.0273
P2Cd100	P	Cd	B	0.00945	0.1471	0.0338	282.941	0.644	0.4764	0.516	128.2642
P2Cd100	P	Cd	B	0.00945	0.175	0.0402	301.857	0.6506	0.5554	0.4893	128.1833
P2Cd100	P	Cd	B	0.00945	0.1751	0.0402	265.23	0.6481	0.5542	0.5003	117.4096
P2Cd100	P	Cd	B	0.00945	0.1753	0.0412	270.732	0.6449	0.5593	0.4734	138.4543
P2Cd500	P	Cd	C	0.04725	0.1241	0.0264	759.56	0.4985	0.5926	0.5448	71.2324
P2Cd500	P	Cd	C	0.04725	0.2161	0.046	761.828	0.4943	0.5921	0.5193	71.8226
P2Cd500	P	Cd	C	0.04725	0.1701	0.0362	757.292	0.4985	0.588	0.5409	70.9462
P2Cd500	P	Cd	C	0.04725	0.17	0.0362	763.201	0.496	0.6111	0.5148	71.3338
P2Cd500	P	Cd	C	0.04725	0.1701	0.0362	755.92	0.4943	0.6124	0.5253	71.0294
P2Cd500	P	Cd	C	0.04725	0.1734	0.0375	754.865	0.496	0.6052	0.4999	73.9164
P2Cd1000	P	Cd	D	0.0945	0.102	0.0308	783.186	0.7047	0.6591	0.5898	78.1734

P2Cd1000	P	Cd	D	0.0945	0.094	0.0284	783.969	0.6888	0.6591	0.488	73.7595
P2Cd1000	P	Cd	D	0.0945	0.074	0.0223	782.403	0.6953	0.6569	0.5856	76.8186
P2Cd1000	P	Cd	D	0.0945	0.09	0.0272	808.096	0.7262	0.6515	0.4833	76.2505
P2Cd1000	P	Cd	D	0.0945	0.09	0.0272	758.277	0.7171	0.6504	0.5549	75.6404
P2Cd1000	P	Cd	D	0.0945	0.0909	0.0284	765.797	0.7313	0.6441	0.5535	81.7815
P2control	P	control	0	0	1.23	0.2999	85.132	0.7426	0.7589	0.5142	165.205
P2control	P	control	0	0	0.84	0.2048	85.56	0.7391	0.7579	0.5683	165.1449
P2control	P	control	0	0	1.071	0.2611	84.704	0.7348	0.755	0.5093	165.3422
P2control	P	control	0	0	1.047	0.2553	91.712	0.7874	0.8221	0.5658	165.2307
P2control	P	control	0	0	1.047	0.2553	78.553	0.7874	0.8198	0.4932	150.4464
P2control	P	control	0	0	1.0993	0.264	80.318	0.7847	0.8204	0.5461	173.4276
P2Zn0	P	Zn	A	0.28	0.2174	0.0414	1298.67	0.6259	0.6559	0.5996	124.2432
P2Zn0	P	Zn	A	0.28	0.5744	0.1093	1303.216	0.6203	0.6526	0.5906	124.2846
P2Zn0	P	Zn	A	0.28	0.1444	0.0275	1294.124	0.6255	0.6528	0.597	124.3255
P2Zn0	P	Zn	A	0.28	0.3117	0.0593	1300.334	0.6708	0.717	0.5874	124.2844
P2Zn0	P	Zn	A	0.28	0.312	0.0594	1297.007	0.6712	0.7143	0.5877	121.9322
P2Zn0	P	Zn	A	0.28	0.3173	0.0615	1293.015	0.672	0.7147	0.5822	124.7475
P2Zn2	P	Zn	B	0.56	0.2473	0.0541	1998.376	0.669	0.9184	0.7699	111.4828
P2Zn2	P	Zn	B	0.56	0.2573	0.0563	2000.852	0.6694	0.9189	0.7945	110.4833
P2Zn2	P	Zn	B	0.56	0.1993	0.0436	1995.9	0.6694	0.918	0.7668	110.2429
P2Zn2	P	Zn	B	0.56	0.2343	0.0513	1998.915	0.7501	0.9121	0.7918	110.7363
P2Zn2	P	Zn	B	0.56	0.2346	0.0513	1997.836	0.7472	0.9089	0.7589	107.1801
P2Zn2	P	Zn	B	0.56	0.2356	0.0517	1995.54	0.7481	0.91	0.7866	113.4133
P2Zn4	P	Zn	C	1.12	0.1962	0.0472	2204.175	0.7788	1.1416	0.8589	82.4537
P2Zn4	P	Zn	C	1.12	0.0582	0.014	2204.189	0.775	1.1378	0.9018	82.5456
P2Zn4	P	Zn	C	1.12	0.1682	0.0405	2204.161	0.775	1.1382	0.8538	82.5256
P2Zn4	P	Zn	C	1.12	0.1407	0.0339	2205.76	0.8219	1.3173	0.8988	82.5083
P2Zn4	P	Zn	C	1.12	0.1408	0.0339	2202.59	0.819	1.3148	0.8455	77.6767
P2Zn4	P	Zn	C	1.12	0.1476	0.0354	2203.105	0.819	1.3114	0.9045	88.8947
P2Zn10	P	Zn	D	2.8	0.0092	0.003	2314.384	1.1966	1.6938	1.1351	70.0857
P2Zn10	P	Zn	D	2.8	0.0152	0.0049	2314.399	1.1973	1.6958	1.1155	70.1638

P2Zn10	P	Zn	D	2.8	0.0062	0.002	2314.369	1.1991	1.6945	1.1357	70.1467
P2Zn10	P	Zn	D	2.8	0.01	0.0032	2316.048	1.2121	1.6686	1.1121	70.1321
P2Zn10	P	Zn	D	2.8	0.0102	0.0033	2312.72	1.2129	1.6688	1.1335	66.0252
P2Zn10	P	Zn	D	2.8	0.0106	0.0034	2313.26	1.214	1.6688	1.1117	75.5605
