# Supplementary material for: Production of Antioxidant Molecules in Polygonum aviculare (L.) and Senecio vulgaris (L.) under Metal Stress: A Possible Tool in the Evaluation of Plant Metal Tolerance
Source: Int J Mol Sci. 2020 Oct 3;21(19):7317. doi: 10.3390/ijms21197317 (PMC7582341; doi:10.3390/ijms21197317)
Supplement: Supplementary file 1 [file ijms-21-07317-s001.zip › Supplementary Table S3.pdf]

Supplementary Table S3. Spearman correlation coefficients (ϱ) between shoot metal concentration and plant biological parameters. Non-significant correlations (positive values) or anti-correlations (negative values), corresponding to ∣ϱ∣ < 0.300, are highlighted in red. Poorly significant values (0.301 < ∣ϱ∣ < 0.500), are highlighted in orange. **Excellent** values (∣ϱ∣ > 0.701) are in **black** color; the remaining good correlation/anticorrelation values (0.501 < ∣ϱ∣ < 0.700) are highlighted in blue. Populations: B, Bologna urban; M, Milan urban; N, Bologna woodland; T, Milan woodland; P, serpentine. A. activity: antioxidant activity, P. pigments: photosynthetic pigments (sum of chlorophyll *a*, chlorophyll *b*, carotenoid levels).

	P. aviculare	Cu	Pb	Cr	Ni	Cd	Zn		*S. vulgaris*	Cu	Pb	Cr	Ni	Cd	Zn
population B	**Shoot FW**	-0.890	-0.859	-0.850	-0.904	-0.835	-0.901	population B	**Shoot FW**	-0.976	-0.928	-0.841	-0.683	-0.920	-0.877
	**Flavonoids**	0.786	0.923	0.691	0.950	0.446	0.823		**Flavonoids**	0.836	0.570	0.890	0.584	0.749	0.723
	**Polyphenols**	0.638	0.770	0.881	0.678	0.632	0.575		**Polyphenols**	0.709	0.739	0.896	0.628	0.166	0.600
	**A. activity**	0.281	0.752	0.615	0.836	0.235	0.825		**A. activity**	0.768	0.868	0.298	0.143	0.710	0.683
	**P. pigments**	-0.908	-0.852	-0.540	-0.871	-0.117	-0.830		**P. pigments**	-0.839	-0.509	-0.600	-0.576	-0.720	-0.873
population M	**Shoot FW**	-0.714	-0.858	-0.740	-0.787	-0.829	-0.742	population M	**Shoot FW**	-0.807	-0.904	-0.874	-0.587	-0.807	-0.711
	**Flavonoids**	0.936	0.940	0.205	0.915	0.733	0.484		**Flavonoids**	0.612	0.342	0.664	0.414	0.531	0.761
	**Polyphenols**	0.646	0.919	0.365	0.632	0.821	0.695		**Polyphenols**	0.708	0.952	0.862	0.928	0.864	0.891
	**A. activity**	0.599	0.880	0.898	0.925	0.146	0.270		**A. activity**	0.928	0.270	0.877	0.329	0.881	0.746
	**P. pigments**	-0.587	-0.456	-0.289	-0.476	-0.623	-0.683		**P. pigments**	-0.859	-0.729	-0.41	-0.906	-0.758	-0.898
population N	**Shoot FW**	-0.935	-0.875	-0.805	-0.967	-0.965	-0.755	population N	**Shoot FW**	-0.867	-0.955	-0.786	-0.872	-0.883	-0.877
	**Flavonoids**	0.608	0.114	0.801	0.564	0.501	0.853		**Flavonoids**	0.667	0.663	0.655	0.478	0.442	0.863
	**Polyphenols**	0.879	0.813	0.836	0.866	0.856	0.828		**Polyphenols**	0.643	0.890	0.569	0.131	0.560	0.611
	**A. activity**	0.122	0.238	0.503	0.500	0.660	0.657		**A. activity**	0.216	0.131	0.211	0.325	0.770	0.578
	**P. pigments**	-0.408	-0.501	-0.328	-0.733	-0.823	-0.512		**P. pigments**	-0.807	-0.582	-0.270	-0.870	-0.518	-0.824
population T	**Shoot FW**	-0.910	-0.893	-0.844	-0.656	-0.895	-0.916	population T	**Shoot FW**	-0.883	-0.958	-0.893	-0.926	-0.897	-0.741
	**Flavonoids**	0.946	0.849	0.956	0.170	0.472	0.677		**Flavonoids**	0.905	0.676	0.765	0.653	0.585	0.862
	**Polyphenols**	0.585	0.662	0.493	0.162	0.116	0.856		**Polyphenols**	0.838	0.671	0.959	0.514	0.848	0.956
	**A. activity**	0.695	0.877	0.692	0.370	0.684	0.696		**A. activity**	0.967	0.924	0.576	0.182	0.661	0.985
	**P. pigments**	-0.902	-0.941	-0.41	-0.765	-0.931	-0.697		**P. pigments**	-0.948	-0.903	-0.257	-0.863	-0.125	-0.929
population P	**Shoot FW**	-0.908	-0.853	-0.885	-0.931	-0.894	-0.846	population P	**Shoot FW**	-0.945	-0.935	-0.416	-0.913	-0.905	-0.911
	**Flavonoids**	0.816	0.859	0.669	0.487	0.126	0.343		**Flavonoids**	0.806	0.613	0.337	0.197	0.634	0.602
	**Polyphenols**	0.922	0.901	0.644	0.724	0.611	0.878		**Polyphenols**	0.333	0.294	0.692	0.616	0.119	0.868
	**A. activity**	0.929	0.891	0.836	0.822	0.650	0.960		**A. activity**	0.614	0.223	0.566	0.940	0.256	0.975
	**P. pigments**	-0.893	-0.824	-0.579	-0.940	-0.935	-0.959		**P. pigments**	-0.937	-0.620	-0.707	-0.842	-0.778	-0.963
